# Proceedings of the Addiction Health Services Research (AHSR) 2020: Virtual Conference: Part 2

**DOI:** 10.1186/s13722-020-00208-4

**Published:** 2020-12-30

## A1 “Patient characteristics associated with admission to low-safety inpatient psychiatric facilities: evidence of racial inequities” (AW01)

### Morgan C. Shields

#### Lead Author Affiliation: Center for Mental Health, Department of Psychiatry, Perelman School of Medicine, University of Pennsylvania, 3400 Civic Center Blvd, Philadelphia, PA 19104, USA

##### **Correspondence:** Morgan C. Shields (shmorg@upenn.edu)

*Addict Sci Clin Pract* 2020, **15(Suppl 2)**:A1

**Background:** I examined patient demographic, clinical, payment, and geographic factors associated with admission to low-safety inpatient psychiatric facilities.

**Methods:** Massachusetts all-payer 2017 discharge data (N = 39,128) were linked to facility-level indicators of safety (N = 38). A composite of safety performance was constructed by averaging standardized measures of restraint and seclusion, as well as five-year (2014–2018) averages of overall, substantiated, and abuse-related (verbal, physical, sexual abuse) complaints per 1,000 discharges (• = 0.73). This composite informed the grouping of high (top 20%), middle, and low-safety (bottom 20%) performers. I first examined unadjusted differences across safety groups, as well as differences in bypass patterns across racial and ethnic groups. I then fit a series of multinomial regression models, adding payment and geography separately.

**Results:** Outstanding factors independently associated with admission to low-safety facilities were being a racial or ethnic minority compared to White patient (relative risk ratio [RRR] for non-Hispanic Black = 1.7, 95% CI = 1.5–2.0; non-Hispanic Asian = 5.6, 95% CI = 3.6–8.7; non-Hispanic “other” race = 2.2, 95% CI = 1.7–2.7; Hispanic/Latinx = 1.3, CI = 1.1–1.5), and not having private insurance (RRR for uninsured/self-pay = 2.4, CI = 1.6–3.6, Medicaid = 1.8, CI = 1.6–2.0, Medicare = 1.3, CI = 1.2–1.5). Several other factors were independently associated with admission to low-safety facilities, such as substance use disorder other than alcohol, proximity, severity, schizophrenia/psychosis, homelessness, and younger age.

**Conclusion:** There were considerable racial and ethnic inequities in admission to low-safety inpatient psychiatric facilities even after accounting for clinical, geographic, and payment characteristics. Future research should further examine quality variation and outcomes, as well as how community-based referrals, mode of transport (e.g., police, self), and deliberate steering and selection affect admissions and outcomes.

## A2 “Desafíos Enredados (entangled challenges): an intersectional examination of risk factors linked to substance use among sexual and gender minority Latinx individuals” (AW02)

### Benjamin F. Shepherd and Paula M. Brochu

#### Lead Author Affiliation: Nova Southeastern University, 3301 College Ave, Fort Lauderdale, FL 33314, USA

##### **Correspondence:** Benjamin F. Shepherd (bs1759@mynsu.nova.edu)

*Addict Sci Clin Pract* 2020, **15(Suppl 2)**:A2

**Background: **Mounting research shows sexual and gender minority (SGM) and Latinx populations experience an alarming array of health disparities, including higher rates of substance use. Substance use is linked to not only substance use disorders, but also health risk behaviors that could lead to HIV infection. However, a dearth of knowledge surrounds the health and resilience of individuals who possess both marginalized identities. As such, the purpose of this systematic literature review is to increase understanding and awareness of factors that elevate SGM Latinx individuals’ risk of substance use.

**Methods:** Guided by PRISMA criteria, a search of peer-reviewed, English and Spanish language articles was conducted using three databases (APA PsycInfo, Pubmed, and Google Scholar). Studies were included if they met the following criteria: (a) assessed individuals who self-identified as SGM in addition to Latinx/Hispanic and (b) assessed variables in association with substance use.

**Results:** Across 26 studies, a total of 28 risk factors were identified. These findings show SGM Latinx individuals face unique (e.g., minority stressors, family rejection, acculturation, machismo, legal status) and common (e.g., depression, anxiety, disordered eating attitudes and behaviors, childhood abuse) challenges that increase their odds of problematic substance use. For example, SGM Latinx individuals may abuse alcohol and/or drugs as a way of coping with negative cultural messages related to their sexuality, gender identity, race/ethnicity, HIV status, mental health, or weight. Because many of the identified risk factors are interrelated (e.g., bullying, discrimination, internalized stigma, mental health), they may intersect with one another to worsen substance use outcomes.

**Conclusion: **This review provides intersectional insights into the risk factors linked to substance use among SGM Latinx individuals. Such information paves the way for future culturally responsive health research, education, and policy, and can inform assessments and interventions to prevent and treat substance use in this growing yet underserved population.

## A3 “Ensuring access to quality substance use disorder treatment for Medicaid enrollees: a qualitative study of diverse stakeholders’ perspectives” (AW03)

### Jenny Zhen-Duan, Marie Fukuda, Irene Falgas-Bagué, and Margarita Alegría

#### Lead Author Affiliation: Massachusetts General Hospital, 55 Fruit Street, Boston, MA 02114, USA

##### **Correspondence:** Jenny Zhen-Duan (jzhen-duan@mgh.harvard.edu)

*Addict Sci Clin Pract* 2020, **15(Suppl 2)**:A3

**Background:** Policies such as transitioning Medicaid beneficiaries from fee-for-service to managed care plans and increasing behavioral health coverage were crafted to improve SUD care, yet treatment rates have remained the same. The shifting landscape and structure of Medicaid may affect treatment for low-income populations, who already face greater barriers to quality and adequate SUD care. Using a policy implementation research approach, our goal was to explore different stakeholders’ perspectives on (1) the effects on SUD care when transitioning from Medicaid fee-for-service to managed care and (2) remaining barriers and facilitators to receiving or providing SUD quality care through managed care.

**Method: **Semi-structured, in-depth, qualitative interviews were conducted with stakeholders in throughout New York State (NYS). Patients were interviewed in outpatient and harm-reduction centers in New York City whereas non-patient stakeholders were interviewed over the phone.

**Results:** Interviewees (N = 40) were policy leaders (n = 13), clinicians (n = 12), plan administrators (n = 5), and patients (n = 10), all involved with different aspects of Medicaid SUD treatment. Three major themes emerged after using a thematic analysis framework: (1) transition to managed care has been beneficial, yet certain policies hinder Medicaid enrollment and deter quality of care; (2) quality of care is poorer for those with dual diagnoses, older adults, and linguistic minorities; and (3) NYS quality metrics do not adequately capture treatment quality.

**Conclusions:** Improvements should be focused on greater interinstitutional coordination and communication between stakeholders, including patients’ input in policies and practices affecting SUD care, increasing patient navigation resources, providing low barrier psychiatric services and increasing resources for older adults and linguistic minorities. Opportunities for NYS include creating metrics to measure more meaningful outcomes and tying insurance reimbursements based on metrics reflecting patients’ quality of life improvements.

## A4 “The implementation & sustainment facilitation (ISF) strategy: cost and cost-effectiveness results from a 39-site cluster randomized trial integrating substance use services in AIDS services organizations” (AW04)

### Jesse M. Hinde, Bryan R. Garner, Colleen J. Watson, Rasika Ramanan, Elizabeth L. Ball, and Stephen J. Tueller

#### Lead Author Affiliation: RTI International, 3040 E Cornwallis Rd, Durham, NC 27709, USA

##### **Correspondence:** Jesse M. Hinde (jhinde@rti.org)

*Addict Sci Clin Pract* 2020, **15(Suppl 2)**:A4

**Background:** Substance use among people with HIV is both prevalent and problematic, yet the integration of substance use treatment within HIV service settings is rare. The Substance Abuse Treatment to HIV Care Project was funded to test an organization-focused strategy called Implementation & Sustainment Facilitation (ISF) as an adjunct to the Addiction Technology Transfer Center (ATTC) strategy. This presentation presents the cost and cost-effectiveness results from this cluster randomized implementation experiment.

**Methods: **Thirty-nine AIDS Service Organizations (ASO) and two brief intervention (BI) staff per ASO (N = 78) were randomized to receive (1) the ATTC strategy (ATTC only) or (2) the ATTC strategy plus the ISF strategy (ATTC + ISF). We estimated costs using primary data on the time spent on the ATTC strategy, on the ISF strategy, and implementing BIs. Salary information was collected via staff surveys. We conducted a cost-effectiveness analysis at the staff level on the number of BIs implemented, the overall MIBI quality scores achieved, and total average patient days abstinent.

**Results:** Per BI staff costs were 3176 for the ATTC strategy and 5752 for the ATTC + ISF strategy, resulting in an incremental cost of 2576. The incremental difference was approximately 4 for BIs delivered and 780 for MIBI quality scores achieved, yielding incremental cost effectiveness ratios of 644 and 3, respectively. Patients receiving a BI in the ATTC + ISF condition averaged 60 more days of abstinence at follow—upper BI staff, yielding an incremental cost effectiveness ratio of 42.

**Discussion:** The ISF strategy was found to be a cost-effective adjunct to the ATTC’s current state-of-the-art implementation strategy. The current finding is important given that it suggests ISF as a promising strategy to improve the integration of substance use treatment within ASOs.

## A5 “Variation in US drug overdose mortality by Hispanic heritage group, 2017” (AW05)

### Manuel Cano

#### Lead Author Affiliation: University of Texas at San Antonio, 1 UTSA Circle, San Antonio, TX 78249, USA

##### **Correspondence:** Manuel Cano (manuel.cano@utsa.edu)

*Addict Sci Clin Pract* 2020, **15(Suppl 2)**:A5

**Background:** The drug overdose crisis has devastated communities across the United States (US), yet relatively little is known about the recent impact on diverse Hispanic populations. The study examined variation in drug overdose mortality across Hispanic heritage subgroups.

**Methods:** Death certificate data were obtained from the 2017 Multiple Cause of Death restricted-access file from the National Center for Health Statistics. The study focused on the 4935 drug overdose deaths in the US (50 states and DC) in 2017 among decedents identified as of Mexican, Puerto Rican, Cuban, Dominican, or Central or South American heritage. Age-adjusted drug overdose mortality rates were computed using direct standardization, with 2017 American Community Survey 1-year estimates utilized for population denominators. Two-tailed z tests were used to assess evidence of statistical significance of differences.

**Results:** Of all Hispanic heritage groups, the highest age-adjusted drug overdose mortality rate in the US in 2017 was observed among Puerto Ricans (29.0 per 100,000, 95% CI 27.6–30.4), with a rate 6% significantly higher than the rate in Non-Hispanic Whites. Significantly lower rates were observed among various other Hispanic heritage groups, ranging from 3.8 (95% CI 3.3–4.3) among Central Americans to 10.1 (95% CI 8.8–11.6) among Dominicans. In the Central and South American heritage groups, the drug overdose decedents were concentrated in relatively younger ages; in contrast, 41.1% of Puerto Rican drug overdose decedents were between the ages of 45 and 64. Synthetic opioids represented the drug category involved in the highest proportion of overdose deaths for all Hispanic heritage groups, except for the Mexican heritage group, in which psychostimulants were involved in the highest proportion of overdose deaths.

**Conclusion:** Results highlight substantial variation in drug overdose mortality rates among different Hispanic heritage groups, suggesting that national rates for Hispanics overall obscure higher-risk subgroups such as stateside Puerto Ricans.

## A6 “Patterns of psychosocial and behavioral therapy received in conjunction with buprenorphine treatment” (AW06)

### Hillary Samples, Arthur R. Williams, Mark Olfson, and Stephen Crystal

#### Lead Author Affiliation: Rutgers University, 683 Hoes Lane West, Piscataway, NJ, 08854, USA

##### **Correspondence:** Hillary Samples (h.samples@rutgers.edu)

*Addict Sci Clin Pract* 2020, **15(Suppl 2)**:A6

**Background:** Current evidence indicates that medication is the most effective treatment for opioid use disorder. However, research on the effectiveness of psychosocial and behavioral therapy is mixed, with some studies showing no added benefit beyond medication treatment with buprenorphine and others showing improvements in treatment retention and outcomes. The goal of this study was to identify trajectories of psychosocial and behavioral services received during the first 6 months of buprenorphine treatment and to examine the association of therapy patterns with patient characteristics and treatment discontinuation.

**Methods:** We analyzed 2013–2018 MarketScan Multi-State Medicaid claims to define longitudinal patterns of psychosocial and behavioral therapy among enrollees with buprenorphine treatment. The sample included adults 18–64 years at buprenorphine initiation with treatment episodes ^3^30 days (n = 40,969). Group-based trajectory models estimated distinct patterns of psychosocial/behavioral therapy. Multinomial logistic regression estimated associations between patient characteristics and therapy patterns. Cox proportional hazards regression estimated time to buprenorphine discontinuation across therapy groups.

**Results:** We identified three trajectories of psychosocial and behavioral therapy services received in conjunction with buprenorphine treatment: None (70.4%), Low-intensity (20.4%), and High-intensity (9.2%). In the first 6 months of buprenorphine treatment, the average number of therapy services was 10.0 for the low-intensity group and 39.5 for the high-intensity group. Compared to the group without psychosocial/behavioral services, low- and high-intensity therapy groups were more likely to have comorbid mental health and substance use disorder diagnoses, all-cause emergency department services and medically treated opioid overdose at baseline. However, the hazard of buprenorphine treatment discontinuation was lower for those with low- (HR = 0.81; p < 0.001) and high-intensity therapy patterns (HR = 0.66; p < 0.001) compared to those without therapy services.

**Conclusion: **Behavioral interventions received in conjunction with buprenorphine medication may improve treatment retention for patients with high-risk clinical profiles.

## A7 “Health-related quality of life and opioid use disorder pharmacotherapy: a secondary analysis of a clinical trial” (TR01)

### Ali Jalali, Danielle A. Ryan, Philip J. Jeng, Kathryn E. McCollister, Jared A. Leff, Joshua D. Lee, Edward V. Nunes, Patricia Novo, John Rotrosen, Bruce R. Schackman, and Sean M. Murphy

#### Lead Author Affiliation: Weill Cornell Medical College, 1300 York Ave, New York, NY 10065, USA

##### **Correspondence:** Ali Jalali (alj4004@med.cornell.edu)

*Addict Sci Clin Pract* 2020, **15(Suppl 2)**:A7

**Background:** To examine the health-related quality-of-life (HRQoL) of persons with opioid use disorder (OUD) seeking treatment in an inpatient detoxification or short-term residential setting; continuing treatment as outpatients.

**Methods:** We conducted a secondary analysis of data from a clinical trial (N = 508) where participants were randomized to extended-release naltrexone or buprenorphine-naloxone for the prevention of opioid relapse. We used a generalized structural equation regression mixture model to identify associations of HRQoL (EQ-5D) trajectories, including latent characteristics, over the 24-week trial and 36-week follow-up period, among participants who reported HRQoL beyond baseline. This novel framework accounted for baseline and time-varying characteristics, while simultaneously identifying latent classes.

**Results:** We identified two subpopulations: HRQoL “pharmacotherapy responsive” (82.3%) and HRQoL “characteristic sensitive” (17.7%). The pharmacotherapy responsive subpopulation was characterized by a short-term HRQoL improvement and then stable HRQoL over time, and by a positive association between HRQoL and receiving pharmacotherapy in the past 30 days. The characteristic sensitive subpopulation was characterized by an initial improvement in HRQoL with a gradual decline over time, and no significant HRQoL response to pharmacotherapy. HRQoL changes over time in this subpopulation were more influenced by baseline demographic, socioeconomic, and psychosocial characteristics.

**Conclusion:** Our findings suggest that while HRQoL may be improved and sustained through targeted efforts to promote use of pharmacotherapy for many persons with OUD, an identifiable subpopulation may require additional services that address socioeconomic and psychosocial issues to achieve HRQoL benefits. Our analysis provides insight for improving individualized care for persons with opioid use disorder seeking treatment.

## A8 “Using machine learning to advance disparities research: subgroup analyses in access to opioid treatment” (TR02)

### Yinfei Kong, Jia Zhou, Zemin Zheng, Hortensia Amaro, and Erick Guerrero

#### Lead Author Affiliation: California State University, Fullerton, 800N State College Blvd, Fullerton, CA 92831, USA

##### **Correspondence:** Yinfei Kong (yikong@fullerton.edu)

*Addict Sci Clin Pract* 2020, **15(Suppl 2)**:A8

**Background:** To operationalize an intersectionality conceptual framework using a novel statistical approach and with these efforts improve estimation of disparities in access to treatment beyond race.

**Methods:** We analyzed a sample of 40,943 treatment episodes collected in 2015 in the state of Maryland. These data are from the Treatment Episodes Data Survey (TEDS-A). Study Design We conducted a retrospective subgroup analysis using a two-step approach called virtual twins. In step 1, we trained a classification model that gives the probability of waiting long (a week or more). In step 2, we identified the subgroups with higher probability difference of waiting long due to race. We tested five classification models for step 1 by simulation and identified the model with improved estimation. Client data were collected during personal interviews at admission and discharge.

**Results:** Estimation using the Random Forest model was the most accurate for the first step of our subgroup analysis. The subgroup analysis suggests that the following six factors augmented racial disparities (i.e., African American versus White) in access to treatment (wait time): (1) 40 years of age or older, (2) receiving medication-assisted opioid treatment (methadone, buprenorphine or naloxone), (3) using only one kind of opioid, (4) having one or more prior treatment episodes, (5) no prior psychiatric problem and (6) primary income from retirement, pension or disability. The probability of waiting 1 week or longer for African Americans was 14% higher than for Whites with the same characteristics.

**Conclusions:** The methodology proposed in this study adds more nuance to the complexities of disparities research. The findings have implications for reducing healthcare disparities by addressing specific factors beyond race that promote disparities. Findings can help policy makers, healthcare administrators and providers address the factors that make subgroups vulnerable to wait longer to enter treatment.

## A9 “Mortality after prison release in Washington State: 2014–2019” (TR03)

### Allyson O'Connor, Jeanne M. Sears, and Deborah Fulton-Kehoe

#### Lead Author Affiliation: University of Washington, 1959 NE Pacific St, Seattle, WA 98195, USA

##### **Correspondence:** Allyson O'Connor (awoconno@uw.edu)

*Addict Sci Clin Pract* 2020, **15(Suppl 2)**:A9

**Background:** Individuals released from prison are at an increased risk of death compared to the non-incarcerated population, particularly from opioid and drug-related overdose. Reduced physiologic opioid tolerance after abstaining from use while in prison may contribute to the high risk of overdose seen in those released from prison, especially immediately after release. This study examined current trends in overdose and other leading causes of death for individuals released from Washington State prisons, compared to those reported in a similarly designed 2013 study (covering 1999–2009).

**Methods: **This retrospective cohort study linked data for 33,811 individuals released from Washington State prisons (2014–2018) to Washington State death files (2014–2019) to identify date and cause of death for individuals who died after release. We identified substance-related deaths, used mortality rates to identify leading causes of death, and compared overdose to non-overdose deaths. Hazard ratios for risk factors for all-cause, non-overdose, and overdose deaths were estimated using Cox proportional hazard regression.

**Results:** The all-cause mortality rate was 747 per 100,000 person-years (95% CI 699 to 800). Drug overdose was the leading cause of death (216 per 100,000 person-years; 95% CI 190 to 244). Psychostimulants (152 per 100,000 person-years; 95% CI 131 to 177) and opioids (138 per 100,000 person-years; 95% CI 118 to 161) were the most prevalent substances among substance-related deaths. The strongest risk factors for all-cause, non-overdose, and overdose deaths were being age 45+ at most recent release, having previous incarcerations prior to release, and drug-related convictions.

**Conclusions:** Using the 2013 study findings for comparison, all-cause mortality rates changed little for individuals released from prison; however, drug overdose mortality rose. In contrast to the 2013 study, this study found that psychostimulants (e.g., methamphetamines), rather than opioids, were the most common substance-related cause of death. This study provides updated mortality statistics to inform future efforts to reduce substance-related deaths post-release.

## A10 “Predicting initiation to treatment for youth on probation: a multi-level approach” (TR04)

### Sarah DeLucca, Steven Belenko, and Ralph B. Taylor

#### Lead Author Affiliation: Temple University, 1801N Broad St, Philadelphia, PA 19122, USA

##### **Correspondence:** Sarah DeLucca (sarah.delucca@temple.edu)

*Addict Sci Clin Pract* 2020, **15(Suppl 2)**:A10

**Background:** Juveniles under community supervision have a higher prevalence of substance use compared to the general population. Previous research has examined factors that predict treatment outcomes once treatment has been initiated; however, there is a gap in the literature regarding the factors that affect initiation of treatment, particularly after referral by the juvenile justice system. This study analyzes individual-level factors with site and state-level variation predicting initiation of treatment following referral from juvenile probation agencies.

**Methods:** Using the Behavioral Health Services Cascade framework of the Juvenile Justice-Translational Research on Interventions for Adolescents in the Legal System (JJ-TRIALS) multisite project, a series of mixed effects logistic regression models were estimated using two methods. Referral and treatment initiation data from two analytic methods addressing missing data from 3312 youth records in Method 1 and 5325 records in Method 2 collected from 22 counties across six states were analyzed. We examined between site variation and assessed the impacts of individual- and state-level factors on initiation to treatment after referral.

**Results:** Results from the mixed effects model indicate that being referred and initiating treatment varied significantly across sites in both Methods. A mixed model in Method 1 with individual-level characteristics indicated that youth with a higher substance use treatment need and youth with a higher supervision level are 1.98 and 1.74 times more likely to initiate treatment after referral, respectively. In Model 3 for Method 2 analyses, when controlling for state-level differences, youth with a higher level of supervision were 2.26 times more likely to initiate treatment following referral compared to those with a lower level.

**Conclusion:** Findings suggest that individual factors matter in predicting initiation, independent of state or site. Level of supervision is the most salient factor in a youth’s initiate of treatment in both missing data methods, however, need for treatment is also important although it was significant in Method 1 only. Understanding the differences in predictors allows partnering behavioral health and juvenile justice agencies to develop strategies to increase the likelihood that referred youth initiate treatment.

## A11 “Opioid dependence and associated healthcare utilization and cost in a privately insured spinal cord injury population” (TR05)

### Beatrice Ugiliweneza, Miriam Nuno, April Herrity, Dengzhi Wang, Mayur Sharma, Shawn Adams, Nicholas Khattar, Nicholas Dietz, Fabian C. Madrigal, Doniel Drazin, and Maxwell Boakye

#### Lead Author Affiliation: University of Louisville, 2301 S 3rd St, Louisville, KY 40292, USA

##### **Correspondence:** Beatrice Ugiliweneza (beatrice.ugiliweneza@louisville.edu)

*Addict Sci Clin Pract* 2020, **15(Suppl 2)**:A11

**Background:** Chronic neuropathic, myopathic and visceral pain are debilitating complications of Spinal Cord Injury (SCI) oftentimes resulting in long-term, high dose opioid use and eventual dependence. To (1) evaluate predictors of opioid dependence after SCI; and (2) evaluate healthcare use and cost among different pre-post opioid dependence patterns.

**Methods:** Incident adult SCI cases were extracted from MarketScan (2000–2018) and followed for 15 months. Opioid dependence was flagged pre- (12 months) and post-SCI (3–15 months). Chronic neuropathic, myopathic and visceral pain was screened post-SCI. We evaluated factors explaining post-SCI dependence; then, looked at healthcare utilization and associated cost compared between prior non-dependent individuals who remained dependent (ND_ND), prior non-dependent who became dependent (ND_D), prior dependent who became non-dependent (D_ND) and prior dependent who remained dependent (D_D).

**Results:** The cohort was 56 years old on average (SD = 20), 53% males, 49% commercial insurance, 70% one or more comorbidities, 57% traumatic and 48% cervical injuries. Post-SCI, pain was recorded in 34% of opioid dependent compared to 16% non-dependent (p < 0.0001). Prior-dependent SCI individuals had 13 times higher odds of becoming dependent after injury (OR: 13, 95% CI 12–14) and post-SCI chronic pain was associated with a twofold odds compared to those who did not (OR: 2.2, 95% CI 2.0–2.4). In 3–15 months after SCI, after adjusting to demographics, comorbidities, degree of injury and pain, ND_D, D_ND, D_D had higher emergency room admissions (respectively OR: 1.874, 1.358, 2.440, p < 0.05), outpatient visits (respectively Estimate Ratio (ER): 1.637, 1.137, 1.161, p < 0.05), medication refills (respectively ER: 2.898, 1.459, 3.343, p < 0.05) and overall payments (respectively ER: 2.876, 1.217, 2.778, p < 0.05) compared to ND_ND.

**Conclusion:** After SCI, opioid dependence is associated with high healthcare utilization and cost. Those who are dependent prior to injury are more than 10 times likely to become dependent after and are heavier consumers of health care.

## A12 “Prescription patterns of adjuvant pain medications following opioid supply restriction law in Florida: an interrupted time series analysis” (TR06)

### Yun Shen, Juan M. Hincapie-Castillo, Scott M. Vouri, Marvin A. Dewar, Jill M. Sumfest, and Amie J. Goodin

#### Lead Author Affiliation: University of Florida, 1225 Center Drive Gainesville, FL 32610 HPNP Building, Rm 3334, USA

##### **Correspondence:** Yun Shen (yunshen@ufl.edu)

*Addict Sci Clin Pract* 2020, **15(Suppl 2)**:A12

**Background:** In Florida, House Bill 21 (HB21) was implemented in July 2018 to limit prescriptions of Schedule II opioids for acute pain patients to a 3-day supply. In response to restrictions to opioid prescriptions, drug utilization patterns of commonly co-prescribed medications might shift among chronic pain patients. Currently, little is known about the unintended impacts of opioid supply policy restrictions on adjuvant medication use.

**Methods:** We obtained prescription claims for medications dispensed from 1/1/2015 to 6/31/2019 from a health plan serving a large Florida employer. Interrupted time series analyses were conducted to compare pre and post-implementation changes in mean monthly number of users and prescriptions per 1000 enrollees for adjuvant medications: gabapentinoids, benzodiazepines, and muscle relaxants.

**Results: **There was a significant decrease in the mean monthly proportion of benzodiazepines users (17.37·1.26 vs. 14.12·0.61) and number of prescriptions (30.25·2.76 vs. 25.34·2.00) per 1000 patients. There were no significant changes in the mean monthly proportion of gabapentinoids users (9.01·0.35 vs. 9.71·0.50), gabapentinoid prescriptions (19.68·1.40 vs. 22.77·2.08), muscle relaxants users (13.31·0.67 vs. 12.33·0.97), or muscle relaxant prescriptions (23.41·1.91 vs. 22.45·2.29) per 1000 patients. Adjusting for key variables, there was an immediate 6% increase in monthly proportion of gabapentinoids users (RR: 1.06, 95% CI 1.02, 1.11) and an immediate 11% increase in gabapentinoid prescriptions (RR: 1.11, 95% CI 1.04, 1.18) per 1000 patients. Additionally, there was a 7% immediate reduction for monthly proportion of benzodiazepines users (RR: 0.93, 95% CI 0.89, 0.97), and a significant 15% reduction in trend was observed in monthly proportion of muscle relaxants users (RR: 0.98, 95% CI 0.97, 0.99; 0.83, 95% CI 0.77, 0.90) after the HB21 enactment.

**Conclusion:** Following the Florida opioid restriction law for acute pain, there were increased number of patients and prescriptions for gabapentinoids, however fewer patients received benzodiazepines and muscle relaxants in the post-implementation period.

## A13 “Adapting a peer-delivered behavioral activation intervention to support retention in methadone maintenance treatment for a low-income, minority population” (TR07)

### Mary B. Kleinman, Julia W. Felton, Christopher J. Seitz-Brown, Valerie D. Bradley, Annabelle Belcher, Melanie Bennett, Aaron Greenblatt, and Jessica F. Magidson

#### Lead Author Affiliation: University of Maryland, College Park, College Park, MD 20742, USA

##### **Correspondence:** Mary B. Kleinman (mkleinm@umd.edu)

*Addict Sci Clin Pract* 2020, **15(Suppl 2)**:A13

**Background:** Although estimates of opioid-related fatalities in the United States indicate a decrease from 2017 to 2018, deaths associated with the opioid epidemic continue to rise among low-income and minority populations. Despite efficacy of medication for opioid use disorder (MOUD), these populations are vulnerable to poor treatment outcomes. Peer recovery coaches (PRCs), individuals with lived experience of substance use and recovery, are well-positioned to engage vulnerable patients. Traditionally, PRCs have focused on bridging to care rather than delivering interventions themselves. This study used qualitative methods to solicit feedback on feasibility and acceptability of PRC-delivered Behavioral Activation (BA) to support retention in MOUD by increasing positive reinforcement.

**Methods: **This study was conducted at a community-based drug treatment center that serves low-income, minority patients and reports an average 49% retention at 6 months post-treatment initiation. We recruited patients and staff as well as PRCs who work across the city. Semi-structured interviews and focus groups inquired about feasibility and acceptability of a BA intervention, recommendations for adaptation for the target population, and comfort working with a peer in the context of MOUD.

**Results:** Participants (n = 20) had a mean age of 48.4 (SD = 10.0), were 70% male, and 60% Black or African American. Staff and PRC participants (n = 12) had a mean age of 49.2 (SD = 0.7), were 42% male, 75% Black or African American, with an average of 9.6 years working in substance use treatment. Participants shared that PRC-delivered BA could be feasible and acceptable with adaptations, including emphasis on PRC-led/taught activities. They described common challenges associated with unstructured time, for which BA could be particularly relevant.

**Conclusions:** Improving MOUD outcomes is a national priority that must be met with cost-effective, sustainable strategies to support individuals in treatment. Qualitative feedback suggests PRCs may be effective in this effort and our research findings inform an upcoming PRC-delivered BA trial.

## A14 “Improving transitions of care for patients initiated on buprenorphine from the emergency department” (TR08)

### Callan E. Fockele, Herbie C. Duber, Brad Finegood, Sophie C. Morse, and Lauren K. Whiteside

#### Lead Author Affiliation: Harborview Medical Center, 325 Ninth Avenue Seattle WA 98104-2499, USA

##### **Correspondence:** Callan E. Fockele (cfockele@uw.edu)

*Addict Sci Clin Pract* 2020, **15(Suppl 2)**:A14

**Background:** Opioid use disorder (OUD) is on the rise nationwide with increasing emergency department (ED) visits and deaths secondary to overdose. Although previous research has shown that patients who are started on buprenorphine in the ED have increased engagement in addiction treatment, access to on-demand medications for OUD is still limited, in part because of the need for outpatient linkages to care. The objective of this study is to describe emergency and outpatient providers’ perception of local barriers to transitions of care for ED-initiated buprenorphine patients.

**Methods: **Purposive sampling was used to recruit key stakeholders, who identified as physicians, addiction specialists, and hospital administrators, from 10 EDs and 11 outpatient clinics in King County, Washington. Twenty-one interviews were recorded and transcribed, and then coded by two team members in order to verify accuracy of the thematic analysis. Interview guides and coding were informed by the Consolidated Framework for Implementation Research (CFIR), which provides a structure of domains and constructs associated with effective implementation of evidence-based practice.

**Results:** From the 21 interviews with emergency and outpatient providers, this study used the CFIR construct of compatibility situated within the domain of the inner setting to identify four barriers to transitions of care for ED-initiated buprenorphine patients: scope of practice, prescribing capacity, referral incoordination, and loss to follow-up.

**Conclusion:** Next steps for implementation of this intervention in a community setting include: establishing a standard of care around treatment and referral for ED patients with OUD, increasing buprenorphine prescribing capacity, creating a central repository for streamlined referrals and follow-up, and supporting low barrier scheduling and navigation services.

## A15 “Evaluating the feasibility and acceptability of a computer assisted brief intervention for cannabis using court-involved non-incarcerated adolescents” (TR09)

### Nazaret C. Suazo, Lauren Micalizzi, Aya Cheaito, Kara Fox, Sara J. Becker, Kathleen Kemp, Anthony Spirito, and Lynn Hernandez

#### Lead Author Affiliation: Warren Alpert Medical School of Brown University, 222 Richmond St, Providence, RI 02903, USA

##### **Correspondence:** Nazaret C. Suazo (nazaret_suazo@brown.edu)

*Addict Sci Clin Pract* 2020, **15(Suppl 2)**:A15

**Background:** Little is known about improving the continuum of care for cannabis-using, court involved non-incarcerated (CINI) adolescents. Computer-assisted motivational interview (MI) interventions are inexpensive, with less demand on staff time and increased protocol fidelity, portability, and accessibility. This study examined the feasibility and acceptability of integrating a computer-assisted, brief MI into the intake procedures at the Rhode Island Family Court for cannabis-using adolescents and their parents.

**Methods:** 71 adolescents ages 14–18 years (Mage = 15.8; 73% male), who screened positive for cannabis use at intake, were recruited and randomized to one of two conditions: (1) a computer-assisted adolescent MI delivered by court staff plus an online parenting program (n = 34); or (2) psychoeducation (n = 37). Substance use outcomes were assessed at 3- and 6-month follow-ups. Feasibility outcomes include participant recruitment, retention, and independent ratings of court staff protocol adherence, use of MI principles and skills (e.g., rolling with resistance, supporting self-efficacy, ranging from 1[poor] to 5[excellent]) and therapeutic skills (e.g., feedback, understanding, ranging from 1[poor] to 6[excellent]). Teens reported on acceptability.

**Results:** Of 115 prospective participants, 71 were enrolled. Retention rates ranged from 78 to 86% across follow-ups. Court staff were 92.7% adherent to intervention protocols. Use of MI principles and skills was good (M = 3.47) and use of therapeutic skills was very good (M = 4.49). All acceptability ratings were high and positive. Adolescents indicated that the information was relevant (93%), they learned a lot (97%), could apply what they learned to their lives (98%), and would recommend the program to others (87%). Approximately 50% of adolescents liked or very much liked the content of the computer program and their conversation with staff.

**Conclusion:** Computer-assisted MIs for CINI adolescent cannabis use can be feasibly administered by court staff with high fidelity in court settings.

## A16 “The association between pill mill legislation and neonatal abstinence syndrome” (TR10)

### Jayani Jayawarhana, and Tahiya Anwar

#### Lead Author Affiliation: College of Pharmacy at University of Georgia, 250W Green Street, Athens, GA 30602, USA

##### **Correspondence:** Jayani Jayawarhana (jayaward@uga.edu)

*Addict Sci Clin Pract* 2020, **15(Suppl 2)**:A16

**Background:** Neonatal Abstinence Syndrome (NAS) is a drug withdrawal syndrome in newborns who were primarily been exposed to drugs such as opioids while in the womb. The rate of NAS in the U.S. has increased over the past decade. While use of opioids and opioid overdose deaths have increased over the years, many states have adopted various policies to combat the opioid epidemic. Pill mill legislation is one of these policies. Although, a few states have adopted pill mill legislation in the hopes of mitigating adverse effects of the opioid epidemic, its effect on NAS is not evident. This study examines the association between pill mill legislation and NAS rate.

**Methods:** The study utilizes state-level hospital discharge data from Healthcare Cost and Utilization Project’s (HCUP) database. HCUP database includes yearly data from 43 states from 2008 to 2017. Data from HCUP database were merged with state level socio-demographic data and opioid-related health policy data for the analysis. Analysis was carried out using a difference-in-differences regression approach.

**Results:** The regression results indicate that pill mill legislation is associated with 3.9 additional NAS cases per 1000 newborn hospitalizations and 6.4 additional NAS cases per 1000 newborn hospitalizations among Medicaid payers.

**Conclusions:** These findings indicate that pill mill legislation has not been effective in reducing NAS rates. This could be because states that have adopted pill mill legislation may be the states with higher rates of opioid utilization.

## A17 “Assessing perceived value of behavioral health workforce educational activities: question construction matters” (TR11)

### Susan A. Stoner, Denna Vandersloot, and Bryan Hartzler

#### Lead Author Affiliation: Alcohol & Drug Abuse Institute at University of Washington, 1107 NE 45th St, Seattle, WA 98105, USA

##### **Correspondence:** Susan A. Stoner (sastoner@uw.edu)

*Addict Sci Clin Pract* 2020, **15(Suppl 2)**:A17

**Background:** To examine how ratings of satisfaction, benefit, and usefulness of training and technical assistance activities provided to DHHS Region 10 behavioral health workforce members by the Northwest Addiction Technology Transfer Center varied by topic and format.

**Methods:** A standard post-event survey utilized by SAMHSA grantees including questions assessing satisfaction, benefit, and usefulness was administered after 192 events from 10/1/2017 through 8/31/2019. Five questions used 5-point Likert scales (strongly agree to strongly disagree or very satisfied to very dissatisfied). One used a 4-point scale (very useful to useless). Responses were recoded so that higher numbers indicated higher favorability, and the 4-point scale was recoded to a 5-point scale. Events were coded by topic (11 options) and format (training workshop, technical assistance, or webinar). Multivariate analyses of variance examined effects of topic and format on ratings.

**Results:** Across 3158 surveys, ratings were generally high (all 95% CIs > 4). Both topic and format significantly affected ratings, which were sensitive to question framing. For example, for “I expect this activity to benefit my clients,” motivational interviewing (MI) was rated significantly higher than 9 other topics (M = 4.64), but for “the material presented will be useful to me in dealing with substance abuse,” MI was not rated higher than any other (M = 4.31). For “I expect this to use the information gained from this activity,” webinars were rated significantly lower than other formats (M = 4.29), but for “the material presented will be useful to me in dealing with substance abuse,” webinars were not rated lower (M = 4.32).

**Conclusion:** Findings revealed that questions that might seem similar yielded different results and conclusions. Such variation lends support to survey revision in September 2019 that streamlined this post-event assessment task. These regional findings support those revisions due to the effects of question wording.

## A18 “Barriers and facilitators to drug use behavior change in diverse community health center patients in Los Angeles” (TR12)

### Stephanie Sumstine, Michael Park, Whitney Akabike, Dallas Swendeman, and Lillian Gelberg

#### Lead Author Affiliation: Department of Psychiatry and Biobehavioral Sciences at University of California, Los Angeles, 760 Westwood Plaza, Los Angeles, CA 90095, USA

##### **Correspondence:** Stephanie Sumstine (SSumstine@mednet.ucla.edu)

*Addict Sci Clin Pract* 2020, **15(Suppl 2)**:A18

**Background:** Self-monitoring and feedback are core elements for supporting behavior change over time. Feedback provided by a therapist or counselor has limited scalability beyond session-limited interventions. There is evidence that automated text-messaging may be efficacious for reducing substance use, however, several studies have not observed drug use reductions when feedback messages were completely automated, not personalized, and did not involve any human contact. Automated feedback messages that are tailored to individuals’ barriers and facilitators to drug use reduction may be a scalable, yet personalized, strategy to enhance counselor delivered interventions and sustain patient engagement in drug use reduction goals after counseling sessions cease. This analysis aimed to identify barriers and facilitators to drug use reduction to guide tailoring of personalized feedback text-messages in response to weekly self-monitoring by patients with moderate risk drug use in the new NIDA-funded QUIT-Mobile study.

**Methods:** Analysis included thematic content analysis of QUIT-Binational study health educator coaching log data. Two research assistants closely examined the data to identify common themes through iterative rounds of coding and discussion with the study team.

**Results:** The most common barriers to drug use reduction cited by QUIT-Binational participants were: (1) peers/social environment, (2) relaxation and being able to “mellow out”, (3) pain relief, and (4) perceived to work better than prescribed medication. The most common facilitators that helped participants stay focused on their drug use reduction goals were: (1) exercise, (2) family and peer support, (3) motivation in spending less money, (4) alternative pain relief (i.e. stretching), and (5) relaxation techniques (i.e. meditation, journaling).

**Conclusion:** Findings suggest there are unique barriers and facilitators to drug use reduction in diverse low-income primary care patients. Feedback messages should be tailored to individuals’ noted barriers and facilitators to enhance motivation and reinforce alternatives to drug use. Future studies should comprehensively examine how cultural, social, and environmental aspects influence drug use to develop specialized feedback that appeals to diverse patients to a greater degree than standard feedback.

## A19 “Understanding readiness to begin SU treatment among substance using justice-involved young adults” (TR13)

### Sin Lee, Taryn Sirias, Stephanie Campos, Megan O’Grady, Susan Tross, Patrick Wilson, Renee Cohall, Alwyn Cohall, and Katherine Elkington

#### Lead Author Affiliation: New York State Psychiatric Institute, 1051 Riverside Dr, New York, NY 10032, USA

##### **Correspondence:** Sin Lee (Sin.lee@nyspi.columbia.edu)

*Addict Sci Clin Pract* 2020, **15(Suppl 2)**:A19

**Background:** Justice-involved young adult (JIYA), ages 18–24, are at higher risk of substance use/disorder (SU/D) than their non-justice-involved counterparts. Nonetheless, uptake of SU treatment remains low, and our understanding of barriers to SU treatment uptake in JIYA remains incomplete. This study explores factors that affect SU treatment readiness in JIYA. By exploring these factors we can gain insight into the barriers and facilitators to uptake of SU treatment among JIYA.

**Methods:** We conducted interviews (n = 153) with JIYA [64.5% African American (AA); 37.5% Latinx; 72.9% male; mean age 20.7] recruited from an alternative to sentencing program. Interviews examined SU frequency, prior treatment history and current treatment needs (determined by DAST, AUDIT scales), readiness to change SU behaviors (0, not ready to 10, ready now) and readiness to begin treatment (0, not ready to 10, ready now). Linear regression models were fit to explore predictors of treatment readiness.

**Results:** Almost all (91.1%) JIYA self-reported in using substance in the past 12 months. 69.3% reported SU that indicated further assessment was necessary [49.3% reported DAST scores in harmful range (> 3) and 9.9% had reported AUDIT scores in the harmful range (> 15)]. However, only 21.7% reported to have prior experience with SU treatment/Counseling. Over 65% reported 6 or higher on the readiness to change SU behaviors scale; in contrast 60.5% reported a 0 on the readiness to begin treatment scale. Males and Latinx JIYA reported lower treatment readiness scores. While African American JIYA, and those with higher DAST and SU change scores, had higher treatment readiness scores.

**Conclusion:** Problematic SU is highly prevalent among JIYA yet uptake of treatment remains low. The inverse relationship between readiness to change and readiness to begin treatment among JIYA suggests low treatment uptake is likely not a function of low insight into their own SU. Linkage programs should address concerns and negative perceptions related to SU treatment, leveraging readiness to change as a point of departure. Linkage to treatment programs that particularly target males and Latinx JIYA are warranted.

## A20 “The cost of providing extended-release naltrexone treatment for opioid use disorder to persons who are incarcerated, prior to reentry” (TR14)

### Philip J. Jeng, Ali Jalali, Danielle A. Ryan, Sabrina A. Poole, Frank J. Vocci, Michael S. Gordon, George E. Woody, Daniel Polsky, and Sean M. Murphy

#### Lead Author Affiliation: Weill Cornell Medical College, 1300 York Ave, New York, NY 10065, USA

##### **Correspondence:** Philip J. Jeng (phj2003@med.cornell.edu)

*Addict Sci Clin Pract* 2020, **15(Suppl 2)**:A20

**Background:** Persons with opioid use disorder (OUD) who are incarcerated are highly susceptible to opioid-overdose upon reentry. If initiated prior to release, extended-release naltrexone (XR-NTX) provides ~ 30 days of opioid-overdose protection in the community. XR-NTX's high cost is perceived as a barrier. Estimate implementation and ongoing-management costs associated with different strategies of XR-NTX delivery to persons with OUD upon reentry.

**Methods:** Data were from two multisite randomized-controlled effectiveness trials comparing pre-release XR-NTX + referral to community pharmacotherapy to: referral only (Study A); pre-release XR-NTX + post-release place-of-residence/mobile treatment (Study B). A micro-costing approach was used. We solicited estimates of resources required to deliver each strategy. All intervention-relevant resources were included and valued. The resource-costing method was used, with unit costs derived from sources reflecting national “real-world” costs. Resources varied by study, and included: labor, medication, supplies, and provider travel (mileage, time). Costs were categorized as fixed, time-dependent, and variable. Year 1 costs included (a), (b), and (c) variable. Subsequent annual costs included (b) and (c).

**Results:** The in-prison XR-NTX process was estimated to take 2–3.5 h. Study A adopted an in-house model. Study B’s intervention was delivered by an outside team. Fixed/one-time costs and time-dependent costs were minimal; consequently, per-patient costs vary little with changes in patient caseload. Assuming full capacity, year 1, per-patient costs were estimated as 979 dollars (Study A), and 3458 dollars (Study B); subsequent annual costs were 976 dollars/patient and 3453 dollars/patient, respectively. 1320 dollars/patient in Study B was associated with travel to prison, and 1007 dollars/patient was associated with the post-release mobile portion.

**Conclusions:** Results are valuable to stakeholders interested in expanding XR-NTX OUD treatment in justice settings.

## A21 “Screening in trauma for opioid misuse prevention (STOMP): results from a prospective cohort of victims of traumatic injury” (TR15)

### Bri Deyo, Randall T. Brown, Christopher Nicholas, Amelia Baltes, Scott Hetzel, Alyssa Tilhou, Andrew Quanbeck, Joseph Glass, and Suresh Agarwal

#### Lead Author Affiliation: University of Wisconsin, Madison, 1100 Delaplaine Ct #1896, Madison, WI 53715, USA

##### **Correspondence:** Bri Deyo (Bri.deyo@fammed.wisc.edu)

*Addict Sci Clin Pract* 2020, **15(Suppl 2)**:A21

**Background:** Traumatic injury frequently requires opioid analgesia to manage pain and avoid catastrophic complications. Screening practices to minimize opioid misuse and de novo use disorder remains an area of much needed investigation. This study seeks to identify patient factors predictive of opioid misuse or use disorder after a traumatic injury.

**Methods:** Six-month prospective cohort study of 295 inpatients of Trauma and Orthopedic Surgical Services at a Level I trauma center. Data included validated survey tools and health record review at baseline (during hospitalization) and at 4, 12, and 24 weeks after discharge. Participants were English speaking, aged 18–75 years, with discharge opioid prescription for independent administration. Surveys included Post-Traumatic Stress Disorder Checklist-5 (PCL-5), Patient Health Questionnaire, Generalized Anxiety Disorder scale, Pain Catastrophizing Scale, and Opioid Risk Tool (ORT). Health record data included diagnosis codes, procedures, and pain severity. Primary outcomes were opioid use disorder (determined by Clinical International Diagnostic Interview-Substance Abuse Module) and/or opioid misuse (determined by survey) at 24 weeks post-discharge.

**Results:** Of 295 participants, 237 completed the 24-week assessment. Stepwise regression model building demonstrated pre-injury PTSD symptoms, Opioid Risk score, and length of stay predicted misuse and use disorder [opioid misuse at 6 months: PCL-5 [OR 1.06, 95% CI (1.02, 1.10)]; ORT [1.17 (1.04, 1.34)]; length of stay [4.32 (1.24, 17.1)]. Receiver operating curves for misuse (AUC 0.880) and use disorder (AUC 0.943) were highly favorable.

**Conclusions:** The pre-injury presence of PTSD-related symptoms, impaired pain coping, and hospitalization for greater than 6 days predicted opioid misuse and de novo opioid use disorder at 6 months after hospital discharge. Behavioral screening and management strategies for PTSD and other anxiety-related syndromes appear warranted for traumatic injury victims to optimize pain management and reduce opioid-related risks.

## A22 “The balanced opioid initiative: protocol for a clustered, sequential, multiple-assignment randomized trial to construct an adaptive implementation strategy to improve guideline-concordant opioid prescribing in primary care” (TR16)

### Andrew Quanbeck, Nicholas Schumacher, Brienna Deyo, Randall Brown, and Rose Hennessy-Garza

#### Lead Author Affiliation: University of Wisconsin, Madison, 800 University Bay Drive, Suite 210 Madison, WI 53705, USA

##### **Correspondence:** Andrew Quanbeck (arquanbe@wisc.edu)

*Addict Sci Clin Pract* 2020, **15(Suppl 2)**:A22

**Background:** Rates of opioid prescribing tripled in the USA between 1999 and 2015 and were associated with significant increases in opioid misuse and overdose death. Roughly half of all opioids are prescribed in primary care. Although clinical guidelines describe recommended opioid prescribing practices, implementing these guidelines in a way that balances safety and effectiveness vs. risk remains a challenge. The literature offers little help about which implementation strategies work best in different clinical settings or how strategies could be tailored to optimize their effectiveness in different contexts. Systems consultation consists of (1) educational/engagement meetings with feedback reports (EM/AF), (2) practice facilitation (PF), and (3) prescriber peer consulting (PPC). This NIH-funded (R01DA047279) study is designed to discover the most cost-effective sequence and combination of strategies for improving opioid prescribing practices in primary care clinics.

**Methods:** The study is a hybrid type 3 clustered, sequential, multiple-assignment randomized trial that randomizes 40 clinics from two health systems at months 3 and 9, of a 21-month intervention. Clinics are provided one of four sequences of implementation strategies: a condition consisting of EM/AF, EM/AF plus PF, EM/AF + PPC, and EM/AF + PF + PPC.

**Results:** The primary outcome is morphine-milligram equivalent (MME) dose by prescribing clinicians within clinics. The primary aim is the comparison of EM/AF + PF + PPC versus EM/AF on change in MME from month 3 to month 21. The secondary aim is to derive and compare cost estimates for each of the four sequences.

**Conclusion:** Systems consultation is a practical blend of implementation strategies used in this case to improve opioid prescribing practices in primary care. The blend offers a range of strategies in sequences from minimally to substantially intensive.

The results of this study will help understand how to cost effectively improve the implementation of evidence-based practices.

## A23 “Development and evaluation of a technology-assisted intervention for parents of adolescents in residential substance use treatment” (TR17)

### Sara J. Becker, Sarah A. Helseth, Katherine I. Escobar, Timothy Janssen, and Anthony Spirito

#### Lead Author Affiliation: Brown University School of Public Health, 121 S Main St, Providence, RI 02903, USA

##### **Correspondence:** Sara J. Becker (sara_becker@brown.edu)

*Addict Sci Clin Pract* 2020, **15(Suppl 2)**:A23

**Background:** Approximately 60% of adolescents in residential substance use (SU) treatment relapse within 90 days of discharge. Parenting skills predict adolescent SU outcomes and likelihood of relapse, but engaging parents in treatment is challenging. Accordingly, there is a clear need for effective and scalable interventions for parents of adolescents in residential SU treatment. This pilot trial evaluated the feasibility, acceptability, and effectiveness of a technology-assisted parenting intervention called Parent SMART, as an adjunct to residential treatment as usual (TAU).

**Methods:** Parent SMART augments an off-the-shelf, research-tested, online parenting program (Parenting Wisely) with two scalable components: (1) up to four telehealth sessions, and (2) a mobile networking app, where parents can submit questions to an SU expert or connect with other parents of adolescents in residential treatment. We randomized 61 adolescent-parent dyads from two residential SU treatment programs to either TAU (n = 31) or Parent SMART + TAU (n = 30). Assessments at baseline, 6-, 12-, and 24-weeks post-discharge examined parenting skills, adolescent days of SU, and adolescent problems.

**Results:** Feasibility and acceptability targets were met or exceeded: 86% of parents completed at least 2 telehealth sessions and 2 online modules, 70% posted in the networking app, and 85–90% were retained at follow-up. Parents were significantly more satisfied with and likely to recommend Parent SMART to a friend than TAU. Mixed effect models revealed that Parent SMART was significantly more effective over time in increasing parental monitoring and communication, in reducing days of drinking, and in reducing school-related problems among parents of adolescents in the short-term residential program.

**Conclusion:** Results provide evidence of feasibility, acceptability, and preliminary effectiveness of Parent SMART as an adjunct intervention to improve outcomes among high-risk adolescents at a vulnerable time in their recovery process.

## A24 “Variation in substance use screening outcomes with commonly used screening strategies in primary care: findings from a multi-site implementation study of electronic health record-integrated screening for alcohol and drug use” (TR18)

### Jennifer McNeely, Angeline Adam, Leah Hamilton, Joseph L. Kannry, Richard N. Rosenthal, Sarah E. Wakeman, Timothy E. Wilens, Sarah Farkas, Aimee Wahle, Seth Pitts, Carmen Rosa, and John Rotrosen

#### Lead Author Affiliation: New York University Grossman School of Medicine, 550 1st Avenue, New York, NY 10016, USA

##### **Correspondence:** Jennifer McNeely (jennifer.mcneely@nyulangone.org)

*Addict Sci Clin Pract* 2020, **15(Suppl 2)**:A24

**Background:** Screening for alcohol and drug use is recommended for adult primary care patients, but primary care clinics frequently struggle to choose the approach that is best suited to their resources, workflows, and patient populations. To inform these decisions, we conducted a multi-site study to inform the implementation and feasibility of electronic health record (EHR)-integrated screening.

**Methods:** In two urban academic health systems, researchers worked with stakeholders from six clinics to define and implement their optimal screening approach. All clinics used single-item screening questions for alcohol/drugs followed by the AUDIT-C/DAST-10 for patients screening positive. Clinics chose between screening at routine vs. annual visits; and staff-administered vs. electronic self-administered screening. Results were recorded in the EHR, and data was extracted quarterly to describe implementation outcomes. Findings are from the first year after implementation.

**Results:** Across all clinics, among 93,114 patients with primary care visits, 72% were screened for alcohol and 71% were screened for drugs. Screening at routine encounters, in comparison to annual visits, achieved higher screening rates for alcohol (90–95% vs. 24–72%) and drugs (90–94% vs. 25–70%). Clinics using staff-administered screening, in comparison to patient self-administered screening, had lower rates of detection of unhealthy alcohol use (1.6% vs. 14.7–36.6%). Detection of unhealthy drug use was low at all clinics, ranging from 0.5 to 1.0%.

**Conclusion:** EHR-integrated screening was feasible to implement in all six clinics, though one had persistently lower screening rates than the others. Screening at routine primary care visits with a self-administered approach offered the most opportunities for identifying unhealthy alcohol use. Detection of drug use was low regardless of screening approach. Although limited by differences among clinics, this study provides insight into outcomes that may be expected with commonly used screening strategies in primary care.

## A25 “Co-occurring disorders of Medicaid/Medicare beneficiaries receiving methadone treatment in a small urban setting surrounded by rural communities” (HD01)

### Jamey J. Lister, Jennifer D. Ellis, Stella M. Resko, Amanda M. Stylianou, and Elizabeth Aguis

#### Lead Author Affiliation: Rutgers School of Social Work, 536 George St, New Brunswick, NJ 08901, USA

##### **Correspondence:** Jamey J. Lister (jlister@ssw.rutgers.edu)

*Addict Sci Clin Pract* 2020, **15(Suppl 2)**:A25

**Background:** Patients in methadone maintenance treatment (MMT) demonstrate high rates of co-occurring disorders (CODs). However, limited information exists about CODs in increasingly rural settings, particularly among Medicaid/Medicare beneficiaries. Our objectives were to identify rates and correlates of CODs, and differences between patients with and without past-year (PY) opioid misuse.

Methods. Medicaid/Medicare beneficiaries (N = 219) with opioid use disorder (OUD) (female = 61.9%, Non-Hispanic White = 85.8%; PY opioid misuse = 48.4%) completed cross-sectional surveys at an opioid treatment program providing MMT in a small urban setting surrounded by rural communities. Measures included sociodemographic and opioid characteristics, and screens for co-occurring emotional (current depression, anxiety, PTSD; PHQ-4, PC-PTSD-5) and substance use disorders (SUDs) (PY alcohol, cannabis, stimulant, sedative use disorders; AUDIT-C, SDS).

**Results:** At least one positive COD screen was observed in 78.1% of patients, with rates as follows: anxiety (48.4%), PTSD (44.0%), depression (41.2%), and disordered use of stimulants (30.1%), cannabis (19.2%), alcohol (16.1%), and sedatives (7.8%). Within OUD patients reporting PY opioid misuse, 89.6% screened positive for CODs, and the same group demonstrated higher rates for six CODs (stimulant: X2 = 34.93, P < 0.001; PTSD: X2 = 13.22, P < 0.001; sedative: X2 = 11.71, P < 0.001; anxiety: X2 = 8.47, P = 0.004; cannabis: X2 = 6.94, P = 0.008; alcohol: X2 = 4.75, P = 0.029). Across the sample, co-occurring sedative use disorder was more common among patients with less than a high school degree (X2 = 5.53, P = 0.019), overdose histories (X2 = 5.18, P = 0.023), and self-reported fentanyl use (X2 = 5.25, P = 0.034). Patients reporting fentanyl use demonstrated a higher rate of positive screens for PTSD (X2 = 8.39, P = 0.004) and stimulant use disorder (X2 = 5.18, P = 0.023). Race and gender did not differentiate CODs.

**Conclusion:** This analysis identifies high COD rates among a population underrepresented in the MMT literature. A single opioid misuse item was a key distinguisher of CODs. Integrated approaches are needed to address the COD burden in this underserved population.

## A26 “Defining patient-centered successful methadone treatment outcomes among low-income, minority individuals at a community-based outpatient treatment center” (HD02)

### Valerie Bradley, Mary Kleinman, Aaron Greenblatt, Annabelle Belcher, CJ Seitz-Brown, Hannah Tralka, Morgan Anvari, Thomas Cole, and Jessica Magidson

#### Lead author affiliation: University of Maryland, College Park College Park, MD 20742, USA

##### **Correspondence:** Valerie Bradley (vbradley@umd.edu)

*Addict Sci Clin Pract* 2020, **15(Suppl 2)**:A26

**Background:** In 2018, nearly 70% of drug overdose fatalities were attributable to opioids, disproportionately impacting low-income, ethnoracial minority individuals. Medication for opioid use disorder (MOUD) has established efficacy, yet less than 50% of patients are retained in care at 6 months with research pointing to the importance of 6-month retention for long-term treatment outcomes. While MOUD success is often defined through abstinence and relapse, a more comprehensive understanding of successful outcomes may help inform efforts to increase treatment success and improve quality of life. This study seeks to define patient-centered successful MOUD outcomes through qualitative work at an outpatient methadone program serving a predominantly low-income, ethnoracial minority population in Baltimore City.

**Methods:** Semi-structured interviews (n = 9) and focus groups (n = 23) were conducted with patients, staff, and peer recovery coaches (PRCs) at an opioid treatment program. Patients were asked what doing well in the methadone program looked/felt like. Staff and PRCs were asked to define treatment success based on their clients’ experiences. Interviews and focus groups were transcribed and thematically coded to consensus by two trained coders.

**Results:** Patients (n = 20) had a mean age of 48.4 (SD = 10.0), were 70% male, and 60% Black or African American. Staff and PRCs (n = 12) had a mean age of 49.2 (SD = 0.7), were 42% male, and 75% Black or African American. Participants identified patient-centered successful treatment outcomes as improved health (mental/physical), stability and productivity (stable housing, employment), social behaviors (mending/building relationships), improved sense of self-worth (pride, valuing life), absence of substance-driven behaviors (saving paychecks, avoiding negative influences), MOUD engagement (maintaining stable dose, planned/tapered methadone discontinuation), and reduction in or abstinence from substance use.

**Conclusion:** Understanding patient-centered definitions of successful MOUD outcomes may help inform interventions aiming to improve treatment retention and success. Our findings will inform how treatment success is defined and evaluated in a subsequent clinical trial at this site. Keywords (2–5): medication treatment for opioid use disorder, retention, treatment outcomes.

## A27 “Differences in recovery from alcohol and drug problems in the United States population based on LGBTQ+ status: prevalence, pathways, and psychological well-being” (HD03)

### Amanda K. Haik, M. Claire Greene, Brandon G. Bergman, and John F. Kelly

#### Lead author affiliation: Massachusetts General Hospital, 151 Merrimac St, Boston, MA 02114, USA

##### **Correspondence:** Amanda K. Haik (ahaik1@mgh.harvard.edu)

*Addict Sci Clin Pract* 2020, **15(Suppl 2)**:A27

**Background:** Alcohol and other drug (AOD) use disorders are a significant public health concern and LGBTQ+ individuals are overrepresented among this population likely due to substantial biobehavioral stress from stigmatization. Little is known about the characteristics of this population and differences between LGBTQ+ and heterosexual individuals on clinical and service use histories and current well-being in recovery.

**Methods:** Data are from the National Recovery Study—a nationally representative sample of US adults (18+) who have resolved an AOD problem (N = 2002). Chi-square or ANOVA tests tested for differences in socio-demographics and AOD use/treatment and psychiatric/legal factors. Unadjusted linear regression analyses tested for group differences on indices of current well-being (e.g., psychiatric distress, quality of life, happiness, self-esteem). LOWESS graphs were computed to show differences between groups across time on well-being indices. Linear regression models factored in variables that were significantly different between groups in univariate analyses in order to investigate which variables might account for observed differences on indices of well-being.

**Results:** 11.7% identified as LGBTQ+. Compared to heterosexual individuals (n = 1666), LGBTQ+ (n = 220) were less likely to be employed (OR = 0.64; 95% CI 0.43, 0.96) and had significantly fewer years since problem resolution (OR = 0.97; 95% CI 0.96, 0.99). LGBTQ+ also evidenced more markers of severity, including being 2.2 times as likely to have a co-occurring psychiatric disorder (95% CI 1.49, 3.37). Unadjusted models showed that LGBTQ+ had significantly worse levels on all well-being indices. Models factoring in significantly different socio-demographic, AOD use/treatment, and psychiatric/legal factors explained most of these differences, except for psychological distress.

**Conclusion:** LGBTQ+ individuals evinced more problematic psychiatric and legal histories and faced greater psychosocial challenges in recovery. Further research is needed to better understand the unique experiences of recovering LGBTQ+ individuals in order to address observed disparities in well-being.

## A28 “Growth in recovery among emerging adults from ethnic minority backgrounds: impact of social support and life events” (HD04)

### Craig Henderson, Tessa Long, Lauren Ryan, Temilola Salami, Amanda Venta, and Elise Yenne

#### Lead author affiliation: Sam Houston State University, 1905 University Ave, Huntsville, TX 77340, USA

##### **Correspondence:** Craig Henderson (chenderson@shsu.edu)

*Addict Sci Clin Pract* 2020, **15(Suppl 2)**:A28

**Background:** Substance use peaks during emerging adulthood and leads to negative outcomes well into the adult years. The negative consequences of substance use are particularly noteworthy in ethnic minority groups. Discrimination has been highlighted in previous research with ethnic minority populations as a salient factor underlying escalating substance use, but it has not been studied in clinical samples of emerging adults. Emerging adulthood has been highlighted as a sensitive period for increased exposure to discriminatory acts for ethnic minority youth, and the entrenchment of substance use behaviors across ethnicity. We examined the following hypotheses: (1) perceived discrimination will be associated with longitudinal trajectories of substance use such that higher levels of discrimination are associated with increasing levels of substance use; (2) time-varying associations between discrimination and substance use trajectories will be moderated by assumption of adult roles; (3) time-varying associations between discrimination and substance use trajectories will be moderated by social support.

**Methods:** Hypotheses were evaluated using the current study used data from the Global Appraisal of Individual Needs dataset. Propensity score matching was first use to balance participants who reported perceiving that they had been discriminated against and those who had not on a number of background variables (final n = 386). Latent growth curve modeling was used to test study hypotheses.

**Results:** contrary to expectations, participants reporting discrimination were more likely to enter recovery over the 12 month follow-up period. However, when social support and emerging adult life events were taken into account the effect was smaller. Results of the final model indicated that early, consistent employment was most influential in promoting participant recovery regardless of whether or not participants identifying as ethnic minority perceived they had been discriminated against.

**Conclusion:** Securing employment during substance use treatment helps offset the negative impact of discrimination among emerging adults.

## A29 “Identifying disparities in unmet behavioral health need and treatment initiation among youth on probation” (HD05)

### Margaret Ryan, Katherine Elkington, and Gail Wasserman

#### Lead author affiliation: New York State Psychiatric Institute, 1051 Riverside Dr, New York, NY 10032, USA

##### **Correspondence:** Margaret Ryan (Margaret.Ryan@nyspi.columbia.edu)

*Addict Sci Clin Pract* 2020, **15(Suppl 2)**:A29

**Background:** Youth on probation have a high burden of behavioral health (BH) treatment need, including mental health (MH) and substance use (SU). The pathway to treatment includes screening for BH need, referral, and initiation. Moreover, these pathways vary by need type, yet whether these differences are associated with disparities in need, referral and initiation is unknown. We aimed to examine this association and to identify whether BH need type (MH, SU or both) might influence these outcomes among juvenile probationers.

**Methods:** Administrative data on BH screening [Youth Assessment and Screening Instrument (YASI)], referral, and initiation were collected from 10 NYS probation departments (08/01/2019 through 05/31/2020; N = 697 Juvenile Delinquent intakes). We examined need type (MH Only, SU Only, or Both MH and SU), controlling for age, gender, and race along each step in a series of multivariate logistic regression analyses.

**Results:** Proportion of unmet need varied by identified need type: 67% of MH (126 of 187), 90% of SU (45 of 50), and 80% of both (78 of 98), as did treatment initiation among those with unmet need: 13% of MH (16 of 126), 7% of SU (3 of 45), and 15% of both (12 of 78). Controlling for demographics, youth with SU need were three times more likely to have untreated need (OR = 3.22 [95% CI 1.19, 8.75], p = 0.022), compared with those with MH need. **Conclusion:** Findings point to disparities between the amount of probationers identified with unmet SU and MH treatment need and linked with treatment. Factors that likely contribute to these disparities, including low availability of adolescent SU services and lack of collaboration between probation and treatment, are discussed.

## A30 “Examining existing barriers to public health interventions and medical services for rural people who use drugs in the COVID-19 era” (HD06)

### Brent Van Ham, Kris Rosentel, Rebecca S. Bolinski, Suzan Walters, Jerel M. Ezell, John Bresett, and Mai T. Pho

#### Lead author affiliation: Southern Illinois University School of Medicine, 801N Rutledge St, Springfield, IL 62702, USA

##### **Correspondence:** Brent Van Ham (bvanham49@siumed.edu)

*Addict Sci Clin Pract* 2020, **15(Suppl 2)**:A30

**Background:** The COVID-19 pandemic has led to unprecedented public health measures such as social distancing and changes to services including restricting face-to-face encounters and the acceleration of telemedicine. People who use drugs in rural areas may be increasingly vulnerable in this setting.

**Objectives:** We explore potential predisposing factors impacting access to information and care for rural people who inject drugs (PWID) and/or people who used opioids (PWUO) non-medically in the pre-COVID era.

**Methods:** We surveyed rural PWID/PWUO before the pandemic regarding social determinants, drug use, barriers to medical and substance use treatment, and technology use and generated descriptive statistics using R Suite.(tm).

**Results:** Between July 2018 and July 2019, 173 current PWID/PWUO were surveyed (58% male; 86% White, 10% Black, 1.7% American Indian; mean age 40 years). Methamphetamines were the most frequently used drug (88% in the past 30 days) followed by opioid painkillers (73%), and benzodiazepines (70%). 49% had been homeless in the past 6 months. 21% were uninsured while 66% had Medicaid or Medicaid expansion. Only 49% had transportation to appointments, 26% could not pay for care, 20% did not trust doctors, and 44% feared they would be treated with disrespect. Over half felt uncertain or disagreed that they would hear about an infection spreading amongst PWID. Word of mouth was the most common way respondents thought they would hear about an infection (66%), followed by television (21%) and social media (12%). 33% reported accessing the internet less than daily and 31% did not have active cell phone service.

**Conclusion:** Existing barriers such as homelessness, poverty, and distrust of providers may limit measures such as quarantine and self-isolation. Telemedicine may mitigate transportation barriers, however this may be undermined by lack of phone service, costs, and underlying stigma faced by PWID/PWUO. Peer interventions may leverage existing networks to disseminate information.

## A31 “Examining the impact of jail sanctions on drug court program completion” (HD07)

### Lisa Shannon, Afton Jones, Jennifer Newell, and Elizabeth Nichols

#### Lead author affiliation: Morehead State University, 150 University Blvd, Morehead, KY 40351, USA

##### **Correspondence:** Lisa Shannon (l.shannon@moreheadstate.edu)

*Addict Sci Clin Pract* 2020, **15(Suppl 2)**:A31

**Background:** Drug court is a community-based rehabilitation program for individuals with substance use issues and criminal justice involvement. Extant drug court research suggests effectiveness via reduced recidivism and other positive outcomes, particularly for graduates. Emerging research has shown the impact of sanctions/therapeutic responses on program completion. Data from Wu and colleagues (2012) suggested program graduates were less likely to receive jail sanctions in comparison to program terminators. Another study showed the timing of the first sanction is also highly predictive of program retention (Brown, Allison & Nieto 2010). The current study’s purpose was to further examine the impact of jail sanctions on drug court program completion.

**Methods:** Fourteen Kentucky Drug Court (KDC) sites were sampled to represent each of the service regions statewide. A random sampling plan identified program participants (between February 16, 2008 to December 13, 2013) for inclusion (n = 50) from each selected site. Data from the assessment at program entry, Management Information System, and criminal justice involvement were examined.

**Results:** Bivariate analyses examined between-group differences based on completion status (graduates: n = 286; terminators: n = 414). Multivariate analysis utilizing Cox regression examined variables associated with program completion status accounting for the passage of time. Participants who had a jail sanction within the first 30 days of drug court participation had an increased hazard of program termination.

**Conclusion:** While responses/sanctions (including incarceration) are a component cited in the Defining Drug Courts: The Key Components, findings indicate incarceration, particularly within the first 30 days, may hinder an individual’s rehabilitative progress by increasing the hazard of program termination. These findings underscore that incarceration as a sanction be utilized sparingly; the Best Practice Standards implores the use of incarceration when the individual is an immediate public safety risk or after other consequences have been ineffective.

## A32 “Impact of Medicaid supportive housing health home program on health care utilization for people living with HIV/AIDS” (HD08)

### Sarah Forthal, Sugy Choi, Rajeev Yerneni, and Charles Neighbors

#### Lead author affiliation: Center on Addiction, 485 Lexington Ave, New York, NY 10017, USA

##### **Correspondence:** Sarah Forthal (sforthal@centeronaddiction.org)

*Addict Sci Clin Pract* 2020, **15(Suppl 2)**:A32

**Background:** Unstable housing among people living with HIV/AIDS (PLWHA) has been consistently linked to poor HIV-related care engagement. Chronic comorbidities such as substance use disorders (SUDs) are common in this population and may further complicate treatment engagement, leading to poor clinical outcomes. Between 2012 and 2018, the New York State (NYS) Department of Health implemented a pilot supportive housing program for eligible clients in Medicaid Health Homes (HH), a comprehensive care management program for individuals with chronic conditions. The Health Homes Supportive Housing Pilot (HHSP) provided long-term supportive housing services to HH-enrolled, chronically homeless PLWHA. We assessed the impact of HHSP on health care utilization (outpatient services, emergency department (ED) visits, and hospitalizations).

**Methods:** We analyzed monthly longitudinal data consisting of linked HHSP data and administrative data from NYS (excluding New York City) between 2012 and 2017. We used time series analysis to examine health care utilization as a function of HHSP enrollment, controlling for demographic variables and comorbid diagnoses. HHSP by SUD interactions were assessed using the cross-product term.

**Results:** The final sample included 250 HHSP-enrolled PLWHA. The most common comorbid diagnosis was SUD (57.2%). Those with at least 6 consecutive months of HHSP had 20% higher odds of using an outpatient service, 19% lower odds of visiting the ED, and 24% lower odds of being hospitalized compared to those with less. The outpatient interaction between HHSP and SUD was positive and significant (p = 0.012).

**Conclusion:** HHSP was effective in decreasing the likelihood of ED visits and hospitalizations while increasing the likelihood of outpatient visits for a group of unstably housed PLWHA. The increase in outpatient visits was larger among those with SUD. These findings suggest that supportive housing may promote better medical management by increasing outpatient visits among chronically homeless PLWHA with SUD.

## A33 “Implementing a peer recovery coach-delivered behavioral intervention to support engagement in substance use treatment from a community setting in Baltimore city” (HD09)

### Mary Kleinman, Jessica F. Magidson, Kelly Doran, Christopher J. Seitz-Brown, Emily N. Satinsky, Frances Loeb, Valerie D. Bradley, Dwayne Dean, and Julia Felton

#### Lead author affiliation: University of Maryland, College Park College Park, MD 20742, USA

##### **Correspondence:** Mary Kleinman (mkleinm@umd.edu)

*Addict Sci Clin Pract* 2020, **15(Suppl 2)**:A33

**Background:** Low-income, ethnoracial minorities disproportionately experience poor substance use (SU) treatment outcomes and need support to increase engagement and retention in treatment. Peer recovery coaches (PRCs), individuals with lived experience of SU and recovery, can reach individuals from community settings to support engagement in care. The aim of this study was to determine the feasibility, acceptability, and preliminary efficacy of a PRC-delivered behavioral intervention to reduce problematic SU and support engagement in SU treatment from a community-based setting.

**Methods:** This study took place at a community resource center serving unstably housed and low-income individuals. We piloted a PRC-delivered weekly, eight-session behavioral activation (BA) intervention designed to increase engagement in SU treatment by increasing substance-free positive reinforcement. We recruited adults with moderate- to high-risk problematic SU interested in harm reduction or SU treatment and not currently enrolled in SU treatment. A structured feasibility/acceptability assessment was administered at the end of study participation.

**Results:** The PRC linked eight guests to SU treatment over 6 months based on individual preferences and needs (e.g. inpatient or outpatient program; medication for opioid use disorder). Current SU included: opioids (n = 5), crack/cocaine (n = 5), alcohol (n = 6), cannabis (n = 3), and sedatives (n = 1). Seventy-five percent of participants reported polysubstance use. Two participants were lost to follow-up before initiating BA sessions. Four participants completed all eight BA sessions, one participant discontinued after four sessions, and one participant discontinued after three sessions. All participants (n = 6) reported decreases in SU and five participants remained in treatment at the time they ended the intervention. Overall intervention acceptability and perceived feasibility were high.

**Conclusions:** Findings suggest a PRC-delivered intervention in a community setting is feasible to engage individuals not otherwise connected to clinical care and acceptable to participants. Future research should examine the efficacy of PRC-delivered BA to reduce SU and support retention in treatment.

## A34 “Outcomes for clients experiencing homeless & trauma in a Hispanic Massachusetts addiction treatment agency providing integrated primary and mental health care” (HD10)

### Nick Huntington, Mary Jo Larson, Diliana De Jesús, Cynthia A. Tschampl, Yinuo Xu, Melisa Canuto, Melinda D'Ippolito, Micaurys Guzman, Emily Stewart, and Lena Lundgren

#### Lead author affiliation: Heller School for Social Policy and Management at Brandeis University, 415 South St, Waltham, MA 02453, USA

##### **Correspondence:** Nick Huntington (nhuntington@brandeis.edu)

*Addict Sci Clin Pract* 2020, **15(Suppl 2)**:A34

**Background:** Addiction treatment organizations that integrate medical/behavioral healthcare at the same geographic location may reach a broader group of patients than traditional programs and deliver integrated care to a high-need, high-risk population. This study examined outcomes for clients experiencing combinations of homelessness and traumatic experience in one of the largest integrated care addiction treatment providers in Massachusetts, Casa Esperanza, Inc.

**Methods:** Interviews were conducted with participants in Casa Esperanza’s Comprehensive Integrated Treatment Approach (CITA) project (SM060845-0) at intake and 6-months. The analysis here is based on 199 participants (63%) with data at both time points. We examined a set of six dichotomous indicators indexing different aspects of health and well-being (employment, illegal drug use, anxiety, depression, health, and pain) and formed a summary index score by summing the six indicators at each timepoint. To examine change by homelessness and traumatic experience we formed a categorical variable with three levels: (1) not homeless and did not report traumatic events that resulted in their feeling emotionally or physically harmed or threatened, (2) either homeless or reported traumatic experiences, both homeless and reported traumatic experiences. We regressed follow-up index score at follow-up on the baseline value, the homeless/trauma measure, and demographic covariates.

**Results:** The mean number of domains positively endorsed increased in all three groups between baseline and follow-up, indicating positive change over time. The group that was homeless and had experienced traumatic events started lower and improved less over time, controlling for baseline starting place.

**Conclusion:** Homelessness and traumatic experience are important factors in shaping how patients interact with, and benefit from, addiction treatment programs.

## A35 “Latino subgroup disparities in completion of addiction treatment: advancing disparities research” (HD11)

### Tenie Khachikian, Yinfei Kong, Daniel L. Howard, Bryan R. Garner, Benjamin Cook, Luis Torres, Richard Cervantes, and Erick G. Guerrero

#### Lead author affiliation: University of Chicago, 5801 S Ellis Ave, Chicago, IL 60637, USA

##### **Correspondence:** Tenie Khachikian (tenie@uchicago.edu)

*Addict Sci Clin Pract* 2020, **15(Suppl 2)**:A35

**Background:** The present study examines disparities in Latino subgroups’ completion of substance use disorder (SUD) treatment. Latinos represent the largest minority group entering SUD treatment in the United States, yet there is scant research specific to their performance in SUD treatment programs. Since Latinos are the most likely uninsured minority group, we tested the role of programs’ acceptance of Medicaid payments in reducing disparities.

**Methods:** We analyzed client and program data from 122 publicly funded SUD treatment programs across Los Angeles County in 2010, 112 programs in 2013, and 105 programs in 2015. These data were merged with information regarding 38,171 adult clients from all three time periods, of whom we selected Mexican Americans (32%), Cubans (0.3%), Puerto Ricans (0.6%), other Latinos (8.6%), and non-Latino Whites (58%). Multilevel logistic regression was used to examine program and client level factors associated with Latino subgroups and White differences in treatment completion.

**Results:** Mexican Americans had the lowest Medicaid coverage, attended programs of lower quality, while reporting lower levels of mental illness. Mexicans were the only Latino subgroup that reported disparities in treatment completion.

**Conclusions:** Implications of study findings can inform future research focused on differences among Latino subgroups to identify vulnerabilities and strengths of each group in positively responding to SUD treatment.

## A36 “Marijuana and E-cigarette use among Latinx and Hispanic population” (HD12)

### Aradhana Srinagesh, Michelle Loraly Martinez, Shankar Ramkellawan, Elizabeth Mustacchio, and Rachel Chernick

#### Lead author affiliation: Center on Addiction, 485 Lexington Ave, New York, NY 10017, USA

##### **Correspondence:** Aradhana Srinagesh (asrinagesh@centeronaddiction.org)

*Addict Sci Clin Pract* 2020, **15(Suppl 2)**:A36

**Background:** There are growing concerns over the increasing rates of electronic cigarette (e-cigarette) and marijuana use. It is especially important to investigate the impact on vulnerable populations including youth, of whom currently report the highest use rates (McMillen et al. 2015). Furthermore, it is crucial to address e-cigarette and marijuana use among ethnic minorities, who have been historically disproportionately affected. Individuals who identify as Latinx and/or Hispanic are among the most rapidly growing ethnic group in the United States, so focusing on this group is particularly important. Saddleson and colleagues (2015) report e-cigarette use among Hispanic adolescents and young adults has increased in recent years. And, among some samples has surpassed the rate among Caucasians (Bostean et al. 2015; Leventhal et al. 2015). The national Youth Risk Behavior Surveillance System survey reports 42.1% of Hispanic youth have used marijuana at least once, and 24.4% reported using marijuana within the month before completing the survey (King et al. 2015).

Methods. We compared use trends reported by our Spanish and English callers (parents or loved one) during a phone call with our Helpline Specialists.

**Results:** For both English and Spanish callers, a child/loved one struggling with ongoing substance use was reported as the most common concern. In addition, a caller's child/loved one’s age fell within the range of 12–28 years.

**Conclusion:** As the rate of Latinx/Hispanic youth continues to grow in the United States, understanding factors that impact substance use among these youth is imperative. Prevention and treatment efforts should incorporate the diverse backgrounds and experiences of this ethnic minority group.

## A37 “Measuring spatial inequities in access to opioid use disorder treatment for reducing HCV transmissions” (HD13)

### Jonathan Ozik, Marynia Kolak, Qinyun Lin, Harel Dahari, Basmattee Boodram, John Schneider, Eric Tatara, and Nicholson Collier

#### Lead author affiliation: Argonne National Laboratory, 9700 S Cass Ave, Lemont, IL 60439, USA

##### **Correspondence:** Jonathan Ozik (jozik@anl.gov)

*Addict Sci Clin Pract* 2020, **15(Suppl 2)**:A37

**Background:** Access to medication for opioid use disorder (MOUD) is essential for improving health outcomes and reducing HIV and hepatitis C virus infections. This study aims to demonstrate how varying geographic distributions of MOUD resources, reflecting different dimensions of equity, may impact health outcomes among people who inject drugs (PWID).

**Methods:** We evaluated three MOUD interventions (Methadone, Naltrexone, Buprenorphine) using different scenarios of varying levels of social and spatial health inequity in HepCEP, a validated agent-based model for Hepatitis C Elimination among the PWID population in Chicago and surrounding suburban areas. Specifically, we used the existing data from the synthetic population and infrastructure within the HepCEP model to newly apply MOUD interventions to model behaviors of PWID agents. To approximate potential access to resources, we calculated distance to the nearest MOUD provider using 2019 locations from the Substance Abuse and Mental Health Services Administration. We developed several hypothetical distributions of physical MOUD locations to reflect spatially random (i.e. equally distributed) and need-based patterns, and explored how different distributions of MOUD influences the MOUD intervention effects.

**Results:** The MOUD interventions are designed to reduce or stop the frequency of injection activities, thereby disrupting the PWID networks within HepCEP, which in turn reduces hepatitis C transmission. However, our results showed that different spatial distributions of MOUD resource locations can lead to substantially different MOUD intervention effects on the behaviors of PWID and their downstream health outcomes. For example, with MOUD locations equally distributed through the area, the average treatment duration for Methadone would increase in 60% of zip code areas, and the average new chronic infection rate would decrease by 33% of areas by 2030.

**Conclusion:** A spatial perspective is essential to understand the MOUD treatment heterogeneity that reflects the complex factors underlying social and spatial health inequity.

## A38 “Medicaid expansion reduces disparities in treatment access and retention in addiction health services” (HD14)

### Erick Guerrero, Tenie Khachikian, Yinfei Kong, Avelardo Valdez, Christine Grella, and Thomas D'Aunno

#### Lead author affiliation: I-Lead Institute, 12300 Wilshire Blvd Suite #210, Los Angeles, CA 90025, USA

##### **Correspondence:** Erick Guerrero (erickguerrero454@gmail.com)

*Addict Sci Clin Pract* 2020, **15(Suppl 2)**:A38

**Background:** The Medicaid expansion has provided an opportunity to eliminate racial and ethnic disparities in treatment access and engagement in addiction health services (AHS) in the United States. By implementing public insurance coverage and delivering coordinated mental health and HIV testing, high capacity treatment programs may eliminate disparities in wait time and retention. High capacity programs in this study accept Medicaid payments and have high levels of director leadership and staff readiness for change. In this IRB approved study, we examined the extent to which program capacity leads to higher implementation of coordinated care and in turn increased treatment access and engagement among Latinos and African Americans comparing pre- and post-Medicaid expansion.

**Methods:** We analyzed publicly available multi-year data from clients and programs at four points. We analyzed two waves during pre-expansion in 2011 (N = 115 programs, n = 11,526 clients) and 2013 (N = 111 programs, n = 18,789 clients), and two waves during post-expansion in 2015 (N = 106 programs, n = 17,339 clients) and 2017 (N = 94 programs, n = 16,191 clients). We relied on two path analyses to test differences between pre and post expansion on days to enter treatment (wait time) and days in treatment (retention), as well as mechanisms of change (coordinated care). We compared two multiple group negative binomial regression models to test race/ethnicity as moderators and coordinated care as mediating mechanisms.

**Results:** Compared to pre-Medicaid expansion and white clients, Latinx and African Americans reported shorter wait times to enter care in high-capacity programs post-expansion. African Americans’ retention was longer than whites in high-capacity programs post-Medicaid expansion. Additionally, receipt of HIV testing and coordination of mental health services played an indirect role in the relationship between high capacity programs and shorter wait time.

**Conclusion:** Medicaid expansion played a significant role in eliminating disparities in treatment access and retention in AHS. Program leadership, readiness for change and Medicaid acceptance are important capacity factors to implement coordinated mental health and HIV testing and increase treatment access among minorities. Future research should consider these program capacity factors to implement other public health services (e.g., COVID-19 testing) to mitigate the disproportionate impact of the pandemic on minority communities.

## A39 “Methadone treatment barriers in a small Midwest community” (HD15)

### Emily Pasman, Stella Resko, Michael Broman, Rachel Kollin, Jamey Lister, and Elizabeth Agius

#### Lead author affiliation: School of Social Work at Wayne State University, 5447 Woodward Avenue Detroit MI, 48202, USA

##### **Correspondence:** Emily Pasman (emily.pasman@wayne.edu)

*Addict Sci Clin Pract* 2020, **15(Suppl 2)**:A39

**Background:** Though effective medications for opioid use disorder exist, treatment barriers are prevalent in rural and small metropolitan communities (Lister et al. 2019). The purpose of this study is to (1) examine barriers to participation in methadone treatment in a small Midwest community and (2) identify factors associated with greater endorsement of barriers.

**Methods:** Clients receiving methadone treatment (N = 267) were recruited to complete a computer-based survey (December 2019). Surveys assessed socio-demographics (age, gender, race, education, community), substance use, depression and anxiety symptoms, trauma history and symptoms, recovery support, and barriers to treatment (e.g., childcare, work, housing, transportation, legal obligations, mental health). Descriptive statistics were used to examine individual barriers and a multivariate linear regression was calculated to assess predictors of greater cumulative barriers.

**Results:** Geographic and logistical issues were the most commonly endorsed barriers, with over one-third reporting challenges related to their work schedule (35%), distance from home to treatment (34%), and transportation (34%). Past year opioid use (B = 1.73, p = 0.017) and more severe mental health symptomology (B = 0.23, p = 0.017) were associated with greater numbers of barriers. Greater levels of recovery support were associated with fewer barriers (B = − 0.23, p = 0.001). No associations were found for demographic variables.

**Conclusion:** This study adds to the limited research on barriers to methadone treatment for clients in rural and small metropolitan communities. Individuals with more recent opioid use reported a greater number of barriers, suggesting barriers may be more substantial early on. Clients experiencing more depression and anxiety symptoms may be more vulnerable to treatment dropout, as evidenced by greater endorsement of barriers. As social support emerged as a potential protective factor against cumulative barriers to treatment, efforts to enhance family and peer support should be explored as adjunctive services to medication treatment.

## A40 “Quantifying the cascade of care for engagement in opioid use disorder treatment programs in an American Indian Tribal Nation in Minnesota” (HD16)

### Jordan Stipek, Thaius Boyd, Alex Kraft, Judge Muskrat, Kevin Hallgren, Clinton Alexander, and Brenna Greenfield

#### Lead author affiliation: University of Minnesota Duluth Medical School, 1035 University Dr, Duluth, MN 55812, USA

##### **Correspondence:** Jordan Stipek (stipe003@d.umn.edu)

*Addict Sci Clin Pract* 2020, **15(Suppl 2)**:A40

**Background:** The Cascade of Care (CoC; Williams et al. 2019) is a quantitative framework for measuring population-level OUD treatment engagement. Studies have quantified the CoC at state and provincial levels using existing public health datasets (Yedinak et al. 2019; Piske et al. 2020); however, none have quantified the CoC in a tribal context.

**Methods:** We evaluated whether a state dataset that tracked admissions and discharges from all public and private substance use disorder (SUD) treatment programs (DAANES; MN DHS 2020) could quantify the CoC for an American Indian tribal nation in MN. Analyses were restricted to American Indian individuals who were affiliated with a specific MN tribe, were admitted to or discharged from SUD programs in counties located within the tribal nation between 2017 and 2019, and reported opioids as their primary substance of use at admission. We evaluated whether the DAANES data could quantify the CoC steps, including those (1) at risk for OUD, (2) diagnosed with OUD, (3), who received medications for OUD, (4) retained in OUD treatment, and (5) who achieved OUD recovery.

**Results:** Of 614 admissions with identified opioids as the primary substance, 390 (63.5%) had moderate/severe OUD diagnoses (direct measure of CoC step 2). A majority (424 admissions; 69.1%) had medications for OUD planned as part of treatment, usually buprenorphine (indirect measure of CoC step 3). Limitations: Step 2 lacks people whose primary substance was another non-opioid drug but still had OUD; people with repeat admissions are counted multiple times; step 3 only indicates planned medications, not whether medications were prescribed or taken.

**Conclusions:** The DAANES data can provide some information about the CoC for AI/AN communities, including direct information about step 2 and indirect information about step 3. Additional data sources are needed to more adequately measure population-level OUD risk, treatment engagement, and recovery in tribal nations.

## A41 “Racial inequity in medication treatment for opioid use disorder: exploring potential facilitators and barriers to use” (HD17)

### Mara A.G. Hollander, and Julie Donohue

#### Lead author affiliation: University of Pittsburgh, 130 De Soto St, Pittsburgh, PA 15261, USA

##### **Correspondence:** Mara A.G. Hollander (mhollander@pitt.edu)

*Addict Sci Clin Pract* 2020, **15(Suppl 2)**:A41

**Background:** One in five individuals with opioid use disorder (OUD) receive medication treatment for OUD (MOUD). People receiving MOUD have lower risk of all-cause and overdose mortality. Black and Hispanic patients are less likely than white patients to initiate and continue MOUD, and racial disparities in MOUD remain largely unexplained. This study examines whether the relationship between race and initiation in MOUD is changed by contact with healthcare, human services, and criminal justice systems.

**Methods:** We used data from the Allegheny County Data Warehouse, which links person-level data from several of the county’s programs, including Medicaid, two court systems, the county jail, housing, and other social service programs. We examined the proportion of enrollees who initiated MOUD in the 180 days after an index OUD diagnosis.

**Results:** In Allegheny County, Pennsylvania, Black Medicaid enrollees with opioid use disorder are one-third less likely to start medication treatment than white Medicaid enrollees (26.7% vs. 43.0%, p < 0.001). We were able to explain approximately 25% of the difference by race in initiation of MOUD. Visits to the emergency department explained much of the variation. In a sensitivity analysis, days in jail also explained some of the racial differences in initiation.

**Conclusion:** Factors unrelated to the need for MOUD may impact initiation of MOUD and may explain variation by race. Our findings support current efforts to develop programs focused on initiation of MOUD in acute care facilities and criminal justice systems to improve rates of treatment overall and to reduce racial disparities.

## A42 “Social support among adults from rural and small urban communities in methadone treatment” (HD18)

### Michael Broman, Stella M. Resko, Emily Pasman, Suzanne Brown, Jamey Lister, and Elizabeth Agius

#### Lead author affiliation: School of Social Work at Wayne State University, 5447 Woodward Ave, Detroit, MI 48202, USA

##### **Correspondence:** Michael Broman (fk7765@wayne.edu)

*Addict Sci Clin Pract* 2020, **15(Suppl 2)**:A42

**Background:** Social support from family, friends, and others promotes retention in treatment, longer abstinence, and other positive outcomes for those in substance use treatment. Social support is especially critical during methadone treatment for opioid use disorder, due to stigmatization of this treatment modality (Cooper & Nielsen 2017). Little is known about social support for individuals receiving methadone in rural and small urban communities. This study examines factors associated with social support among adults receiving methadone treatment from a healthcare provider serving rural and small urban communities in Michigan.

**Methods:** Adults (N = 267) were recruited at a methadone clinic to complete a web-based survey. Social support was assessed with the Social Support for Recovery (Laudet et al. 2000) and Friends’ Support for Recovery (Humphreys et al. 1999) scales. Multiple regression was used to examine the association between social support and demographic variables, substance use, and stigma/shame (e.g. feelings of shame related to receiving methadone, frequency of hearing negative comments about methadone).

**Results:** The sample was 59.6% female, 40.4% male; 85.0% White, 15.0% person of color. Mean age was 38.51 years (SD = 9.95). Half (48.3%) misused opioids in the past year. Male gender, feelings of shame, and the frequency of hearing negative comments about methadone were inversely associated with recovery-specific support. Past-year shame and the frequency of hearing negative comments about methadone were inversely associated with support from friends.

**Conclusions:** Clients with stronger feelings of shame and who more frequently heard negative comments about methadone may also have lower levels of social support. These clients may be particularly vulnerable and need additional supports to maintain recovery. Interventions designed to enhance social support among individuals in methadone treatment may want to address shame and internal stigma. Those with higher levels of shame may benefit from interventions that address this feeling.

## A43 “Substance use and basic needs among methamphetamine and opioid involved mothers receiving public assistance in Kentucky” (HD19)

### Megan Dickson, and Carl Leukefeld

#### Lead author affiliation: University of Kentucky College of Medicine, 800 Rose Street MN 150, Lexington, KY 40506, USA

##### **Correspondence:** Megan Dickson (megan.dickson@uky.edu)

*Addict Sci Clin Pract* 2020, **15(Suppl 2)**:A43

**Background:** Despite national attention surrounding the opioid epidemic, recent reports suggest increases in methamphetamine use point to a “twin epidemic.” There is evidence of widespread methamphetamine use among opioid-involved individuals, yet little is known about high-risk populations who use both methamphetamine and opioids. Given this limitation, the current study examines substance use and basic needs among mothers with co-occurring methamphetamine and opioid use who receive public assistance.

**Methods:** Assessment data were collected from methamphetamine and opioid-involved mothers receiving public assistance in Kentucky (N = 2701) between July 2011 and June 2019. Participants were categorized into groups based on whether they reported only methamphetamine use (n = 616), only opioid use (n = 1660), or both methamphetamine and opioid use (n = 425) in the past 3 months. ANOVA and logistic regression were used to explore differences in demographics, substance use, and basic needs.

**Results:** Overall, more than one-third (38.5%) of participants reported using methamphetamine in the past 3 months, and, on average, were 30 years old with 2 children, mostly white (94.8%), unmarried (83.9%), unemployed (80.1%), and had completed high school (66.8%). Mothers who used both methamphetamine and opioids were more likely to be unemployed, live in a non-metropolitan community, have an incarceration history, and self-reported more days of mental health problems and pain. They were more substance-involved, more likely to have recently injected drugs, and reported experiencing difficulties related to a wider variety of basic needs in the past 3 months, such as transportation and housing.

**Conclusion:** Results suggest a high prevalence of methamphetamine use, while highlighting distinct differences related to methamphetamine and opioid involvement among mothers receiving public assistance. Implications for treatment policy and prevention include the importance of thorough assessments and intervention services that address the unique needs of mothers with co-occurring methamphetamine and opioid use who receive public assistance. Limitations include data collection in a single rural state.

## A44 “Addiction-related treatment service disparities in Middle Atlantic counties: state and urban–rural availability comparisons” (HD20)

### Jamey J. Lister, Kristen G. Powell, Andrew Peterson, Sarah Cooper, Stephen Crystal, and Peter C. Treitler

#### Lead author affiliation: Rutgers School of Social Work, 536 George St, New Brunswick, NJ 08901, USA

##### **Correspondence:** Jamey J. Lister (megan.dickson@uky.edu)

*Addict Sci Clin Pract* 2020, **15(Suppl 2)**:A44

**Background:** Middle Atlantic states have been hard hit by addictive disorders, conditions that co-occur with infectious diseases and mental health disorders. Thus, a comprehensive array of addiction-related treatment services is warranted. However, studies have yet to assess the availability of addiction-related treatment services and disparity patterns in the Middle Atlantic. Our objectives were to examine disparities between states and conduct urban–rural comparisons in the division and within states. We hypothesized rural disparities, and due to their rural composition, disparities for Pennsylvania and New York (compared to New Jersey).

**Methods:** We extracted data (May, 2020) from national directories (SAMHSA, CDC, NCPG) and calculated per-capita county-level availability statistics for seven addiction-related treatment services (opioid treatment programs; non-OTP substance use facilities; certified gambling counselors; HIV, HCV, HBV specialty and non-specialty facilities; mental health facilities). RUCCs categorized urban (RUCCs = 1–3) and rural (RUCCs = 4–9) counties. Analyses included one-way ANOVA and non-parametric tests.

**Results:** Of the 150 Middle Atlantic counties (PA = 67; NY = 62; NJ = 21), 36.0% (n = 54) were rural. In state comparisons, PA demonstrated disparities for facilities to treat HIV, HCV, and HBV (vs. NY or NJ) (Ps < 0.01). No additional state disparities were identified. Urban–rural disparities were revealed for each addiction-related treatment service. Rural counties experienced disparities for OTPs, certified gambling counselors, and facilities to treat HCV, HBV (Ps < 0.001), and HIV (P < 0.01). By contrast, urban counties demonstrated disparities for non-OTP substance use (P < 0.001) and mental health facilities (P < 0.01). State analyses demonstrated similar patterns, with more pronounced rural disparities in PA.

**Conclusion:** This analysis highlights nuanced availability of addiction-related treatment services in the Middle Atlantic. In line with predictions, rural county disparities were consistently demonstrated, though urban county disparities were identified. Service expansion efforts for rural counties, particularly in PA, are urgently needed.

## A45 “Substance use disorders in pregnancy and risk of severe maternal morbidity in the United States” (HD21)

### Marian Jarlenski, Elizabeth E. Krans, Qingwen Chen, Scott D. Rothenberger, Abigail Cartus, Kara Zivin, and Lisa Bodnar

#### Lead author affiliation: University of Pittsburgh, 130 De Soto St, Pittsburgh, PA 15261, USA

##### **Correspondence:** Marian Jarlenski (marian.jarlenski@pitt.edu)

*Addict Sci Clin Pract* 2020, **15(Suppl 2)**:A45

**Background:** Severe maternal morbidity and maternal mortality are increasing in the U.S. Reasons for the increase in severe maternal morbidity are not well elucidated, though the trend is not fully explained by increasing maternal age. Prior studies have suggested that the increase of chronic disease burden in pregnancy may explain the rise in severe maternal morbidity. However, the extent to which substance use disorders may contribute to the risk of severe maternal morbidity is unknown. We aim to determine the independent association between substance use disorders in pregnancy and risk of severe maternal morbidity in the United States. Data and Study Population: National Inpatient Sample, nationally representative sample of U.S. hospital discharges, 2003–2016. We included females ages 18–55 years of age with a hospitalization for labor and delivery.

**Methods:** We conducted a retrospective analysis of a weighted 53.4 million delivery hospitalizations. We constructed measures of substance use disorders using diagnostic codes for cannabis, opioids, and stimulants (amphetamines or cocaine) abuse or dependence during pregnancy. The outcome was a binary measure indicating a severe maternal morbidity, according to the CDC algorithm including 21 indicators; with and without blood transfusion. Using weighted multivariable logistic regression, we estimated the association between substance use disorders and adjusted risk of severe maternal morbidity. Because older age at delivery is predictive of severe maternal morbidity, we tested for effect modification between substance use and maternal age by age group (18–34 years vs > 34 years).

**Results:** Pregnant women with an opioid use disorder had an increased risk of severe maternal morbidity compared with women without an opioid use disorder (18–34 years: aOR: 1.51; 95% CI 1.41, 1.61, > 34 years: aOR: 1.17; 95% CI 1.00, 1.38). Compared with their counterparts without stimulant use disorders, pregnant women with a simulant use disorder (amphetamines, cocaine) had an increased risk of severe maternal morbidity (18–34 years: aOR: 1.92; 95% CI 1.80, 2.0, > 34 years: aOR: 1.85; 95% CI 1.66, 2.06). Cannabis use disorders were not associated with an increased risk of severe maternal morbidity. Results were consistent when considering severe maternal morbidity with and without blood transfusion.

**Conclusion:** Substance use disorders during pregnancy, particularly opioids, amphetamines, and cocaine use disorders, may contribute to severe maternal morbidity in the United States.

## A46 “Substance use trajectories among individuals on probation and the impact on re-arrest” (HD22)

### Jennifer Lerch, Scott T. Walters, and Faye S. Taxman

#### Lead author affiliation: George Mason University, 4400 University Drive MSN 6D3 Fairfax, VA 22030, USA

##### **Correspondence:** Jennifer Lerch (jlerch@gmu.edu)

*Addict Sci Clin Pract* 2020, **15(Suppl 2)**:A46

**Background:** Individuals cycling in and out of the criminal justice system disproportionately experience substance use disorders that result in a multitude of negative outcomes. While there is a growing body of research examining substance use trajectories, there are still large gaps in our knowledge, particularly among probationers. Using data from the Motivational Assessment Program to Initiate Treatment (MAPIT) multi-site, randomized controlled trial funded by NIDA (R01 DA029010-01), the current study examines the substance use patterns among individuals while on community supervision, with attention to the factors that predict membership into those substance use groups and how those substance use groups may predict re-arrest.

**Methods:** Self-report baseline and follow-up surveys and administrative data for 275 individuals are used to conduct group-based trajectory analysis, logistic regression, and survival analysis. Timeline Follow-Back data collected for 180 days post-baseline measured any illicit substance use and/or binge alcohol use, while re-arrest between 6- and 18-months post-baseline was captured dichotomously and as days until event from administrative records. **Results:** Six groups of substance users emerged from the data: abstainers, late-increasing, low-moderate, increasing, decreasing, and high user groups. The number of probation contacts, formal treatment attendance, number of arrests, and housing in a non-controlled environment were the time-varying predictors related to group membership, while risk-taking, family and peer drug use, initiating substance use under the age of 16, and severity of drug disorder were time-stable risk factors for group membership. Despite the distinct substance use patterns that emerged, the pattern of substance use did not predict later re-arrest among this group of individuals on community supervision.

**Conclusion:** Despite the distinct substance use patterns that emerged, the pattern of substance use did not predict later re-arrest. The patterns of substance use provide insights that could support interventions and justice controls to serve both health and justice goals.

## A47 “Syndemic barriers to successful treatment outcomes for individuals receiving medication for opioid use disorder” (HD23)

### Mary Kleinman, Christopher J. Seitz-Brown, Valerie D. Bradly, Hannah Tralka, Morgan Anvari, Thomas Cole, Annabelle Belcher, Aaron Greenblatt, and Jessica F. Magidson

#### Lead author affiliation: University of Maryland, College Park, College Park, MD 20742, USA

##### **Correspondence:** Mary Kleinman (mkleinm@umd.edu)

*Addict Sci Clin Pract* 2020, **15(Suppl 2)**:A47

**Background:** Successful engagement with and retention in medication treatment for opioid use disorder (MOUD) is an important focus in the fight against the opioid crisis. Gaps in OUD care point to a need for improved understanding of factors that affect MOUD outcomes and how barriers may act as syndemic factors, compounding one another’s effects. This study used qualitative methods to solicit feedback about barriers to retention and successful treatment outcomes in MOUD.

**Methods:** This study was conducted at a community-based drug treatment center that serves a low-income, minority population. We recruited patients and staff as well as peer recovery coaches who work in OUD recovery across Baltimore City. Semi-structured interviews and focus groups asked about factors that influence MOUD treatment outcomes and how barriers co-occur and interact. We used thematic analysis to examine themes pertaining to our research questions and two independent coders coded each transcript based on identified themes.

**Results:** Participants (n = 20) had a mean age of 48.4 (SD = 10.0), 70% male, and 60% Black or African American. Mean reported age of first drug use was 17.7 (SD = 5.1). Staff and peer recovery coach (PRC) participants (n = 12) had a mean age of 49.2 (SD = 0.7), were 42% male, 75% Black or African American, with an average of 9.6 years working in substance use treatment. Barriers described by participants fell into four overarching but cross-cutting levels: individual/self, social/interpersonal, institutional/structural, and community/environmental. Participants described co-occurrence of these barriers as fueling one another and having a disastrous effect on treatment outcomes.

**Conclusions:** Understanding barriers to successful MOUD outcomes experienced by this vulnerable population and considering the synergistic effect of these barriers may assist with identification and promotion of the types of interventions needed to effectively and efficiently mitigate their impact.

## A48 “The role of stigma in care-seeking experiences for people with hepatitis C who inject drugs” (HD24)

### Elizabeth Austin, Emily C. Williams, Michael Barry, Elyse Tung, Sara N. Glick, Michael Ninburg, and Judith I. Tsui

#### Lead author affiliation: Surgical Outcomes Research Center at University of Washington, 1107 NE 45th Street, Suite 502, Seattle, WA 98105, USA

##### **Correspondence:** Elizabeth Austin (austie@uw.edu)

*Addict Sci Clin Pract* 2020, **15(Suppl 2)**:A48

**Background:** Stigma, or systemic devaluing of groups based on features that distinguish them from societal norms, is common for persons with substance use disorders. For people who inject drugs (PWID), stigma may influence experiences navigating care for hepatitis C as well as care for other pressing health needs.

**Methods:** As part of a broader study focused on developing a community pharmacy model of medication delivery, we conducted semi-structured qualitative interviews to understand PWID’s care-seeking experiences. Participants were recruited from Seattle-area community organizations and eligible if they had recent (≤ 3 months) injection drug use and hepatitis C. Interviews were transcribed and analyzed using a Rapid Assessment Process, where two independent coders summarized learnings using structured templates and iterative review to identify themes. Here we report emergent themes specific to experiences of stigma.

**Results:** Among 40 participants, 65% were male, 53% were White, and most (80%) were not stably housed; mean frequency of injection use was daily. Participants reported internalized stigma, which, when paired with the interpersonal stigma (e.g. stigmatizing language and behaviors) enacted by care team members, created barriers to how PWID express their care preferences and felt heard by providers. As PWID navigated care, their status as an active user of drugs was used to control and sometimes coerce their access to services (for instance through court-mandated treatment), further minimizing the value of individual preferences for care and reinforcing perceived stigma. Consequently, the experience of stigma discouraged PWID from seeking needed care.

**Conclusions:** This study underscores the pervasive role stigma has throughout PWID’s experiences with healthcare and its impact on PWID’s ability to navigate the care they need. Multilevel interventions that target the individual, interpersonal, community, and structural levels of stigma associated with injection drug use may be needed to mitigate negative impacts on PWID health outcomes.

## A49 “Trends in gabapentin utilization among Medicare beneficiaries, 2006–2015” (HD25)

### Abisola Olopoenia, Sean P. Fleming, and Linda Simoni-Wastila

#### Lead author affiliation: University of Maryland School of Pharmacy, 20N Pine St, Baltimore, MD 21201, USA

##### **Correspondence:** Abisola Olopoenia (aolopoenia@umaryland.edu)

*Addict Sci Clin Pract* 2020, **15(Suppl 2)**:A49

**Background:** Little is known about the trends in gabapentin utilization in the Medicare population. Our goal was to characterize gabapentin utilization among Medicare beneficiaries overall, and among specific clinical [disabled, chronic pain (CP), mental health disorders (MHD), substance use disorders (SUD), seizures, post-herpetic neuralgia, neuropathic pain and opioid or/and benzodiazepine users] subgroups.

**Methods:** We conducted a retrospective longitudinal analysis using a 5% sample of 2006–2015 Medicare data. We included fee-for-service beneficiaries with ≥ 1-month coverage during each year within our study period. Clinical conditions were defined based on a diagnosis of CP (back, neck, joint, head or chronic pain), MHD (anxiety, depression, personality disorders, mood disorders, adjustment disorders, and attention deficit hyperactive disorders), SUD (alcohol, opioid or non-opioid) and neuropathic pain. Opioid or/and benzodiazepine utilization was defined as ≥ 2 prescriptions for either/both medication(s) in each calendar year. We compared the annual prevalence of gabapentin utilization overall, and among clinical subgroups.

**Results:** The study included 9.3 million person-years between 2006 and 2015. The percentage of individuals who utilized gabapentin increased from 5.38% (95% CI 5.33–5.42) in 2006 to 11.68% (95% CI 11.62–11.75) in 2015 (p < 0.0001), an increase of 112%. All subgroups experienced increased gabapentin utilization between 2006 and 2015; however, the increase in gabapentin utilization was highest among disabled individuals (8.15% to 18.48%), opioid users (13.64% to 27.48%) and those with SUD (12.17% to 28.39%). Gabapentin utilization remained high among benzodiazepine users (19.03% in 2013 to 21.58% in 2015) and opioid and benzodiazepine users (28.05% in 2013 to 31.82% in 2015.)

**Conclusion:** Gabapentin utilization has increased significantly over the years, especially among high risk sub-groups. Given emerging evidence concerning gabapentin’s association with respiratory depression, it is essential we comprehensively examine its ongoing utilization, especially among those who may be potentially more vulnerable to these adverse effects.

## A50 “Veterans’ treatment courts in Kentucky: examining how personal characteristics and during-program occurrences influence program completion and criminal recidivism” (HD26)

### Monica Himes, Lisa Shannon, and Elizabeth Nichols

#### Lead author affiliation: Morehead State University, 150 University Blvd, Morehead, KY 40351, USA

##### **Correspondence:** Monica Himes (m.himes@moreheadstate.edu)

*Addict Sci Clin Pract* 2020, **15(Suppl 2)**:A50

**Background:** Military veterans are disproportionately represented in United States (U.S.) jails and prisons, with nearly 10% of current inmates being veterans. Veterans’ criminal justice involvement is often precipitated by underlying mental health and substance abuse that are connected to their military service. Veterans’ treatment courts are the judicial response to a need for more coordinated provision of mental health and substance abuse services to veterans involved in the criminal justice system. Modeled after drug courts and mental health courts, veterans treatment courts are a judicial innovation that aim to honor the service of veterans by providing them an alternative to incarceration.

**Methods:** There are currently 551 veterans' treatment courts in 42 states throughout country, including five in Kentucky. This exploratory descriptive study uses Andersen’s healthcare utilization model and a social control theoretical perspective as a framework to examine veterans’ treatment court outcomes from a sample of participants (N = 58) in Kentucky. Univariate and bivariate analyses were used to provide a description of the sample and to examine relationships between personal characteristics and during-program occurrences and the outcomes of program completion and criminal recidivism.

**Results:** Gender, sanctions, drug screens, and treatment sessions each had a significant association with program completion, and both age and housing status had a significant association with recidivism.

**Conclusion:** Findings for each outcome variable are discussed, along with possible explanations, as well as limitations of the study, implications of this research for social work practice, and suggestions for future research.

## A51 “Assessing overdose education and naloxone distribution within syringe service programs throughout the United States” (IS01)

### Barrot H. Lambdin, Ricky Bluthenthal, Bryan Garner, Lynn Wenger, Savannah O’Neill, and Alex H. Kral

#### Lead author affiliation: RTI International, 307 Waverley Oaks Rd #101, Waltham, MA 02452, USA

##### **Correspondence:** Barrot H. Lambdin (blambdin@rti.org)

*Addict Sci Clin Pract* 2020, **15(Suppl 2)**:A51

**Background:** Syringe service programs (SSPs) have been a linchpin for public health efforts such as HIV and HCV prevention for people who use drugs (PWUD), and SSPs have led efforts for community-based overdose education and naloxone distribution (OEND). SSPs are ideal venues to deliver OEND for PWUD, with staff who excel in providing culturally appropriate services and delivery systems designed to reach people where they are. We describe phases of OEND implementation within SSPs and assess predictors of SSP-based OEND penetration throughout the United States.

**Methods:** In collaboration with the North American Syringe Exchange Network and Harm Reduction Coalition, we surveyed all known SSPs in the United States in 2019. Out of the 342 known SSPs operating, 266 (77%) responded to the online survey. We utilized negative binomial regression to assess predictors of SSP-based OEND penetration (i.e., number of naloxone doses distributed), adjusting for opioid overdose deaths in the region.

**Results:** Regarding OEND implementation phases, 3% of SSPs were exploring OEND implementation, 3% were actively preparing for OEND implementation, 28% implemented OEND for < 12 months, and 66% had implemented OEND for ≥ 12 months. In the prior year, 237 SSPs reported distributing 710,232 naloxone doses, including refills, to 230,506 people. We found that penetration of SSP-based OEND varied substantially by census division (p < 0.001). In addition, we found that proactive naloxone refill systems (adjusted Incidence Rate Ratio (aIRR = 2.77; p = 0.002) and higher levels of community support (aIRR = 2.54; p = 0.039) were associated with significantly higher rates of naloxone doses distributed. SSPs located in regions with larger numbers of opioid overdose deaths did not have significantly higher levels of naloxone distribution.

**Conclusions:** While our results showed high levels of OEND integration within SSPs in the United States, OEND penetration varied substantially across census divisions, and substantial opportunity existed for improving penetration of SSP-based OEND. Public health initiatives to reduce opioid overdose deaths should increase investments that support and scale-up SSPs and SSP-based OEND services throughout the United States.

## A52 “Boosting interest and adoption of technology by substance use disorders treatment & recovery support providers through a brief online learning and consultation series on text messaging” (IS02)

### Terra Hamblin, Joyce Hartje, Carina Rivera, Trisha Dudkowski, Nancy Roget, and Scott Walters

#### Lead author affiliation: Center for the Application of Substance Abuse Technologies at University of Nevada, Reno, 1664N Virginia St, Reno, NV 89557, USA

##### **Correspondence:** Terra Hamblin (thamblin@casat.org)

*Addict Sci Clin Pract* 2020, **15(Suppl 2)**:A52

**Background:** Text messaging is a popular and widely used smartphone feature. Research demonstrates the effectiveness of text messages in supporting individuals with chronic diseases and mental health conditions to better manage their symptoms. Following this trend, recent studies found text messaging helped individuals with substance use disorders (SUD) to increase engagement in treatment and/or recovery support services, receive educational resources, and learn to manage craving and negative thoughts/moods. Unfortunately, SUD treatment/recovery support providers’ uptake of text messaging has been slower than other health providers.

**Methods:** A series of online learning and consultation strategies were implemented to increase uptake of text messaging by SUD treatment/recovery support providers. First, a step-by-step guide was created to help providers use text messages to enhance and expand the reach of their services. Next, the guide was used to structure a 4-session virtual initiative called Text Reminders to Assist Clinical Effectiveness and Recovery (TRACER), designed to teach providers how to: set up a texting sequence that supports treatment flow; use a texting provider to send messages that support the treatment sequence; send texts that are positively-framed, direct, and personalized; ensure the messaging strategy is compatible with legal/ethical guidelines; and use different formats for texting sequences. Third, at the end of the 4-sessions providers had an opportunity to have a 1-h individual consultation session to discuss implementation of text messaging at their agency.

**Results:** Responses were 100% positive regarding the overall TRACER experience. Quantitative and qualitative findings will be presented about the usefulness of the texting guide, format/content of the virtual sessions, and successes/challenges to implementing text messaging. **Conclusion:** A brief online learning and consultation series can be used as a vehicle to boost SUD treatment and recovery support providers use of technology and serve as a vehicle to intervention adoption.

## A53 “Boosting interest and adoption of technology by substance use disorders treatment & recovery support providers through a brief online learning and consultation series on text messaging” (IS03)

### Courtney M. DelaCuesta, Rebecca Uth, Linda Hurley, and Rosemarie Ann Martin

#### Lead author affiliation: Brown University School of Public Health, 121 S Main St, Providence, RI 02903, USA

##### **Correspondence:** Courtney M. DelaCuesta (Courtney_DelaCuesta@brown.edu)

*Addict Sci Clin Pract* 2020, **15(Suppl 2)**:A53

**Background:** Rates of opioid use disorder (OUD) have risen at an alarming rate, with overdose deaths peaking nationally in 2017. More than 200,000 individuals with an OUD are involved with the criminal justice system yearly. Within the first 2 weeks post-release, unintentional fatal overdoses spike, as individuals lose their tolerance during incarceration. Medication for opioid use disorder (MOUD) has been shown to significantly decrease relapse and recidivism, while increasing retention in treatment. In 2016, upon the introduction of the Governor’s Overdose Prevention and Intervention Task Force, Rhode Island (RI) became the first state to implement a state-wide medication assisted treatment (MAT) program within the correctional system, offering all three FDA-approved MOUD to incarcerated individuals.

**Methods:** Linkage to MOUD treatment post-release has been expanded into the community, leading to a significant decrease in statewide fatal overdose incidents post-release in RI. The program aims to target individuals who are at even higher risk for relapse/overdose post-release, providing continuity of treatment and support for clients as they are reintroduced into the community.

This program partners Brown University, CODAC Behavioral Healthcare, RI State Police, RI District Court, and RI Department of Corrections to increase adherence to MAT through an intensive post-release support protocol, implemented by a multidisciplinary outreach team. A clinician, a member of local law enforcement, and a peer navigator collaborate to provide case management, recovery support services, and referrals.

**Results:** We have enrolled 142 individuals, and provided resources including transportation, client/family emotional support, and referrals to behavioral health providers and MAT programs. The multidisciplinary nature of each unit has allowed clients to bypass traditional barriers in continuity of treatment.

**Conclusion:** By ensuring access to MAT for criminal justice-involved individuals, levels of relapse/overdose are expected to decline, leading to lower crime, more intact families, increased employment, and improved community engagement.

## A54 “Exploring mechanisms of change for the implementation & sustainment facilitation (ISF) strategy” (IS04)

### Bryan R. Garner, and Stephen J. Tueller

#### Lead author affiliation: RTI International, 3040 E Cornwallis Rd, Durham, NC 27709, USA

##### **Correspondence:** Bryan R. Garner (bgarner@rti.org)

*Addict Sci Clin Pract* 2020, **15(Suppl 2)**:A54

**Background:** Experimental evidence supports the Implementation & Sustainment Facilitation (ISF) strategy as an effective adjunct to the Addiction Technology Transfer Center (ATTC) strategy for improving implementation of a motivational interviewing-based brief intervention for substance use within HIV service organizations. However, beyond the need to identify effective implementation strategies is the need to understand the mechanisms of change for implementation strategies. This study explored the extent to which changes in implementation climate and readiness were mechanisms of change for the ISF strategy.

**Methods:** Thirty-nine HIV service organizations and their staff (n = 78) were randomized to receive the ATTC strategy or the ATTC + ISF strategy. Implementation climate (the extent to which implementation was expected, supported, and rewarded) and implementation readiness (the extent to which staff had the necessary training, knowledge, skill, and time for implementation) were assessed prior to randomization and after the project’s implementation phase. Adjusted multilevel regression analysis was used to examine the extent to which the implementation effectiveness of staff could be predicted by changes in implementation climate and implementation readiness.

**Results:** The ISF strategy remained a significant predictor of implementation effectiveness in all models. In addition, variation in implementation effectiveness was explained by changes in implementation readiness (β = 0.14, p = 0.02), but not by changes in implementation climate (β = 0.05, p > 0.05). Regarding the impact of the ISF strategy on these two putative mediators, the ISF strategy was found to have a significant impact on changes in implementation climate (β = 0.83, p = 0.004), but did not quite reach statistical significance regarding changes in implementation readiness (β = 0.65, p = 0.09).

**Conclusions:** Evidence suggests the ISF strategy improved implementation climate and implementation effectiveness, yet neither implementation climate nor implementation readiness was able to be supported as a potential mechanism of change.

## A55 “How are substance use disorder treatment programs in arkansas responding to COVID-19? A qualitative study” (IS05)

### Jure Baloh, and Geoffrey M. Curran

#### Lead author affiliation: University of Arkansas for Medical Sciences, 4301W Markham St, Little Rock, AR 72205, USA

##### **Correspondence:** Jure Baloh (jbaloh@uams.edu)

*Addict Sci Clin Pract* 2020, **15(Suppl 2)**:A55

**Background:** The COVID-19 pandemic rapidly changed how substance use disorder (SUD) treatment services are organized and provided. This study examines what changes SUD treatment programs in Arkansas implemented (e.g., guidelines, technologies), and what factors influenced their ability to implement and sustain these changes.

**Methods:** We conducted semi-structured phone interviews with SUD program leadership (administrative and/or clinical leaders). Interviews are based on the Consolidated Framework for Implementation Research and focus on what changes programs are implementing, barriers and facilitators to implementation, and recommendations. Interviews were recorded and transcribed verbatim and are currently being thematically analyzed (preliminary analyses are presented). **Results:** We interviewed 29 leaders at 21 residential and outpatient SUD treatment programs. Preliminary analyses indicate that programs implemented similar infection control practices: COVID-19 screening at entry, use of masks, hand hygiene, and social distancing (including working from home, when possible). To limit contacts between different groups (clients, staff, visitors), residential programs also discontinued outside visitations and some capped admissions; outpatient programs discontinued group sessions or switched to telehealth and, when possible, switched individual sessions to telehealth (some continued in person, e.g. for client preference, drug screening). Key facilitators included grants/loans to help finance salaries, equipment, and service extension, looser regulatory restrictions (e.g. telehealth, prior approval), and good communication and coordination with other organizations (e.g., state agencies, partners, peers). Key barriers included limited access to supplies (e.g., masks, disinfectants), lack of rapid COVID-19 testing (particularly for clients entering residential treatment), limited capacity for social distancing, and negative employee and client responses (e.g., anxiety, quitting). Considerable uncertainty and concerns remain about sustainability of new practices, including long-term economic viability of SUD programs, and their ability to meet clients’ treatment and support needs. Key recommendations include better access to supplies, rapid COVID-19 testing, telehealth continuation (including beyond the pandemic), and strengthened communication within and between organizations to enhance resilience of the SUD services system.

**Conclusion:** This study provides an early insight into how SUD programs are responding to COVID-19. Future studies can quantify the extent/prevalence of identified themes and examine what strategies can help sustain the implemented changes.

## A56 “Impact of therapist attitudes toward evidence-based treatments on training participation” (IS06)

### Lila Bruynesteyn, Molly Bobek, Aaron Hogue, Alexandra MacLean, and Nicole Porter

#### Lead author affiliation: Center on Addiction, 485 Lexington Ave, New York, NY 10017, USA

##### **Correspondence:** Lila Bruynesteyn (lbruynesteyn@centeronaddiction.org)

*Addict Sci Clin Pract* 2020, **15(Suppl 2)**:A56

**Background:** Research has shown that attitudes such as pre-training anxiety, pre-training motivation, and perceived organizational support towards Evidence-Based Treatments (EBTs) predict learning and clinical use of EBTs. Learning is also influenced by perceived barriers—minimizing organizational and perceptual barriers to EBT implementation has demonstrably improved EBT training outcomes. Finally, differences in therapists’ self-reported allegiance to various therapeutic orientations may impact the degree to which they engage in trainings for specific therapy frameworks like family therapy (FT).

**Methods:** This study tested provider attitudes toward EBTs, allegiance to FT, and perceived barriers to delivering FT in predicting training uptake in terms of perceptions of training utility and rate of weekly training module completion. Training included weekly 20-min online training sessions in which therapists viewed and coded short videos exemplifying expert use of FT techniques. We hypothesized that stronger attitudes toward EBTs, greater allegiance to FT, and fewer perceived barriers to delivering FT would predict greater training uptake. Providers were a diverse group of frontline therapists treating adolescent behavior problems.

**Results:** Multiple linear regression was used to assess the relation between attitudes toward EBTs, FT allegiance, and perceived barriers and participation in training activities. Results indicated therapists who endorsed more positive attitudes toward EBTs in terms of Openness to Innovation and Intuitive Appeal reported greater utility of the training system. Furthermore, allegiance to FT predicted a higher rate of training module completion. Contrary to hypotheses, attitudes towards Organizational Requirements, Perceived Divergence of Research-based Innovation, and perceived barriers to implementing FT did not significantly predict outcomes.

**Conclusion:** Positive attitudes towards EBTs, and greater proficiency and allegiance to FT are important for FT training uptake. Providers treating adolescent substance use who are more open to trying new interventions and who endorse a stronger allegiance to FT may be more likely to engage in novel FT online video-based training opportunities.

## A57 “Influence of opioid treatment provider attitudes on skill and knowledge after contingency management training” (IS07)

### Kimberly Yap, Daneris Fuentes, Samantha Moul, Cara Murphy, Bryan Hartzler, Bryan R. Garner, and Sara J. Becker

#### Lead author affiliation: Brown University School of Public Health, 121 S Main St, Providence, RI 02903, USA

##### **Correspondence:** Kimberly Yap (kimberly_yap@brown.edu)

*Addict Sci Clin Pract* 2020, **15(Suppl 2)**:A57

**Background:** Contingency management (CM) is an evidence-based behavioral treatment in which patients have the opportunity to earn motivational incentives for achieving treatment goals. One potential barrier to the successful implementation of CM in opioid treatment programs (OTPs) is negative provider attitudes towards CM. Training can potentially help improve the implementation of CM in OTPs, but the relationship between provider attitudes towards CM and responsiveness to training is not well understood. Using data from the first cohort of a multi-site type 3 hybrid trial, this study evaluates the relationship between CM knowledge and skills, as well as the extent to which providers’ CM attitudes predicted both CM knowledge and skills after training.

**Methods:** Thirty-nine treatment providers from eight OTPs completed a CM Attitudes survey, which assessed provider attitudes regarding the acceptability and effectiveness of CM. Providers then completed an 8-h CM training. After the training, 35 providers completed an 18-item CM Knowledge Test to assess their knowledge of CM principles and 30 providers submitted a CM session role-play to assess their CM skills.

**Results:** Bivariate analyses supported the a priori hypothesis that there would be a statistically significant correlation between CM knowledge and skill (r = 0.50, *p* = 0.007). Multivariate regression supported the hypotheses that CM attitudes would predict both CM knowledge (b = 0.47, p = 0.005) and CM skill (b = 0.56, p = 0.002), even when controlling for providers’ age and months of clinical experience.

**Conclusion:** Providers with more negative CM attitudes were less responsive to CM training, suggesting that pre-training attitudes represent a novel training target. Providers with negative CM attitudes may benefit from customized training to address CM misconceptions. Overall, the study findings highlight the importance of considering counselors’ attitudes when designing CM training in order to better address the barriers surrounding CM implementation.

## A58 “Intervention to increase naloxone engagement in community pharmacies: a focus group investigation” (IS08)

### Daniel Ventricelli, Mary Gray, Jesse Boggis, Jef Bratberg, Adriane Irwin, Caleb Banta-Green, Gillian Leichtling, Anthony Floyd, Dan Hartung, and Traci C. Green

#### Lead author affiliation: University of the Sciences in Philadelphia, 600 S 43rd St, Philadelphia, PA 19104, USA

##### **Correspondence:** Daniel Ventricelli (d.ventricelli@usciences.edu)

*Addict Sci Clin Pract* 2020, **15(Suppl 2)**:A58

**Background:** Community-based pharmacies play a pivotal role towards improving opioid safety by dispensing naloxone, medications for opioid use disorder and selling nonprescription syringes for safe injection. This study explored pharmacist attitudes, knowledge, and experience in dispensing naloxone, providing buprenorphine and selling nonprescription syringes as well as the acceptability of a pharmacy-based training program, entitled RESPOND TO PREVENT, aimed at improving these three content areas to reduce opioid-related harms.

**Methods:** Two online asynchronous focus groups were conducted with community-based chain pharmacists (n = 32) across Massachusetts, New Hampshire, Oregon, and Washington state. Eligible participants were those pharmacists who had completed the baseline assessment and online course. Each pharmacist participated anonymously for approximately 30 min across a 52–60 h window. Questions focused on prescriber support, policy impacts at the store, state, and federal level, experiences with pharmacy-based naloxone, and intervention implementation barriers and facilitators. Qualitative data analysis was conducted by a multidisciplinary team using an immersion-crystallization approach.

**Results:** Five major themes were identified in the focus groups: (1) gaps in pharmacist and broader pharmacy team knowledge of opioid use disorder, pharmacy-based naloxone, buprenorphine and syringe safety and sales; (2) shifts in self-efficacy to initiate “tough” conversations with patients and counseling about naloxone using intervention materials; (3) attitude changes regarding the pharmacist’s role as community caretaker; (4) practice changes to increase provision of naloxone and syringe sales; and (5) barriers to naloxone provision due to cost concerns and stigma towards people who use drugs.

**Conclusion:** Community pharmacists across four states identified important knowledge, training, and stigma-related gaps. Results reflect rich and positive experiences of community-based pharmacists participating in the educational intervention and provide face validity for the content of the modules and intervention materials as a means of addressing identified gaps.

## A59 “Learning extenders: using text messages to enhance online learning for the behavioral health workforce” (IS09)

### Joyce Hartje, Terra Hamblin, Carina Rivera, Trisha Dudkowski, Wendy Woods, Kim Prokosch, Jaycob Nolte, Nancy Roget, Thomas Freese, Beth Rutkowski, and Scott Walters

#### Lead author affiliation: Center for the Application of Substance Abuse Technologies at University of Nevada, Reno, 1664N Virginia St, Reno, NV 89557, USA

##### **Correspondence:** Joyce Hartje (jhartje@casat.org)

*Addict Sci Clin Pract* 2020, **15(Suppl 2)**:A59

**Background:** Research on adult learning, evidence-based practices adoption/implementation, and workplace learning shows that training alone rarely changes practice behavior. To align with these tenets, it was posited that a new training/technical assistance model linking training to other services (e.g., expert consultation, performance feedback, peer support, reminders, and case studies) could dramatically improve training outcomes. The new model, called Workwise, is an online series that provides participants interactive instructional/consultation activities, including Learning Extenders, which are brief text messages that include reminders, prompts, questions, and links to videos or websites to increase learning, retention, and skills.

**Methods:** Each Workwise series is an online interactive training and consultation initiative on treatment/recovery-related topics. The series involves a higher level of learning intensity and commitment than other training formats and participants receive approximately four Learning Extender reminders each week. Participants complete pre-post-follow-up web-based surveys to assess the technology-based training and the impact of the Learning Extenders on changing and/or enhancing their learning experience.

**Results:** Overall, participants said the Learning Extenders were useful in reminding them to complete learning activities, keeping them engaged in the sessions, and helping them apply the materials. Most also said they would recommend using text messages in future trainings and that it was beneficial to their learning experience. Qualitative and quantitative data will be presented.

**Conclusion:** Adding text-based Learning Extenders to a series of sequenced learning activities further enhances the training experience and improves training outcomes.

## A60 “Maximizing implementation of contingency management in opioid treatment programs: process data from the first cohort of a type 3 hybrid trial” (IS10)

### Sara J. Becker, Bryan R. Garner, Bryan Hartzler, Cara Murphy, Carla Rash, Kimberly Yap, Julia Yermash, Elizabeth Ball, Sharon Lang, Mat Roosa, and Lynn Madden

#### Lead author affiliation: Brown University School of Public Health, 121 S Main St, Providence, RI 02903, USA

##### **Correspondence:** Sara J. Becker (sara_becker@brown.edu)

*Addict Sci Clin Pract* 2020, **15(Suppl 2)**:A60

**Background:** Contingency management (CM) is the most effective adjunctive treatment to medication for opioid use disorders, but its implementation in opioid treatment programs (OTPs) remains low. Project MIMIC (Maximizing Implementation of Motivational Incentives in Clinics) is a type 3 hybrid trial testing strategies to help OTPs implement CM. This poster reports process data from the project’s first cohort of OTPs.

**Methods:** Staff and patients from eight OTPs were cluster randomized to receive either the Addiction Technology Transfer Center (ATTC) strategy (workshop + feedback + coaching) or the Enhanced ATTC (EATTC) strategy, which layered in two additional strategies: Pay-For-Performance and Implementation Sustainment Facilitation. Consistent with the exploration, preparation, implementation, and sustainment (EPIS) framework, OTPs engaged in 5 months of preparation and 7 months of implementation.

**Results:** Each OTP completed preparation activities and advanced to the implementation phase. During the preparation phase, 52 staff (28 ATTC, 24 EATTC) completed a baseline survey. Of those enrolled, 41 (75% ATTC, 83% EATTC) staff participated in a didactic CM workshop and 31 (54% ATTC, 67% EATTC) submitted a role play for performance feedback. During the implementation phase, each OTP sought to enroll 25 patients. Overall, OTPs in the ATTC condition recruited 88% of the target, while OTPs in the EATTC condition recruited 100%. Of the 58 CM session recordings submitted for feedback, 29 met the skills benchmark: 8 ATTC and 21 EATTC.

**Conclusions:** Preliminary process data indicate that CM recruitment, training engagement, and session submissions were greater in the experimental EATTC condition, relative to the ATTC control condition. Next steps will include examining the impact of the EATTC strategy on (a) number of CM sessions implemented with patients, (b) reductions in patient’s days of opioid use at follow-up, and (c) OTPs sustainment of CM during the 6-months post-implementation.

## A61 “Opioid treatment professional stigma as a barrier to contingency management implementation” (IS11)

### Kelli Scott, Cara M. Murphy, Kimberly Yap, Samantha Moul, Julia Yermash, and Sara J. Becker

#### Lead author affiliation: Brown University School of Public Health, 121 S Main St, Providence, RI 02903, USA

##### **Correspondence:** Kelli Scott (kelli_scott@brown.edu)

*Addict Sci Clin Pract* 2020, **15(Suppl 2)**:A61

**Background:** Contingency management (CM) is an evidence-based practice for opioid use disorder (OUD) that enhances the provision of usual care (i.e. medication for OUD) through rewarding patients for achieving treatment goals. However, CM faces numerous barriers to successful implementation in opioid treatment programs. Health professional stigma toward individuals with OUD is one important barrier that may limit treatment providers willingness to implement CM.

**Methods:** We assessed CM familiarity and implementation barriers and facilitators through qualitative interviews (N = 43) with treatment providers and leaders across 11 opioid treatment programs. De-identified interviews were transcribed and coded by two independent coders, with each coder completing half the interviews. A third coder coded 20% of the interviews to ensure inter-rater reliability and both a priori and emergent themes were analyzed.

**Results:** Stigma toward individuals with OUD was prominent in the transcripts, with 86% of transcripts having stigmatizing language (i.e. “substance abuser,” “clean/dirty,” or “addict”) or themes. Several emergent themes related to stigma were identified, including: (a) distrust of individuals with OUD; (b) infantilizing views; (c) belief that individuals with OUD don’t deserve prizes; and (d) recognition of individuals with OUD having self-stigma and community-based stigma.

**Conclusion:** These emergent themes highlight the importance of stigma as a key target for implementation strategies to facilitate sustained CM use in opioid treatment programs. Strategies to enhance CM and other evidence-based practice implementation in community opioid treatment programs likely need target multiple types of stigma (i.e. language, infantilizing views, and individual self-stigma) in order to facilitate effective scale up.

## A62 “Optimizing strategies to reduce initiation of prescription opioid analgesics to opioid naïve patients in primary care: study design and protocol” (IS12)

### Jan Klimas, Michee-Ana Hamilton, Greg Carney, Ian R. Cooper, Nicole Croteau, Huiru Dong, Colin Dormuth, Malcolm Maclure, M. Eugenia Socías, Lianping (Mint) Ti, Evan Wood, and Rita McCracken

#### Lead author affiliation: British Columbia Centre on Substance Use, 1045 Howe St Suite 400, Vancouver, BC V6Z 2A9, Canada

##### **Correspondence:** Jan Klimas (jan.klimas@bccsu.ubc.ca)

*Addict Sci Clin Pract* 2020, **15(Suppl 2)**:A62

**Background:** Describe the proposed design of and baseline data for a study using individualized prescribing portraits to reduce inappropriate initiation of prescription opioid analgesics to opioid naïve patients in primary care.

**Methods/design:** A mixed methods cluster randomized controlled trial tests a quality improvement initiative on safer opioid prescribing in primary care. The monthly incidence of opioid initiation is the primary outcome and is estimated using administrative data from a Centralized Medication Monitoring Database in British Columbia, Canada. Opioid naïve patient status is defined as no opioid prescriptions in the past 6 months. Secondary outcomes include related health outcomes (e.g., hospitalization, emergency department visit), and the intervention experience among a purposive sample of physicians and patients. Communities are randomized by geographical location and physicians receive either an “early” portrait (intervention group) or “delayed” portrait (control group) 1 year later.

**Results:** Between December 2018 and November 2019, a total of 5657 active family physicians initiated 139,145 opioid prescriptions to opioid-naïve patients. The mean monthly initiation rate was 2.05 prescriptions per physician. Majority of initiations were in the Lower Mainland regions of BC, also where the population is most concentrated, (46,456, 33% in the Fraser region), by prescribers who graduated between 1996–2010 (49,314, 49%), and had less than 10 visits per day (72,506, 52%).

**Conclusions:** We identified high numbers of initiation of opioid prescriptions to opioid naïve patients at baseline. The trial will provide important information on the potential of a complex educational intervention to change policy and practice on initiation of prescription opioid analgesics.

## A63 “Staff perceptions about implementing medication for opioid use disorder in outpatient public mental health settings” (IS13)

### Sarah Hunter, Erika Litvin Bloom, Allison J. Ober, Isabel Leamon, and Alanna Montero

#### Lead author affiliation: RAND Corporation, 1776 Main St. Santa Monica, CA 90401-3208, USA

##### **Correspondence:** Sarah Hunter (shunter@rand.org)

*Addict Sci Clin Pract* 2020, **15(Suppl 2)**:A63

**Background:** Most people in the U.S. with co-occurring opioid use disorder (OUD) and serious mental illness are treated primarily in mental health settings and do not receive treatment for their OUD. In the current study, we conducted focus groups with staff working in public mental health outpatient clinics to explore organizational capacity challenges to implementing medication for OUD (MOUD).

**Methods:** We conducted two focus groups (composed of medical/supervisory staff or front-line clinical staff) at 8 publicly funded clinics (n = 108) in Los Angeles County selected for diversity and size. Focus groups were recorded and transcribed. Transcript coding was guided by Meyer et al. (2012)’s eight constructs of organizational capacity for public health.

**Results:** Themes were largely consistent among the clinics. The most frequently mentioned capacity constraints were workforce, system boundaries, inter-organizational relationships, organizational culture, governance/decision-making, physical infrastructure and fiscal/economic. Staff from 7 clinics perceived that they lacked training and expertise. Staff at 7 clinics reported their caseloads were heavy and more specialized staff were needed to address OUD. Staff at 6 clinics reported mixed messages about MOUD from leadership and within the organizational culture. Staff at 7 clinics reported challenges accessing substance use treatment resources in their community. Staff at 6 clinics perceived that few clients had OUD. Staff also described capacity constraints in physical infrastructure, such as lack of office space (3 clinics) or facilities for urine testing and MOUD delivery (5 clinics). Finally, staff at 4 clinics mentioned unclear billing procedures for OUD. Despite these barriers, participants were receptive to receiving more training and resources to implement MOUD.

**Conclusion:** There are significant capacity constraints to implementing MOUD in public mental health clinics, highlighting the need to develop effective implementation strategies to address these gaps.

## A64 “Contingency management tracker: a research tool and implementation strategy in one” (IS14)

### Bryan R. Garner, Elizabeth Ball, Leena Dave, Roger Jesrani, Joseph Nofziger, Alyssa Toro, Samantha Moul, Kimberly Yap, and Sara J. Becker

#### Lead author affiliation: RTI International, 3040 E Cornwallis Rd, Durham, NC 27709, USA

##### **Correspondence:** Bryan R. Garner (bgarner@rti.org)

*Addict Sci Clin Pract* 2020, **15(Suppl 2)**:A64

**Background:** Contingency management (CM) is one of the only behavioral interventions shown to improve patient abstinence from opioids when combined with FDA-approved pharmacotherapy. Unfortunately, the implementation of CM in opioid treatment programs (OTPs) remains low. Additionally, research suggests when CM is implemented in real-world treatment settings, it is implemented with poor adherence. The objective of this poster is to describe the development and implementation of CM Tracker, which was custom developed to serve the dual purpose of a research tool for standardizing the assessment of CM implementation and an implementation strategy (i.e., develop and implement tools for quality monitoring) to improve adherence.

**Methods:** Funded by the National Institute on Drug Abuse, Project MIMIC (Maximizing Implementation of Motivational Incentives in Clinics) is a type 3 hybrid trial testing strategies for helping OTPs and their staff implement CM. Beyond general implementation data, CM Tracker was designed to collect and store data specific to the escalating prize-based CM protocol being implemented as part of Project MIMIC. By providing simple inputs about CM sessions, OTP staff receive a user-friendly dashboard that provides visual information to support CM implementation with patients.

**Results:** To date, 22 CM staff have used the project’s CM Tracker for documenting the quality of CM implementation. Of the project’s 188 patient participants, 162 (86%) initiated CM. Per data collected using CM Tracker, the median number of CM sessions implemented per patient was 7 (out of 12 possible sessions).

**Conclusion:** The CM Tracker is an innovative tool that can simultaneously serve a research tool and an implementation strategy. Future research is needed to examine the extent to which OTPs sustain the implementation of CM, as well as examine the extent to which they develop their own tools for monitoring quality of CM implementation.

## A65 “The role of an intermediary purveyor organization in identifying and responding to workforce priorities to combat the opioid epidemic” (IS15)

### Bryan Hartzler, and Denna Vandersloot

#### Lead author affiliation: Alcohol & Drug Abuse Institute at University of Washington, 1107 NE 45th St, Seattle, WA 98105, USA

##### **Correspondence:** Bryan Hartzler (hartzb@uw.edu)

*Addict Sci Clin Pract* 2020, **15(Suppl 2)**:A65

**Background:** Following identification of technical assistance priorities for the opioid epidemic via a needs assessment survey of workforce members in Health and Human Services Region 10, the Northwest Addiction Technology Transfer Center (Northwest ATTC) embarked on corresponding provision of universal, targeted, and intensive technical assistance. Responsive efforts by this intermediary purveyor organization are ongoing, and include sponsorship of products and activities for local, regional, and national audiences.

**Methods:** Iteratively developed by a multidisciplinary team, survey content included eight items on prevention, treatment, and recovery practices for opioid use disorder (OUD) rated on a five-point scale of importance (1 = Not At All, 5 = Extremely). A lone inclusion criterion for workforce respondents, recruited via the Northwest ATTC website, was employment as a health professional in HHS Region 10. Cumulative record of responsive products and activities over the subsequent 18 months was compiled by Northwest ATTC leadership.

**Results:** In this workforce sample (N = 306), the three greatest priorities were treatment of OUD and co-occurring disorders (M = 4.47, SD = 0.73), clinical services for pregnant/parenting women with OUD (M = 4.39, SD = 0.88), and community-based OUD recovery support (M = 4.32, SD = 0.85). Generalized linear models confirmed this pattern as robust across the four HHS Region 10 states. As for responsivity to these identified priorities, Northwest ATTC has sponsored: (1) expositions and exhibits at state and regional conferences, (2) webinar presentations for regional and national audiences, (3) training workshops for local workforce groups, (4) online educational products, and (5) intensive technical assistance projects to support implementation of useful practices by partnering health agencies.

**Conclusions:** Initial, survey-based needs assessment of a regional addiction workforce informed subsequent Northwest ATTC provision of universal, targeted, and intensive technical assistance to combat the opioid epidemic. This process may be informative to other intermediary purveyor organizations seeking to address workforce priorities in their locales.

## A66 “Training dental residents on opioid use disorder: a national survey” (IS16)

### Shenam Ticku, Tien Jiang, Hesham Alhazmi, Nora Alamer, and Christine Riedy

#### Lead author affiliation: Harvard School of Dental Medicine, 188 Longwood Ave, Boston, MA 02115, USA

##### **Correspondence:** Shenam Ticku (shenam_ticku@hsdm.harvard.edu)

*Addict Sci Clin Pract* 2020, **15(Suppl 2)**:A66

**Background:** Dentists are one of the highest prescribers of opioids. Several dental schools and state dental associations have made efforts to increase curricular offerings on opioid use disorder (OUD) and appropriate prescription of opioids. The purpose of this study was to conduct an environmental scan on the inclusion of OUD in postgraduate dental training.

**Methods:** A 46-item self-administered online survey was sent to 265 dental postgraduate primary care [Advanced Education in General Dentistry (AEGD) and General Practice Residency (GPR)] program directors (PDs) in February 2019. The survey included questions on behavioral health content (BH), e.g. inclusion of OUD, importance of training residents on OUD, evaluation of resident competence and usage of the prescription drug monitoring program (PDMP).

**Results:** We received 111 responses from GPR and AEGD PDs (42% response rate). The majority of the programs think it is important for residents to receive training (n = 97, 87%) in identifying OUD and knowing community and oral health resources to provide referrals (n = 97, 87%). Similarly, a majority (n = 95, 86%) of the respondents train their residents to identify OUD. While only 42% (n = 42) of the respondents evaluate students on BH in their curricula, a majority of those evaluated consider their residents competent in identifying OUD (n = 35, 83%) and knowing resources to provide referrals for the disorder (n = 36, 86%). Around 80% (n = 90) of the programs teach their residents to utilize the state’s PDMP.

**Conclusion:** It is encouraging that most PDs train their residents to identify OUD. Best practices need to be disseminated to increase uptake in programs that have not yet included OUD in their curricula and to utilize the state’s PDMP program. Finally, evaluation of residents’ competency to identify and refer their patients with OUD needs greater importance.

## A67 “Virtualization of training and technical assistance: a rapid response to the needs of the behavioral health workforce due to COVID-19” (IS17)

### Nancy Roget, Sara J. Becker, Michael Chaple, Tom Freese, Heather Gotham, Holly Hagle, Maxine Henry, Igor Koutsenok, Laurie Krom, Rosemarie Martin, Todd Molfenter, Kristen Powell, Laura Saunders, Isa Velez, Ruth Yanez, and Joyce Hartje

#### Lead author affiliation: Center for the Application of Substance Abuse Technologies at University of Nevada, Reno, 1664N Virginia St, Reno, NV 89557, USA

##### **Correspondence:** Nancy Roget (roget@unr.edu)

*Addict Sci Clin Pract* 2020, **15(Suppl 2)**:A67

**Background:** The Substance Abuse and Mental Health Services Administration (SAMHSA) funds three technology transfer center (TTC) networks focused on addiction treatment (ATTC), mental health (MHTTC), and substance use prevention (PTTC). TTCs are charged with developing and strengthening the behavioral health workforce, and helping organizations incorporate effective practices, through free training and technical assistance (T/TA). Each Network is comprised of 10 US-based Centers, a National American Indian and Alaskan Native Center, a National Hispanic and Latino Center, and a Network Coordinating Office. In addition, the ATTC Network includes 6 International HIV Centers (funded by the President’s Emergency Plan For AIDS Relief).

**Methods:** During Spring 2020, the TTCs quickly shifted an entire continuum of T/TA offerings from primarily in-person to virtual delivery since policies implemented to mitigate COVID-19 spread, including stay-at-home orders and social distancing, prohibited in-person attendance. TTCs rapidly innovated to ensure that the workforce had access to virtual T/TA without disruption. Each TTC was surveyed about COVID activities and 41 responded: 13 ATTCs, 12 PTTCs, 10 MHTTCs; and 6 International.

**Results:** Lessons learned in this shift to virtual T/TA service delivery will be highlighted including: using technology (expansion of videoconferencing platforms); promoting best practices for online learning (educating traditionally in-person trainers about virtual learning);creating downloadable products to augment/reinforce learning (fact/tip sheets); sponsoring online events to gather data on T/TA needs (listening sessions); and conducting T/TA specific to using telehealth technologies to deliver SUD and MH services. Ongoing monitoring and evaluation will be needed to assess the extent to which virtual T/TA delivery is associated with comparable provider satisfaction, knowledge, skill, and practice implementation, relative to in-person T/TA.

**Conclusion:** The rapid virtualization (Shore 2020) of T/TA services by the TTCs demonstrates adaptability and carries important implications for designing a hybrid service delivery model post pandemic.

## A68 “A participatory process to develop a naloxone, buprenorphine, and syringe safety intervention for community pharmacies” (MM01)

### Traci C. Green, Jesse Bogis, Adriane Irwin, Jeffrey Bratberg, Mary Gray, Caleb Banta-Green, Ryan Hansen, Anthony Floyd, Gillian Leichtling, and Dan Hartung

#### Lead Author Affiliation: Heller School for Social Policy and Management at Brandeis University, 415 South St, Waltham, MA 02453, USA

##### **Correspondence:** Jesse Bogis (jboggis@brandeis.edu)

*Addict Sci Clin Pract* 2020, **15(Suppl 2)**:A68

**Background:** Pharmacies are well positioned to mitigate opioid risks through provision of naloxone to people taking opioid medications and supplying nonprescription syringes for safe injection. Many pharmacists are unsure of how to address these topics with patients and some may harbor stigma towards people who use drugs (PWUD). We used a participatory design process with multiple stakeholders to integrate two evidence-based opioid safety-focused training toolkits (MOON and RESPOND) to enhance content related to buprenorphine dispensing and nonprescription syringe sales.

**Methods:** MOON materials focused on naloxone knowledge and dispensing; RESPOND emphasized communication strategies and importance of opioid safety screenings. We formed external advisory committees (EACs) across each state comprised of pharmacists, policy makers, community health workers, and researchers (n = 20) to provide feedback via online survey and presentations to local task forces. Three in-person focus groups with PWUD (n = 17) in one urban and two rural areas were held. Toolkits included a continuing education online course, academic detailing, as well as pharmacist-and patient-facing materials.

**Results:** EAC survey responses and task force discussions affirmed the need to focus the online course and academic detailing on naloxone assessment, counseling, and pharmacy workflow. New content and tool enhancement areas pertained to buprenorphine effectiveness, concomitant medication monitoring, prescriber communication; and reducing syringe purchase and sale stigma, community benefit of syringe sales, and importance of safe disposal. Focus group participants discussed toolkit refinements to: (1) reduce stigma, (2) present clear patient-facing messaging with pictures, (3) offer syringe disposal containers, and (4) use training videos with more realistic scenarios.

**Conclusions:** Adaptation and enhancement of a comprehensive, evidence-based toolkit for pharmacists was formalized through a participatory process with multiple stakeholders. Community engagement in intervention development improved the validity and meaning of materials for stakeholders, especially for PWUD.

## A69 “Assessing a facilitation-based implementation strategy using the Grasha–Riechmann framework: results from the substance abuse treatment to HIV care (SAT2HIV) project” (MM02)

### Jay Ford, Aaron Gilson, Martha Maurer, Kim A. Hoffman, and Bryan R. Garner

#### Lead Author Affiliation: Oregon Health & Science University, 3181 SW Sam Jackson Park Rd, Portland, OR 97239, USA

##### **Correspondence:** Kim A. Hoffman (hoffmaki@ohsu.edu)

*Addict Sci Clin Pract* 2020, **15(Suppl 2)**:A69

**Background:** The Implementation & Sustainment Facilitation (ISF) strategy was found to be an effective implementation strategy for improving the integration of a motivational interviewing-based brief intervention for substance use disorders within HIV community-based organizations (CBOs). Using the Grasha–Riechmann framework, which codifies teaching styles into five categories: Delegator, Expert, Facilitator, Formal Authority and Personal Model, we sought to understand the styles used to deliver the ISF strategy. To our knowledge, the Grasha–Riechmann framework has not been applied to a facilitation-based implementation strategy.

**Methods:** The ISF strategy facilitation meetings (n = 137) were recorded and transcribed. Participants included intervention and leadership staff. A deductive, thematic coding framework using the Grasha–Riechmann framework was applied to the transcripts. NVivo qualitative software facilitated coding of text for analysis.

**Results:** Preliminary results show that the Grasha–Riechmann framework is useful for identifying styles of facilitation, as well as the individual elements within those styles. Examples included: Promoting a mechanism or tool that will enable immediate independent work (Delegator); heading off/foreseeing future problems (Expert); offering empathy and/or encouragement (Facilitator); setting expectations (Formal Authority); and providing explicit examples of how the facilitator or others have carried out a task in the past (Personal Model).

**Conclusion:** Results demonstrated the feasibility of using the Grasha–Riechmann framework for coding a facilitation-based implementation strategy. Findings fill a gap in the literature, as few studies to date have examined the techniques employed by facilitators to improve motivational interviewing implementation in HIV-CBOs. A future investigation will employ a mixed-methods approach to compare the qualitative analyses of facilitation transcripts to the fidelity of the ISF strategy and HIV-CBO impact. Triangulating quantitative and qualitative data could provide an opportunity to better understand facilitation-based interventions by using a qualitative approach to validate the quantitative self-report method.

## A70 “Assessing the validity of pharmacy syringe sales data to inform opioid addiction trends and response” (MM03)

### Traci C. Green, Thomas J. Stopka, Ziming Xuan, Tyler C. Davis, Jesse Boggis, Adriane Irwin, Mary Gray, Daniel Hartung, and Jeffrey Bratberg

#### Lead Author Affiliation: Heller School for Social Policy and Management at Brandeis University, 415 South St, Waltham, MA 02453, USA

##### **Correspondence:** Traci C. Green (Tracigreen@brandeis.edu)

*Addict Sci Clin Pract* 2020, **15(Suppl 2)**:A70

**Background:** The role pharmacies play in addressing the opioid crisis and drug-related risks like injection drug use is evolving. Estimating the prevalence of injection drug use at the community level is challenging due to the hidden nature of drug use. Many community pharmacies sell nonprescription syringes, thus pharmacy-level sales of injection equipment may be an indicator of drug-related harm and unmet need from high-risk populations. We aimed to assess the convergent validity of staff-reported syringe sales volumes and syringe sales data from community pharmacies in Massachusetts and Rhode Island.

**Methods:** Between November 2017 and January 2018, we administered a telephone-based survey to estimate average weekly nonprescription syringe sale type and volume for 191 retail chain (CVS) pharmacies located in communities experiencing fatal opioid overdoses above the state’s annual median rate. For the same period, we obtained syringe sales data from surveyed pharmacies and all CVS pharmacies in the two states. CVS Pharmacy is the largest retail pharmacy chain in both study states. We ran Spearman correlations to assess convergence of average weekly volume between pharmacy staff reports and sales data.

**Results:** All pharmacies responded to the survey. Most (98.4%) pharmacies surveyed sold nonprescription syringes but 41.4% reported running out of stock monthly or more frequently. Pharmacy staff tended to under-report syringe sales. Staff reported weekly nonprescription syringe sales volume was 67,922 compared with 70,962 from the administrative pharmacy sales data. Spearman’s correlation between reported and actual syringe sales data was 0.40 (95% CI = 0.27–0.51).

**Conclusions:** Pharmacy syringe sales data can provide a real-time, geographically specific, anonymous data source to track emerging trends and tailor local responses. The counts of administrative pharmacy syringe sales data in Massachusetts and Rhode Island indicate high need, substantial volume, and notable access at community pharmacies.

## A71 “Building bridges: fostering interagency collaboration to combat the opioid overdose crisis with community planning meetings” (MM04)

### Saad T. Siddiqui, Anna La Manna, Liz Connors, Philip Horn, Jeremiah Goulka, Rachel P. Winograd, Leo Beletsky, and Claire A. Wood

#### Lead Author Affiliation: Missouri Institute of Mental Health, 4633 World Pkwy Cir, St. Louis, MO 63134, USA

##### **Correspondence:** saad.siddiqui@mimh.edu

*Addict Sci Clin Pract* 2020, **15(Suppl 2)**:A71

**Background:** Innovative approaches are needed to address the opioid overdose crisis. Connecting the DOTS—Drug Overdose Trust and Safety, a large-scale SAMHSA-funded project, aims to foster collaboration between historically siloed organizations and community stakeholders, provide occupational safety and harm reduction training to first responders, distribute naloxone, and increase the integration of care coordination services within first responder agencies. Planning sessions were conducted in two counties in Missouri to encourage community collaboration and customize training content. The differences in baseline levels of collaboration and partnership factors between two urban counties were assessed.

**Methods:** Participants were surveyed at the beginning of the planning sessions on their demographic characteristics, current levels of collaboration, anticipated benefits from collaborating, partnership factors, as well as open-ended responses to gauge reasons for attending, expectations, and measures of success.

**Results:** Planning sessions were attended by county health officials, law enforcement, fire and EMS representatives, substance use service providers, and members of local recovery organizations. St. Louis City (STL) attendees were more racially diverse (66.7% White, 26.7% Black) compared to Jackson County (JC) [Kansas City Metro Area] 96.7% White, 3.3% Native American). Attendees reported similar levels of collaboration among participating organizations in each county; however, levels of trust between partners were significantly higher in Jackson County than St. Louis City (F(1,55) = 2.57, p = 0.008).

**Conclusion:** Historical and existing collaboration and partnership differences between STL and JC may be a result of contextual and resource-based differences between the counties. Providing a forum to communicate ideas and issues can mitigate uncertainty and skepticism, eventually building relationships, developing trust, and contributing to the partnerships' success. The baseline scores and differences will inform our approach in adapting the training curriculum to context, fostering impactful partnerships towards reducing opioid overdose mortality rates.

## A72 “Change sensitivity and validation of the substance use recovery evaluator (SURE)” (MM05)

### Emma L. Hatton, Peter J. Kelly, Clin Briony Larance, Frank P. Deane, Amanda Baker, and Laura Robinson

#### Lead Author Affiliation: University of Wollongong, Northfields Ave, Wollongong NSW 2522, Australia

##### **Correspondence:** Emma L. Hatton (elh985@uowmail.edu.au)

*Addict Sci Clin Pract* 2020, **15(Suppl 2)**:A72

**Background:** The Substance Use Recovery Evaluator (SURE) is a Patient-Reported Outcome Measure developed with extensive involvement from service users. Our study undertakes a psychometric evaluation of the SURE and examines whether it is sensitive in detecting changes in recovery over time.

**Methods:** A total of 222 participants completed the SURE along psychological distress (Kessler-10), substance use (Timeline Follow-Back) and quality of life (Eurohis-8) measures at baseline during residential treatment, and 3- and 6-months after discharge. Confirmatory factor analyses (CFA), internal consistency, and correlations assessed psychometric validity. To assess change over the follow-up period, statistical as well as clinically significant and reliable change indices were calculated. Correlations between mean changes on SURE, Kessler-10 and Eurohis-8 assessed change sensitivity.

**Results:** The CFA suggested a four-factor structure of all SURE items (χ^2^ = 22.25, RMR = 0.023, GFI = 0.981, NFI = 0.972) except those from the Material Resources subscale which had low internal consistency and low factor loadings. The total SURE scale and remaining 4 subscales had acceptable to excellent internal consistency (α = 0.7–0.9) and significant correlations with quality of life (*r*_*s*_ = 0.73), days of substance use (*r*_*s*_ = − 0.51), and psychological distress (*r*_*s*_ = 74). There was statistically significant change on the total SURE score, and Drug Use, Self-care and Relationships subscales with medium effect sizes. For the total SURE score, 31% of participants demonstrated reliable deterioration or improvement. SURE mean changes were significantly correlated with mean changes on quality of life (*r*_*s*_ = 61) and psychological distress (*r*_*s*_ = 50).

Discussion: The SURE is a valid and reliable measure which shows change sensitivity, indicates reliable changes over time, and correlates well with other validated measures of treatment outcome. Although the Material Resources subscale might have clinical utility, these items are not recommended for assessing change over time or for research purposes.

## A73 “Core elements of family therapy for adolescents in community settings: construct and predictive validity” (MM06)

### Nicole Porter, Aaron Hogue, Molly Bobek, Sarah Dauber, Michael Southam-Gerow, Bryce McLeod, and Craig Henderson

#### Lead Author Affiliation: Center on Addiction, 485 Lexington Ave, New York, NY 10017, USA

##### **Correspondence:** Nicole Porter (nporter@centeronaddiction.org)

*Addict Sci Clin Pract* 2020, **15(Suppl 2)**:A73

**Background:** This work advances efforts to disseminate family-based treatment in routine care for adolescent conduct and substance use problems by examining the construct and predictive validity of core elements of family therapy: Interactional Change, Relational Reframe, Adolescent Engagement, and Relational Emphasis. The core elements were derived from an observational distillation study of high-fidelity family therapy sessions conducted by expert clinicians.

**Methods:** The study sampled recorded sessions and clinical outcome data from 161 cases participating in one of three studies: an implementation trial of Functional Family Therapy (98 sessions/50 cases), an adaptation trial of Multisystemic Therapy (115 sessions/59 cases), and a naturalistic trial of non-manualized family therapy in usual care (107 sessions/52 cases). Adolescents were 60% male with an average age of 15.4 years (SD = 1.7); 15% were African American, 27% White Non-Hispanic, 49% Hispanic American, and 9% other. Session recordings (n = 320) were randomly selected for each case and coded for 21 discrete techniques. Outcome data were collected through 12-month follow-up.

**Results:** Confirmatory factor analyses replicated the factor structure of the family therapy techniques from the original distillation study, confirming four coherent treatment modules: Interactional Change (ICC = 0.77, Cronbach’s α = 0.81); Relational Reframe (ICC = 0.75, α = 0.81); Adolescent Engagement (ICC = 0.72, α = 0.78); Relational Emphasis (ICC = 0.76, α = 0.80). Latent growth curve modeling demonstrated that for each module, greater use of core family techniques predicted improvement in at least one clinical outcome (substance use, externalizing and internalizing problems, family cohesion and conflict); predictive effects were found for multiple outcomes reported by multiple sources.

**Conclusion:** Core elements of empirically supported family therapy models for adolescent behavior problems are clinically viable when delivered in routine care and demonstrate direct links between extensiveness of core module delivery and long-term client gains.

## A74 “Effects & implementation of state-level naloxone pharmacy access laws: an online Delphi process” (MM07)

### Sean Grant, and Rosanna Smart

#### Lead Author Affiliation: Richard M. Fairbanks School of Public Health at Indiana University, 1050 Wishard Blvd, Indianapolis, IN 46202, USA

##### **Correspondence:** Sean Grant (spgrant@iu.edu)

*Addict Sci Clin Pract* 2020, **15(Suppl 2)**:A74

**Background:** Recent state legislation has sought to expand naloxone pharmacy distribution in order to reduce opioid-related harms. This study aimed to examine experts’ views on various state-level naloxone pharmacy access policies.

**Methods:** We recruited a purposive sample of 46 key stakeholders (advocates, healthcare providers, human/social service practitioners, policymakers, and researchers) with experience and expertise in naloxone pharmacy access policies to participate in an online Delphi process. We provided participants with a list of 15 state-level policies: five targeting naloxone prescribers/prescription, six targeting naloxone dispensers/distribution, and four targeting patients/individuals obtaining naloxone. In Round One, stakeholders in Panel A (n = 24) rated the average effect of each policy (assuming it had been implemented as intended) on naloxone pharmacy distribution, opioid use disorder prevalence (OUD), nonfatal opioid overdoses, and opioid overdose mortality. Stakeholders in Panel B (n = 22) rated the acceptability, feasibility, affordability, and equitability of each policy. In Round Two, participants reviewed the Round One results and engaged in an anonymous, moderated, online discussion with other participants. In Round Three, participants revised their Round One responses in light of Round Two. We examined consensus on the effects of opioid-related outcomes (Panel A) and on implementation-related considerations (Panel B).

**Results:** Experts rated three policies to yield a decrease on fatal overdose: Statewide Standing/Protocol Order, Over-the-Counter Pharmacy Supply, and Statewide “Free Naloxone”. Of these, experts rated only Statewide Standing/Protocol Order as high on all implementation criteria (i.e., high acceptability, feasibility, affordability, and equitability). Experts perceived liability protections and required provision of education or training as having little-to-no effect on naloxone distribution. All policies had little-to-no change on prevalence of OUD and nonfatal overdose. All Naloxone Dispenser/Distribution policies had high acceptability, while all Naloxone Prescriber/Prescription policies had little-to-no change on fatal overdose. While only five policies (33%) have “high” equitability, no policy has “low” acceptability, feasibility, affordability, or equitability.

**Conclusion:** The results of this study will help researchers better characterize naloxone policies and guide policymakers in making decisions about naloxone access. Analyses will inform an evidence-to-decision framework for policymakers in this area and will be used in future empirical work characterizing state-level opioid ecosystems.

## A75 “Examining preliminary data for opioid-related technical assistance requests for underserved communities” (MM08)

### Holly Hagle, Michael Knabel, Yifei Liu, Frances Bozsik, Ignacio Alex Barajas Munoz, Laurie Krom, Aimee Campbell, Kathryn Cates-Wessel, and Frances Levin

#### Lead Author Affiliation: University of Missouri, Kansas City, 5000 Holmes St, Kansas City, MO 64110, USA

##### Correspondence: Holly Hagle (hagleh@umkc.edu)

*Addict Sci Clin Pract* 2020, **15(Suppl 2)**:A75

**Background:** Funded by the Substance Abuse and Mental Health Services Administration (SAMHSA) and led by the American Academy of Addiction Psychiatry (AAAP), in collaboration with the University of Missouri, Kansas City, Addiction Technology Transfer Center (ATTC) and Columbia University Division of Substance Use Disorder, a large coalition of national professional organizations created the Opioid Response Network (ORN) which launched May, 2018. Through an individualized intake session, the TA requester indicates if the TA request involves a unique or hard-to-reach population, such as justice-involved populations, people experiencing housing instability, people who are uninsured or underinsured, and Black, Indigenous, people of color (BIPoC) communities.

**Methods:** The ORN project team is conducting qualitative analysis to describe the TA type, levels (Basic TA, Targeted TA, and Intensive TA) and types of TA activities. Data analysis includes using consensus methods to synthesize the unique and hard-to-reach TA requests and nominal group technique. Senior researchers lead the group in identifying key terms for coding, iterative coding, and review through consensus sessions.

**Results:** The ORN TA project identified 21 unique and hard-to-reach populations as part of the TA request process (44% of all TA requests). Twenty key terms were identified. Subsequent rounds of coding and code reviews are taking place, which includes reviews of coded data, thematic analysis, and quantitative descriptive analysis of project data.

**Conclusion:** Preliminary results indicate early identification as part of a systematic process to identify unique and hard-to-reach populations may facilitate tailored TA. Individualized support can be given to the TA requester to identify unique and hard-to-reach populations. Further analysis is necessary to identify impact and outcomes on TA delivery across diverse populations.

## A76 “Parent engagement in treatment for adolescent behavior problems: construct and predictive validity” (MM09)

### Nicole Porter, Aaron Hogue, Molly Bobek, and Craig Henderson

#### Lead Author Affiliation: Center on Addiction, 485 Lexington Ave, New York, NY 10017, USA

##### **Correspondence:** Nicole Porter (nporter@centeronaddiction.org)

*Addict Sci Clin Pract* 2020, **15(Suppl 2)**:A76

**Background:** Family therapy (FT) has the strongest evidence base for treating conduct and substance use disorders in adolescents. A fundamental and unique feature of FT models for adolescent externalizing problems that differentiates FT from other approaches is family engagement. Family engagement is characterized by interventions aimed to enhance family members’ involvement in therapy and promote investment in the therapy process. The current study leveraged three existing manualized FT models to identify and distill core parent engagement (PE) techniques that are conceptually and clinically distinct from other FT interventions. Then, using a front-line sample to maximize generalizability, this study tested the construct and predictive validity of a PE factor, derived from FT theory and clinical practice.

**Methods:** Sessions were sampled from tapes collected from community clinicians treating adolescents with substance use and co-occurring behavioral problems in usual care. Sessions were observationally coded for the presence of 4 PE techniques: Enhances Love and Commitment, Instills Hope, Parent Ecosystem, and Joins with Parents.

**Results:** Descriptive statistics for the PE factor demonstrate strong internal consistency (4 techniques; α = 0.71) suggesting a reliable and valid construct. Modest correlation with other FT Factors (i.e., Interactional Change, Relational Reframe, Adolescent Engagement, Relational Emphasis; Hogue et al. 2020) suggests differentiation from other core FT techniques. Latent growth curve modeling was used to examine technique-outcome associations over 12-month follow-up. PE score was included as a predictor, controlling for FT model, therapist effects, adolescent ethnicity, sex, and age. Results suggest more extensive use of PE techniques was associated with significant decrease in adolescent substance use. Counter to study hypotheses, use of PE techniques was associated with significant increases in internalizing and externalizing symptoms.

**Conclusion:** Frontline therapists employing family-based interventions intensified their use of PE techniques for those youth who behavioral symptoms showed greatest decline over time.

## A77 “Patterns of opioid use in commercially insured cancer patients” (MM10)

### Sean P. Fleming, Tham T. Le, and Linda Simoni-Wastila

#### Lead Author Affiliation: University of Maryland Baltimore School of Pharmacy, 20N Pine St, Baltimore, MD 21201, USA

##### **Correspondence:** Sean P. Fleming (sean.fleming@umaryland.edu)

*Addict Sci Clin Pract* 2020, **15(Suppl 2)**:A77

**Background:** In the era of heightened opioid prescribing scrutiny, little research assesses opioid se in cancer patients. We examined patterns of and factors associated with opioid utilization in newly diagnosed cancer patients.

**Methods:** A retrospective cohort study of individuals aged 18 to 64 years with a new cancer diagnosis in IQVIA PharMetrics® Plus (1/1/2007–12/31/2013). Study participants were continuously enrolled 12 months pre- and 24 months post-cancer diagnosis. Opioid prevalence, total days supplied, number of prescriptions, and Morphine Equivalent Daily Dose (MEDD) were measured in the 2-years following cancer diagnosis. Multivariable logistic regression models identified factors associated with odds of receiving opioids post-cancer diagnosis.

**Results:** Of 191,616 eligible individuals, 93,739 (48.9%) received an opioid after cancer diagnosis; of these, 56,025 (59.8%) were new users. In the 2-years following cancer diagnosis, opioid users received a mean 4.6 prescriptions covering 65 total days supply with a mean MEDD of 31.8 mg. Only 2387 (2.5%) patients had high MEDD (≥ 90 mg). Baseline predictors of opioid use after cancer diagnosis included secondary cancer [OR 2.47, 95% CI 2.07–2.96], baseline opioid use [2.46 (2.40–2.51)], muscle relaxant use [1.64 (1.57–1.71)], and specific cancer sites including breast [2.0 (1.75–2.29)], gynecological [1.77 (1.54–2.04)], and head and neck [1.51 (1.32–1.71)].

**Conclusions:** We found no evidence of excessive opioid use following new cancer diagnoses. Cancer-related factors, including site and progression to secondary cancer, were among the strongest predictors of opioid use in the cancer population. Current policies to reduce opioid overuse and adverse events may unintentionally impact access to opioid medications used to treat cancer-related pain. In order to avoid under-treatment of cancer pain, patient-centered policies and prescribing practices should be adopted to minimize unintended consequences of broad opioid policies intended to limit inappropriate opioid use.

## A78 “Pharmacy-based care model for the treatment of opioid use disorder: pilot findings and novel care adaptations during COVID-19” (MM11)

### Traci C. Green, Michelle McKenzie, Rachel Serafinski, Seth Clark, Kirsten Langdon, Josiah Rich, and Jeffrey Bratberg

#### Lead Author Affiliation: Heller School for Social Policy and Management at Brandeis University, 415 South St, Waltham, MA 02453, USA

##### **Correspondence:** Traci C. Green (traci.c.green@gmail.com)

*Addict Sci Clin Pract* 2020, **15(Suppl 2)**:A78

**Background:** Buprenorphine/naloxone (BNX) and naltrexone (NTX) are critical components of addressing the current opioid epidemic, yet treatment need vastly exceeds treatment availability. In Rhode Island, one new model of MAT provision considered the expansion of BNX and NTX care into pharmacies through a collaborative pharmacy practice agreement (CPA). The current study aimed to determine the feasibility of providing pharmacy-based MAT care to 11 patients with opioid use disorder, and to consider adaptations of this model during COVID-19.

**Methods:** A CPA for MAT was developed by state, community pharmacy, and study team members, drawing from existing MAT models of nurse case manager office-based care and adapting extant pharmacy-MAT models for community pharmacies. Once approved by state officials, we trained 17 pharmacists in MAT care provision principles over a 20-h online and in-person course designed in partnership with national organizations for the study. We then piloted the CPA with 11 patients receiving BNX maintenance doses who visited the study pharmacy at least weekly for 1 month. All toxicological testing was oral and observed; pharmacy care notes were provided to the collaborating prescriber within 8 h of visits. Study assessments were in-person and included self-reported behavioral measures of drug use, safety, and social and health stability and clinical measures such as drug toxicology, counseling, and clinic visit attendance. Feasibility was assessed from patients as well as from pharmacists delivering the intervention through a self-reported Likert-scale item. During the COVID-19 crisis, state and community concerns for broader MAT care brought about further CPA adaptations.

**Results:** Eleven patients (5 women, 6 men, 40% non-white race) aged 23 to 60 years completed 70 clinic visits for buprenorphine care at two locations. There were no adverse events and patients with mandated counseling and other requirements continued in their receipt. Pilot participants were safely transitioned to and from the pharmacy. All pharmacists rated the CPA model highly feasible; patients similarly rated the care receipt highly. Patients noted the efficient care, flexibility, family-friendly setting, and low perceived stigma of the pharmacy experience. During COVID-19, changes in permissions from DEA/SAMHSA led to expanding the CPA to support withdrawal care management and pharmacy-facilitated induction.

**Conclusion:** Findings suggest that a CPA care model is feasible and safe for patients on MAT and pharmacists. The CPA model for MAT can further engage pharmacists as part of the patient care team to meet the dynamic needs of patients including during the COVID-19 crisis.

## A79 “Prenatal opioid use disorder in rural communities: measure of providers’ perceptions and attitudes toward treating patients with prenatal opioid use disorder” (MM12)

### Jacob Baylis, Marcela C. Smid, Bhanu Muniyappa, Melissa Cheng, Katherine T. Fortenberry, Danielle Pendergrass, Jacob Foringer, Shayla Archer, Mitchell Garets, Jade Hill, M. Aryana Bryan, Kristi Carlston, Maggie Cooper, and Gerald Cochran

#### Lead Author Affiliation: University of Utah, 201 Presidents' Cir, Salt Lake City, UT 84112, USA

##### **Correspondence:** Jacob Baylis (Jacob.Baylis@hsc.utah.edu)

*Addict Sci Clin Pract* 2020, **15(Suppl 2)**:A79

**Background:** Pregnant women in rural communities are disproportionately affected by opioid use and have limited access to treatment. Little is known about rural provider perceptions/attitudes when treating pregnant patients with opioid use disorder (OUD).

**Objective:** To evaluate health care provider perceptions/attitudes on providing care to patients with prenatal OUD (POUD).

**Methods:** Design. This study was a cross-sectional, one-time close-ended/self-report electronic survey. Setting. The survey was distributed within two high opioid prescribing/overdose counties in rural Utah. All health/behavioral health care providers listed in Utah’s professional license database in the targeted counties were contacted. Assessments. We adapted the Shortened Alcohol and Alcohol Problems Perception Questionnaire to assess provider perceptions/attitudes towards POUD. Analyses. We employed descriptive statistics to characterize the sample; ANOVA and unadjusted/multivariable linear regression tests analyzed provider perceptions/attitudes by key indicators.

**Results:** A total of 225 providers consented to participate in the survey (response rate = 52%). Following screening, 82 providers indicated they provide care for pregnant women with opioid use disorder. Participants included nurses/nurse practitioners/nurse midwifes (54.7%), counselors/therapists/social workers (34.0%), physicians (5.7%), and physician assistants (5.7%). The provider perception/attitude score ranged from 10 to 70 (10 = negative; 70 = positive). A significant difference in mean scores was found between professions (overall mean = 52.1, SD = 9.8, p = 0.04) with nurses having the lowest score (mean = 47.8, SD = 7.1) and nurse practitioners with the highest score (mean = 59.9, SD = 8.4). Adjusted linear regression analyses (adjusted for demographics) showed more hours of prenatal opioid education training were associated with lower perception/attitude scores (β = 2.63, 95% CI [0.72, 4.54], p < 0.01) and providers who feel more competent in asking pregnant patients about opioid use were associated with lower perception/attitude scores (β = 4.81, 95% CI [2.00, 7.62], p < 0.01).

**Conclusions:** Additional education may benefit rural provider’s perceptions/attitudes in providing POUD care. Future research should design/test interventions to improve rural POUD care.

## A80 “Primary care medical staff attitudes toward substance use: results of the substance abuse attitude survey” (MM13)

### Leah Hamilton, Noa Appleton, Sarah Wakeman, Timothy Wilens, Joseph Kannry, Richard N. Rosenthal, Keith Goldfeld, Angeline Adam, Sarah Farkas, Carmen Rosa, John Rotrosen, and Jennifer McNeely

#### Lead Author Affiliation: New York University Grossman School of Medicine, 550 1st Avenue, New York, NY 10016, USA

##### **Correspondence:** Leah Hamilton (leah.hamilton@nyulangone.org)

*Addict Sci Clin Pract* 2020, **15(Suppl 2)**:A80

**Background:** Under-treatment of drug and alcohol use in primary care settings has been attributed, in part, to medical providers’ negative attitudes toward substance use. As a part of an implementation study of EHR-integrated substance use screening in primary care clinics, conducted in the NIDA Clinical Trials Network, we assessed baseline attitudes among medical staff.

**Methods:** Eligible participants were primary care providers and medical assistants in six urban academic primary care clinics. Prior to implementation of a substance use screening program, participants completed the Substance Abuse Attitudes Survey (SAAS), a validated 50-item self-administered survey that measures attitudes to substance use in five domains: permissiveness, non-moralism, non-stereotyping, treatment intervention, and treatment optimism. Participants were asked to rate their level of agreement with each item on a five-point Likert scale.

**Results:** In total, 139/191 (73% response rate) eligible staff completed the survey. Participants were age m = 42; 75% female; 10% Hispanic/Latino, 65% White, 6% Black, 25% Asian (multi-race selection allowed). The sample comprised 78% physicians, 9% nurse practitioners, and 11% medical assistants with an overall average of 13.4 years in practice. Approximately one-third reported moderate to high satisfaction treating patients with drug problems (37.3%) and alcohol problems (36.7%). The proportion of participants having positive attitudes in each of the following domains were: non-moralism (70.1%); non-stereotyping (58.3%); treatment intervention (48.6%); treatment optimism (49.6%); and permissiveness (46.2%).

**Conclusions:** While most primary care staff did not endorse moralistic or stereotyping statements about alcohol and drug use, attitudes toward addiction treatment were mixed, with less than half endorsing positive attitudes toward treatment effectiveness. Our results suggest a need to improve attitudes, particularly toward addiction treatment. This could be accomplished through education and increased exposure to effective interventions that can be delivered by primary care providers, including office-based treatment for alcohol and opioid use disorder.

## A81 “Provider perspectives on integration of substance use disorder and HIV care in Vietnam” (MM14)

### Andrew Edsall, Thanh Thuy Thi Dinh, Pham Phuong Mai, Kim A. Hoffman, Hang T. Nguyen, Tong Thi Khuyen, Nguyen Thu Trang, Gavin Bart, Le Minh Giang, and P. Todd Korthuis

#### Lead Author Affiliation: Oregon Health & Science University School of Medicine, 3181 SW Sam Jackson Park Rd, Portland, OR 97239, USA

##### **Correspondence:** Andrew Edsall (edsalla@ohsu.edu)

*Addict Sci Clin Pract* 2020, **15(Suppl 2)**:A81

**Background:** UNAIDS recommends integration of medications for substance use disorders (SUD) with HIV care to improve HIV outcomes. Yet, integration of HIV and SUD services remains limited in many countries. The objective of this study was to assess provider perceptions of care integration in Vietnam.

**Methods:** Qualitative interviews were conducted with 43 healthcare providers (nurses, physicians, counsellors, pharmacists, and clinic managers) in 8 HIV clinics in northern Vietnam, 2013–2015. Interviews, each of which lasted between 30 and 60 min, were digitally audio-recorded with the consent of the participants. Thematic analysis with a mixed deductive and inductive approach at the semantic level was employed to analyze key topics.

**Results:** Five themes were identified from providers’ attitudes regarding integration of HIV and SUD treatment: (1) integration of treatment for alcohol use disorder is often neglected compared to other SUD treatment; (2) structural challenges must be addressed to increase integration feasibility; (3) integrated care must address workforce limitations; (4) integration must overcome societal and healthcare stigmatization of SUD; and, (5) providers must resolve conflicting views to overcome integration challenges.

**Conclusion:** The experience of providers in Vietnam may be useful to other countries attempting to integrate HIV and SUD services. This study provides critical insights from the perspective of healthcare providers for integrating HIV and SUD treatment in Vietnam, which could inform scale-up of integrated HIV and SUD services in other countries.

## A82 “Sensitivity of paid insurance claims data for identifying hospital patients with opioid use disorder” (MM15)

### Jennifer McNeely, Elizabeth Owens, Emmeline Bone, Noa Appleton, Jasmine Fernando, Scarlett Wang, Johanna Dolle, Roopa Kalyanaraman Marcello, John Billings, and Shane Gallagher

#### Lead Author Affiliation: New York University Grossman School of Medicine, 550 1st Avenue, New York, NY 10016, USA

##### **Correspondence:** Jennifer McNeely (jennifer.mcneely@nyulangone.org)

*Addict Sci Clin Pract* 2020, **15(Suppl 2)**:A82

**Background:** Behavioral health diagnoses are frequently underreported in administrative health data. As part of a pragmatic trial of a hospital addiction consult program, we sought to determine the sensitivity of New York State Medicaid paid claims data for identifying hospital inpatient admissions with opioid use disorder (OUD) in six New York City public hospitals.

**Methods:** We conducted a structured chart review of electronic health records to identify patients with OUD at the time of their hospitalization. Cases selected for review were 2017 admissions to medical/surgical inpatient units of adults who received methadone or buprenorphine in the hospital. The target sample was 100 cases per hospital. Clinicians with addiction medicine expertise reviewed medical charts to determine if the patient had a clinical presentation consistent with OUD during the hospitalization. For those with OUD, we searched for the same hospitalization in NY State Medicaid paid claims data using demographics, hospital identifiers, and admission dates, and examined all ICD-10 discharge diagnoses associated with the admission for codes including OUD/opioid poisoning. Sensitivity was calculated based on cases that were found in the claims data; because our concern was for underreporting of OUD, specificity was not explored.

**Results:** A total of 591 cases were reviewed; 552 (93%) were clinically consistent with OUD. 465 (84%) of the OUD cases were found in the paid claims data, of which 418 (90%) had a discharge diagnosis of OUD or opioid poisoning. There was variation between hospitals, with the rate of capture ranging from 83 to 97%.

**Conclusion:** For hospitalized patients receiving OUD medications, paid public insurance claims appear to have good sensitivity for capturing opioid-related diagnoses. Claims data may be a valuable source of information about treatment and outcomes of this population.

## A83 “Social network dynamics & DUI: a multilevel dyadic analysis of relationship retention, drinking, and support following a first DUI” (MM16)

### Mauri Matsuda, David Kennedy, and Karen Chan Osilla

#### Lead Author Affiliation: Portland State University, 1825 SW Broadway, Portland, OR 97201, USA

##### **Correspondence:** Mauri Matsuda (mmatsuda@pdx.edu)

*Addict Sci Clin Pract* 2020, **15(Suppl 2)**:A83

**Background:** Recent research shows that individuals with networks comprised of greater proportions of drinking partners are more likely to experience risk factors for DUI; furthermore, reductions in network proportions of drinking partners following a DUI is associated with improved drinking outcomes. The present study builds on this prior work by exploring changes in individual’s network members following a DUI through a multilevel lens.

**Methods:** We collected ego-centric social network data on network members in the 2 weeks prior to DUI incident and at completion of the DUI treatment program for a sample of participants (n = 94) enrolled in a larger randomized controlled trial comparing the effects of cognitive behavioral therapy with usual care. We employed multilevel modeling to examine the associations between participant and network member characteristics and network member retention, drinking, and support at followup.

**Results:** Our results indicate that participants were significantly more likely to retain network members with whom they drank and received tangible support from and significantly less likely to retain network members with whom they drank more alcohol than they wanted. Participants were more likely to receive support for reducing drinking from those with whom they drank, those who provided emotional support, and those who provided DUI-specific support.

**Conclusion:** Although prior research shows that having a greater proportion of drinking partners in ones network is associated with risk for DUI, drinking partners are significantly more likely to be retained in networks following a DUI incident. Despite these potential risks, individuals with a first-time DUI were more likely to receive support for reduced drinking from those with whom they drink. The results of this work highlight the importance of multiplexity—an overlap in functions of network members—in research on the social context of drinking.

## A84 “The cannabis retail environment for young adults in Los Angeles: which metrics matter” (MM17)

### Caislin L. Firth, Rachana Seelam, Anthony Rodriguez, Regina Shih, Joan Tucker, Elizabeth D’Amico, and Eric Pedersen

#### Lead Author Affiliation: RAND Corporation, 1776 Main St. Santa Monica, CA 90401-3208, USA

##### **Correspondence:** Caislin L. Firth (caislinleah@gmail.com)

*Addict Sci Clin Pract* 2020, **15(Suppl 2)**:A84

**Background:** Currently, there is no consensus on how to measure cannabis retailer density. Researchers and policy makers need clear measures to support policies that mitigate unintended harms of cannabis legalization. To address this gap, we developed cannabis retailer density metrics and assessed whether they were associated with young adult cannabis use in Los Angeles County (LA), California.

**Methods:** Drawing from GIS-based measures of alcohol outlet density, we developed a series of cannabis retailer density metrics: proximity, counts within 5- 10- 15-, and 30-min driving distances, and considered retail licensure. Retailer addresses were compiled by webscraping cannabis registries (e.g., Weedmaps) and conducting field visits (March 2019). Home addresses were geocoded for young adults who completed a 2019 survey (no. 1097); retailer metrics were calculated for each participant. We fit a series of multi-level logistic regression models to assess which retailer metrics were associated with any past month cannabis use. Models included a random intercept by census tract (CT) and adjusted for age, gender, race/ethnicity, college student, and CT median household income.

**Results:** Thirty percent of young adults used cannabis in the past month, 39% had a retailer within a mile from home, and an average of 14 retailers within a 10-min drive. Licensed retailers were less prevalent; nearest licensed retailer was on average 2.4 miles from home. The odds of past month cannabis use significantly increased by 3% (OR: 1.03, 95% CI 1.00–1.07) for every additional licensed retailer within a 10-min drive in adjusted model; use was also significantly associated with licensed retailers within a 30-min drive (OR: 1.01, 95% CI 1.00–1.01). Other metrics were not significantly associated with past month cannabis use.

**Conclusion:** Findings indicate density metrics may be important indicators for risk among young adults. Such findings can be used to inform policy, prevention, and intervention efforts.

## A85 “The influence of an opioid use disorder on initiating physical therapy for low back pain: a retrospective cohort” (MM18)

### Jake Magel, Adam Gordon, Julie Fritz, and Jaewhan Kim

#### Lead Author Affiliation: University of Utah, 201 Presidents' Cir, Salt Lake City, UT 84112, USA

##### **Correspondence:** Jake Magel (jake.magel@hsc.utah.edu)

*Addict Sci Clin Pract* 2020, **15(Suppl 2)**:A85

**Background:** Low back pain (LBP) is common among patients with an opioid use disorder (OUD). Primary care providers (PCPs) are often the first health care providers consulted when patients seek care for LBP. After this initial encounter, patients are frequently managed in physical therapy. The extent to which patients with an OUD initiate physical therapy for LBP is unknown. To examine the association between a history of an OUD and initiation of physical therapy for LBP within 60 days of a PCP visit for this condition.

**Methods:** Claims from a single state-wide all payer claims database from June 30, 2013 and August 31, 2015 were used to establish a retrospective cohort of patients who consulted a PCP for a new episode of LBP. The outcome measure was the presence of at least 1 physical therapy claim within 60-days after the PCP visit. After propensity score matching on covariates (age, sex, prior history of LBP, comorbid neck pain, obesity, chronic pain, mental health comorbidity, pain medications, residence, insurance carrier and high deductible health plan), logistic regression was used to compare the outcome between patients with a history of an OUD to patients without an OUD.

**Results:** Propensity score matching resulted in 1360 matched pairs of participants with a mean age of 47.2 years (15.9) and 55.9% were female. Compared to patients without an OUD, patients with an OUD were less likely to initiate physical therapy for LBP (aOR = 0.65, 95% CI 0.49 to 0.85).

**Conclusions:** After a visit to a PCP for a new episode of care for LBP, patients with a history of an OUD are less likely to initiate physical therapy than those without an OUD. PCPs may find the results of this study useful when considering the referral of patients with LBP for rehabilitation who have a comorbid OUD.

## A86 “Understanding barriers faced by peer recovery coaches (PRCs) during COVID-19” (MM19)

### Jessie Spencer, Christopher (CJ) Seitz-Brown, Jessica Magidson, Megan Mulheron, Valerie Bradley, and Julia W. Felton

#### Lead Author Affiliation: Michigan State University, 426 Auditorium Road East Lansing, MI 48824, USA

##### **Correspondence:** Jessie Spencer (spenc369@msu.edu)

*Addict Sci Clin Pract* 2020, **15(Suppl 2)**:A86

**Background:** Peer Recovery Coaches (PRC), individuals with lived experience in substance use disorder (SUD), are uniquely suited to overcome barriers low-income clients face during treatment. The COVID-19pandemic forced many SUD treatment facilities to implement new safety and treatment protocols (e.g., telehealth procedures) that may not fit the needs of the population they serve. We aimed to conduct interviews with certified PRCs, peer supervisors, and treatment program staff to understand any new barriers and challenges to delivering SUD services that emerged as a result of COVID-19.

**Methods:** We conducted 15 interviews with PRCs in Detroit, Baltimore, and Washington D.C. Participants were asked about working with clients during stay-at-home orders, whether their training prepared them for delivering services remotely, and barriers associated with COVID-19 treatment protocols and telehealth.

**Results:** The sample was diverse and reflected the communities they served (75% identified as black and 25% as white). On average, participants were certified PRCs for 4 years and worked in a variety of treatment settings (community organizations, emergency departments, residential treatment centers). Peers noted several barriers to assisting clients using telehealth: (1) a drop in the number of patients due to lack of technology, cell-phone minutes, and/or data; (2) a loss of “meetings after the meeting,” which provides peer and group connectedness through physical touch and proximity after group sessions; (3) a feeling that training had not prepared them to work in this context, focusing on “human to human contact” rather than skills needed for engaging clients virtually; and (4) difficulty delivering telehealth services while experiencing their own COVID-19-related anxiety and PTSD.

**Conclusion:** Findings suggest peers perceived significant barriers to transitioning to remote services. Results indicate a need for future research on remote SUD treatment delivery and specific training needs for engaging clients virtually.

## A87 “Understanding the impacts of COVID-19 on substance use among individuals living with HIV: perspectives of HIV service organizations across the United States” (MM20)

### Bryan R. Garner, Brittany A. Zulkiewicz, and Hannah K. Knudsen

#### Lead Author Affiliation: RTI International, 3040 E Cornwallis Rd, Durham, NC 27709, USA

##### **Correspondence:** Bryan R. Garner (bgarner@rti.org)

*Addict Sci Clin Pract* 2020, **15(Suppl 2)**:A87

**Background:** Given the major societal disruptions of the COVID-19 pandemic and its accompanying stressors, concerns are growing that the pandemic is increasing substance use, particularly among individuals with chronic conditions, such as HIV. This study aimed to understand the perceived impacts of COVID-19 on substance use among clients served by HIV service organizations (HSOs) and whether those impacts also increase the need for substance use disorder (SUD) treatment integration in these organizations.

**Methods:** In April 2020, key staff members from 253 HSOs across the US completed an online survey focused on the HSO’s capacity to provide substance use treatment services to clients with SUDs. Survey items measured perceptions of the impact of the COVID-19 pandemic on: (a) their clients’ substance use and (b) increasing the need for the HSO to offer substance use treatment services to clients with SUD, using a scale from 0 representing ‘Not at all’ to 3 representing ‘To a great extent.’

**Results:** Overall, 57% (n = 143) of HSOs reported providing substance use services as part of their service offerings. The average perceived impacts of COVID-19 on clients’ substance use was 2.3 (SD = 0.7), was 2.2 (SD = 0.7) for increasing the prevalence of SUDs among the HSO’s clients, and was 2.1 (SD = 0.8) for increasing the need for the HSO offer effective substance use treatment interventions. HSOs currently lacking substance use services reported lower perceived need to offer effective treatment interventions because of COVID-19 (mean = 2.0, SD = 0.9) than HSOs that currently offer substance use services (mean = 2.3, SD = 0.7, p = 0.002).

**Conclusions:** From the perspective of HSOs, COVID-19 is likely to increase substance use and the prevalence of SUDs among clients served by their organizations. Increases in the prevalence of SUDs due to COVID-19 raise the urgency of integrating SUD interventions into HSOs across the US.

## A88 “Acute healthcare utilization and polysubstance use among people living with HIV and substance use disorder” (SW01)

### Esperanza Romero Rodriguez, Theresa Kim, Christine Lloyd-Travaglini, Michael Winter, Timothy Heeren, Nicolas Bertholet, Alexander Walley, and Richard Saitz

#### Lead Author Affiliation: Boston University, 72 E Concord St, Boston, MA 02118, USA

##### **Correspondence:** Esperanza Romero Rodriguez (espe_mrr@hotmail.com)

*Addict Sci Clin Pract* 2020, **15(Suppl 2)**:A88

**Background:** People living with HIV (PLWH) and substance use disorder (SUD) frequently use acute care services but how use of multiple substances relates to utilization has not been well-described. To determine whether patterns of substance use are associated with acute healthcare utilization among PLWH with SUD.

**Methods:** Participants were recruited from two urban HIV primary care clinics. Inclusion criteria were: (1) HIV infection in medical records, (2) Current DSM-IV substance dependence, or ever injection drug use. Acute healthcare utilization was defined as any past 3-month emergency department visit or hospitalization. Based on latent class analysis, the substance use patterns were: (1) mostly cannabis and unhealthy alcohol use; (2) polysubstance (opioids, cannabis, tranquilizers, cocaine, and unhealthy alcohol use); (3) no drug or unhealthy alcohol use. Past 30-day drug use was assessed using the Addiction Severity Index. Unhealthy alcohol use was assessed with Alcohol Use Disorders Identification Test-Consumption (AUDIT-C). Analysis: generalized estimating equations from repeated measures at baseline, 12-, and 24-month follow-up, adjusting for sociodemographic variables.

**Results:** Among 250 participants, mean ± SD age was 49 ± 9 years, 63% were male, 30% Hispanic, 71% had HIV viral load < 200 copies/mL; 157 (62.8%) were categorized as cannabis and unhealthy alcohol use; 49 (19.6%) as polysubstance use; and 44 (17.6%) as no drug or unhealthy alcohol use; 46% reported acute healthcare utilization. Although there was a trend (acute healthcare utilization 34%, 47%, 53%, respectively; unadjusted p = 0.07), after adjustment there were no significant associations, compared to no substance use, between (1) cannabis and unhealthy alcohol use [adjusted odds ratio (aOR) = 1.17 (95% confidence interval (CI) 0.75, 1.82)], or (2) polysubstance use [aOR = 1.40 (95% CI 0.81, 2.40)], and acute healthcare utilization.

**Conclusion:** Among PLWH with SUD, we did not detect an association between substance use patterns and acute healthcare utilization. Future studies should assess longitudinal effects in larger samples.

## A89 “Association between child welfare policies pertaining to maternal substance use and infant mortality in the United States” (SW02)

### Jad E. Hilal, Debra Bogen, Liz Krans, Lisa Bodnar, Bradley Stein, and Marian Jarlenski

#### Lead Author Affiliation: University of Pittsburgh School of Medicine, 3550 Terrace St, Pittsburgh, PA 15213, USA

##### **Correspondence:** Jad E. Hilal (JEH163@pitt.edu)

*Addict Sci Clin Pract* 2020, **15(Suppl 2)**:A89

**Background:** U.S. states have adopted varying policies that shape how child welfare agencies address maternal substance use. This study examined the association between state child welfare policies pertaining to maternal substance use and infant mortality due to external causes, sudden infant death syndrome (SIDS), and all causes.

**Methods:** Time-series analysis of state child welfare policies pertaining to maternal substance use and state infant mortality rates in all U.S. states from 2000 to 2017. Conditional state fixed-effects negative binomial regression models were fit to determine the adjusted association between such policies and infant mortality rates. Exposures were state adoption of any of 3 types of child welfare laws: requiring healthcare provides to report maternal substance use to child welfare agencies; requiring a plan of safe care for children affected by in utero substance exposure; or defining maternal substance use as child neglect/abuse. Data sources used included the state infant external cause, SIDS, and all-cause mortality rates.

**Results:** During the study time period, 48 states had a mandatory reporting policy, eight had a plan of safe care policy, and 17 had a neglect/abuse policy in place at any time point. There was an increase in external cause mortality, and a decrease in SIDS mortality over time. State mandatory reporting policies were not associated with changes in external cause (aIRR: 1.09, 99% CI 0.94, 1.28) or SIDS (aIRR: 1.03; 99% CI 0.85, 1.27) mortality rates. Plans of safe care policies were not associated with a change in external cause (aIRR: 1.02; 99% CI 0.82, 1.26) or SIDS (aIRR: 0.94; 99% CI 10.89, 1.51) mortality rates. Neglect/abuse policies were not associated with changes in external causes (aIRR: 1.04; 99% CI 0.92,1.18) or SIDS (aIRR: 0.93; 99% CI 0.79, 1.12) mortality rates. Findings were similar for all-cause mortality.

**Conclusion:** States’ adoption policies pertaining to how child welfare agencies address substance use in pregnancy was not associated with meaningful changes in infant mortality rates due to external causes, SIDS, or all-cause mortality in the United States.

## A90 “Changes in opioid utilization by provider type following a days’ supply restriction policy” (SW03)

### Ivelisse L. Valdes, Marie-Christin Possinger, Juan M. Hincapie-Castillo, Amie J Goodin, Marvin A. Dewar, Jill M. Sumfest, and Scott M Vouri

#### Lead Author Affiliation: Department of Pharmaceutical Outcomes & Policy at University of Florida, 1225 Center Drive Gainesville, FL 32610, HPNP Building, USA

##### **Correspondence:** Ivelisse L. Valdes (ivelissev@ufl.edu)

*Addict Sci Clin Pract* 2020, **15(Suppl 2)**:A90

**Background:** Many states have implemented opioid days’ supply restriction policies, leading to reductions in opioid prescribing. Although research within certain provider types exist, no study has evaluated an opioid restriction policy by various provider types. To evaluate changes in mean days’ supply (MDS) and morphine milligram equivalents (MMEs) dispensed per opioid prescription before and after Florida’s restriction policy (implemented on 7/1/2018), stratified by provider type: surgery, emergency medicine, primary care, and dentistry.

**Methods:** We used prescription claims of a private health plan serving a large Florida employer from 1/1/2015 to 3/31/2019. Interrupted time series analyses were conducted to compare pre and post-implementation changes in MDS and MMEs for opioid medications stratified by Healthcare Provider Taxonomy Code using providers’ national provider identifier (NPI).

**Results:** Among 8000 opioid initiators, treating providers were classified as surgery 21.7% (n = 1732), emergency medicine 19.0% (n = 1516), primary care 28.0% (n = 2241), and dentistry 31.4% (n = 2511). For surgery, the MDS was 5.4 which resulted in a non-significant decrease to 3.9 following implementation (p = 0.055) and mean MME of 212 with a non-significant reduction after implementation (p = 0.244). In emergency medicine, MDS was 3.5 with a reduction to 2.8 following implementation (p = 0.036) and mean MME was 88 with a reduction to 61 following policy enaction (p = 0.001). For primary care providers, MDS was 8.9 with a reduction to 5.7 following implementation (p = 0.011); however, the mean MME was 185 with no significant reduction after the law enacted (p = 0.219). The MDS was 3.5 for dentistry with a reduction to 3.0 following implementation (p = 0.012); however, the mean MME was 116 with no significant reduction after implementation (p = 0.557). Additionally, changes in trends of opioid prescribing varied over time following implementation by provider type.

**Conclusion:** Pre-policy opioid prescribing varied by provider type with a differential impact on MDS and MMEs dispensed per prescription following implementation.

## A91 “Comparing the current status of state laws of opioid prescribing in the United States” (SW04)

### Taylor Easey, Kayla M. Smith, Aolani B. Chirino, Carlos-Hernandez, Robin Moornman-Li, Amie Goodin, Scott Martin Vouri, and Juan M. Hincapie-Castillo

#### Lead Author Affiliation: Department of Pharmaceutical Outcomes & Policy at University of Florida, 1225 Center Drive Gainesville, FL 32610, HPNP Building, USA

##### **Correspondence:** Taylor Easey (limade987@ufl.edu)

*Addict Sci Clin Pract* 2020, **15(Suppl 2)**:A91

**Background:** In response to the epidemic of opioid-related overdoses and deaths in the United States, several states have passed legislation limiting the prescribing of opioids for acute pain. Previous studies address state-specific legislation restrictions; however, many studies failed to examine the details of these restrictions across each of the 50 states. We aimed to summarize the state-specific limitations on the day’s supply or total morphine milligram equivalents (MMEs) per day of the prescription, any individual opioid prescriber stipulation in the laws, and any exemptions to these policies.

**Methods:** We utilized publicly available legislative online libraries to search for statutes and State Code amendments pertaining to opioid restrictions through May 1, 2020. Elements collected from each state included: the specific legislative policy on prescribing of adult and pediatric patients, the practitioner to whom the law applied, exemptions to the legislation, and the timeline of policy enactment.

**Results:** In the United States, as of May 2020, 37 states have enacted an opioid prescribing restriction. Thirty of the 37 states (81%) have limits only on the number of days’ supply allowed, and 7 (19%) have limits on the MMEs and days’ supply for acute pain conditions. A total of 13 states (35%) have distinctions on what type of prescriber the restriction applied to and a total of 8 states (22%) have different limitations for pediatric patients. All states with opioid limitations have chronic pain restriction exemptions, but these vary across each individual state.

**Conclusion:** This study shows an uptake in increased state restrictions for prescribing of opioids for acute pain across the United States. Due to a lack of federal policy, there is no uniformity amongst these prescribing restrictions. Future studies evaluating national trends and opioid policy evaluations must account for the different implementation timelines of prescribing restriction laws.

## A92 “Coverage of medications for the treatment of opioid use disorder in medicaid managed care organizations” (SW05)

### Amanda J. Abraham, Melissa A. Westlake, MSW; Samantha J. Harris, Christina M. Andrews, and Colleen M. Grogan

#### Lead Author Affiliation: University of Georgia, Candler Hall, 202 Herty Dr, Athens, GA 30602, USA

##### **Correspondence:** Amanda J. Abraham (aabraham@uga.edu)

*Addict Sci Clin Pract* 2020, **15(Suppl 2)**:A92

**Background:** Medicaid is the largest payor of all substance use disorder treatment in the US, providing coverage for approximately 40% of all Americans with opioid use disorder. A majority of Medicaid enrollees (~ 54 million) participate in managed care organization (MCO) plans, however little is known regarding how MCO plans manage access to opioid use disorder medications.

**Methods:** We conducted a content analysis of all Medicaid MCO plans across 39 states in 2018 (n = 264) using publicly-available documentation (member handbooks, provider manuals, drug formularies) on coverage and utilization management policies for all medications FDA approved for opioid use disorder treatment. Descriptive statistics were used to compare coverage and utilization management policies for injectable naltrexone, buprenorphine, and methadone. For comparison, we also examined naloxone which is used for the treatment of opioid overdose.

**Results:** A little more than half (55.6%) of all Medicaid MCO plans covered all three opioid use disorder medications. Almost all MCO plans covered buprenorphine (98.1%), while about 72% of plans covered methadone or injectable naltrexone. Coverage of methadone and injectable naltrexone showed the widest variation by state. In 49% of states, all MCO plans covered methadone, while no MCO plans covered methadone in 18% of states. In 36% of states, all MCO plans covered injectable naltrexone and in 18% of states, no MCO plans covered the medication. We also found variation in utilization management policies across medications. Prior authorization was required by 53% of plans for buprenorphine, 52% for methadone and 42% for injectable naltrexone. In contrast, only 10% of plans required prior authorization for naloxone. Utilization management policies also showed variation by state. In 33% of states, all MCO plans required prior authorization for buprenorphine and in 28% of states, no MCO plans required prior authorization for the medication. In 19% of states, all MCO plans required prior authorization for injectable naltrexone and in 47% of states, no MCO plans required prior authorization for the medication. In contrast, 87% of states did not require prior authorization for naloxone in all MCO plans.

**Conclusion:** Our findings suggest that Medicaid enrollees’ access to medications may be heavily influenced by the state they live in and the particular MCO plan in which they enroll. State Medicaid agencies may need to restructure MCO contracts to ensure more equitable access to medications for the treatment of opioid use disorder.

## A93 “Current workforce development priorities for training and technical assistance for integration of behavioral health services in medical settings” (SW06)

### Denna Vandersloot, Susan Stoner, and Bryan Hartzler

#### Lead Author Affiliation: Alcohol & Drug Abuse Institute at University of Washington, 1107 NE 45th St, Seattle, WA 98105, USA

##### **Correspondence:** Denna Vandersloot (dennav@uw.edu)

*Addict Sci Clin Pract* 2020, **15(Suppl 2)**:A93

**Background:** Explore Region 10 workforce perspectives of key training and technical assistance needs to support workforce efforts to integrate behavioral health services in primary care. Herein, results are described of an online needs assessment survey completed by addiction workforce members in Health and Human Services (HHS) Region 10.

**Methods:** Seven survey items concern practices specific to integrating behavioral health services in primary care, for which importance as a workforce development priority was rated on a five-point Likert scale (1 = Not At All, 5 = Extremely). A lone inclusion criteria for survey respondents, recruited via the Northwest Addiction Technology Transfer Center (Northwest ATTC) website, was current employment as a health professional in an HHS Region 10 state (i.e., AK, ID, OR, WA).

**Results:** Among this addiction workforce sample (N = 306), the three practices most highly-rated as integration workforce development priorities were: (1) multidisciplinary staff teamwork to meet the clinical challenges (M = 4.31, SD = 0.89), (2) orientation to substance use disorders as a form of chronic illness (M = 4.23, SD = 0.89), and (3) referral processes to link persons to longer-term treatment (M = 4.20 SD = 0.89).

**Conclusions:** Findings identify targets the Region 10 workforce members see as most important for integrating behavioral health services into primary care. These findings suggest a need to focus on providing training and technical assistance to promote skills related to multidisciplinary staff teamwork, orientation to substance use disorders (SUD) as a form of chronic illness, and referral processes to link persons to longer-term treatment. This will inform future efforts by the Northwest ATTC, and others similarly seeking to address workforce development issues, around integration of addiction services into primary care settings.

## A94 “Density of cannabis retailers and cannabis use disorder for veteran health affairs patients in King County, Washington” (SW07)

### Theresa E. Matson, Gwen T. Lapham, Jonathan C. Wakefield, and Emily C. Williams

#### Lead Author Affiliation: University of Washington School of Public Health, 1959 NE Pacific St, Seattle, WA 98195, USA

##### **Correspondence:** Theresa E. Matson (tessam2@uw.edu)

*Addict Sci Clin Pract* 2020, **15(Suppl 2)**:A94

**Background:** Availability and use of cannabis is changing rapidly in the United States as legislation expands legal markets for cannabis. Increased access to cannabis retailers and products may increase likelihood of cannabis use disorder (CUD). We examine whether density of cannabis retailers is related to prevalence of CUD for Veterans Health Affairs (VA) patients in King County, Washington.

**Methods:** This ecological, cross-sectional study considered 85 zip codes in King County (includes Seattle) where recreational and medical cannabis is legal. Counts of active cannabis retailers within zip codes were obtained from the Washington State Liquor and Cannabis Board. VA patients living in King County were included if they had ≥ 1 electronic health record-documented visit over 2 years (7/8/2015–7/31/2017). CUD was determined by the presence of ≥ 1 diagnostic code in the year prior to the visit, and counts of patients with CUD were aggregated by zip code. Using Poisson-lognormal spatial random effects models, fit using integrated nested Laplace approximation, we estimated the relative risk of CUD associated with cannabis retailer density, adjusted for population characteristics (age, race, gender) at the zip-code level. Posterior median estimates of relative risk were extracted for all zip codes and mapped to explore spatial structuring.

**Results:** A total of 91 cannabis retailers and 19,246 VA patients contributed data to analyses. A majority of patients were older (median age = 62), male (90%), and white (80%). The average number of observed CUD cases in each zip code was 5.0 (0–37). Spatial variability in CUD risk was observed across zip codes. One additional cannabis retailer was associated with a 7.6% (95% credible interval: 0.9%–14.4%) increase in area-level CUD risk.

**Conclusion:** Areas in King County, Washington with a high density of cannabis retailers also had a higher prevalence of VA patients with CUD. Further research is needed to understand temporality of this association.

## A95 “Disclosure to healthcare providers as a factor in alcohol abstinence” (SW08)

### Kiefer Cowie, and Emily Diamond

#### Lead Author Affiliation: The Wright Institute, 2728 Durant Ave, Berkeley, CA 94704, USA

##### **Correspondence:** Kiefer Cowie (kcowie@wi.edu)

*Addict Sci Clin Pract* 2020, **15(Suppl 2)**:A95

**Background:** Social determinants are factors in length of sobriety in alcohol recovery. This study examined the relationship between disclosure of alcohol recovery status and longest period of alcohol abstinence.

**Methods/Results:** US adults (n = 154) participated in an online study launched simultaneous to the emergence of the COVID-19 pandemic. Limiting stress and potential relapse, nearly all questions were optional.

**Results:** Participants were (76 male, 77 female, 1 transgender) mean age (55 male; 50.8 female), and 94% had active healthcare insurance. Age of first alcohol consumption was similar between genders, (mean = 15 years old), the mean age for self-identifying problematic alcohol consumption was 30 (28.8 male, 31.2 female). Overall there were significant differences by gender in longest mean duration of abstinence (male 8.48 years; female 5.77 years) (p < 0.01; Cohen’s d = 0.440). There was a significant difference between number of disclosure groupings and longest abstinence (ranked ANOVA, p < 0.001; Cohens f = 0.605). Mean longest period of abstinence was increased for those who disclosed to grandparents, parents, children, employers, support group members, and healthcare providers. Those who disclosed to health care providers (n = 95) compared with those who had not (n = 57) had a significant difference in longest mean abstinence (8.39 vs. 4.97 years) (p = 0.011; Cohen’s d = 0.416). Among those with medical conditions (n = 73) and those without (n = 80), longest mean abstinence was approximately double (9.66 vs. 4.80 years), (p < 0.001; Cohen’s d = 0.621). This was similar to disclosing to close family members.

**Conclusion:** Disclosure to health care providers was associated with significantly longer abstinence, particularly for those with medical conditions. Findings indicate the importance of disclosing recovery status to health care providers, working to reduce the time to seeking treatment, and identifying problematic alcohol consumption earlier. As those with medical condition had longer sobriety, more integrated care could be beneficial.

## A96 “Documentation of suicidality is negatively associated with receipt of first-line treatment medications for opioid use disorder in a national sample of veterans health administration patients” (SW09)

### Madeline C. Frost, Julie E. Richards, John R. Blosnich, Eric J. Hawkins, Judith I. Tsui, E. Jennifer Edelman, and Emily C. Williams

#### Lead Author Affiliation: University of Washington School of Public Health, 1959 NE Pacific St, Seattle, WA 98195, USA

##### **Correspondence:** Madeline C. Frost (Madeline.Frost@va.gov)

*Addict Sci Clin Pract* 2020, **15(Suppl 2)**:A96

**Background:** Opioid use disorder (OUD) and suicide are urgent crises. OUD is associated with increased suicidality (ideation/attempt). It is important that patients with suicidality and OUD receive evidence-based medications to treat OUD (MOUD), which may lower risk of suicide. MOUD include agonists (methadone/buprenorphine) which are first-line treatments that reduce opioid use and overdose risk, and an antagonist (injectable naltrexone) which reduces craving and use. It is unknown whether suicidality impacts likelihood of receiving MOUD. We examined whether documented suicidality was associated with MOUD receipt among Veterans Health Administration (VA) patients with OUD.

**Methods:** Electronic health record data were extracted for all VA outpatients with ≥ 1 preventive screen indicating a visit (10/1/09–7/31/17) with prior-year documentation of OUD diagnosis. In cross-sectional analyses using most recent visit, Poisson regression with robust standard errors clustered on facility estimated relative risk (RR) of receiving MOUD (≥ 1 clinic code/filled prescription for methadone, buprenorphine, and/or injectable naltrexone in years prior to/following visit) for patients with suicidality (≥ 1 diagnostic code/risk flag indicating prior-year ideation/attempt) relative to those without. Models were adjusted for sociodemographics and comorbidities. Secondary analyses examined individual medications.

**Results:** Among 88,207 patients, 12.8% (n = 11,313) had documented suicidality. Suicidality was negatively associated with receipt of any MOUD (RR 0.82, 95% confidence interval [CI] 0.77–0.86). Results were similar for methadone and buprenorphine individually, but suicidality was positively associated with injectable naltrexone receipt (RR 1.40, 95% CI 1.17–1.67).

**Conclusion:** Among VA outpatients with OUD, those with documented suicidality may be less likely to receive any MOUD and first-line agonists (methadone/buprenorphine), but more likely to receive injectable naltrexone. Longitudinal analyses are planned to establish temporality between suicidality and subsequent MOUD receipt, as associations might reflect reverse causation (i.e., MOUD may impact likelihood of suicidality). Health system interventions may be needed to ensure patients with OUD and suicidality receive life-saving medications.

## A97 “From policy to practice: the real-world impacts of medicaid system transformation on substance use disorder treatment programs” (SW10)

### Howard Padwa, Lesley Harris, Veronica Serret, Tenie Khachikian, and Erick Guerrero

#### Lead Author Affiliation: University of California, Los Angeles Integrated Substance Abuse Programs, 11075 Santa Monica Blvd., Ste. 200, Los Angeles, CA 90025, USA

##### **Correspondence:** Howard Padwa (hpadwa@mednet.ucla.edu)

*Addict Sci Clin Pract* 2020, **15(Suppl 2)**:A97

**Background:** The Affordable Care Act (ACA) and MediCal 1115 Waiver for substance use disorder (SUD) services in California required SUD treatment organizations to integrate themselves into medical systems of care for reimbursement. Our main research question was to learn the anticipated impacts of ACA and the MediCal Waiver on service delivery pre-implementation, and how these changes impacted these organizations and access and engagement in care post-implementation.

**Methods:** A pre- and post-ACA longitudinal qualitative research design was developed to understand policy implementation regarding in SUD treatment organizations. Informed by constructive grounded theory, we rely on Dedoose to analyze an average of 30 semi-structured interview transcripts with clinical supervisors in each of the three waves (2013, 2015, and 2017). These supervisors have played a key role in the implementation of system-wide reforms and integration of care.

**Results:** In 2013, supervisors anticipated increased service utilization but with a strict managed care approach to justify medical necessity leading to a loss of control over treatment decisions. Supervisors also anticipated significant need for workforce training challenging their ability to satisfy increased service demand. Following ACA implementation (2015), supervisors reported an increased emphasis on evidence-based practices, pharmacotherapy, harm-reduction, a clientele with higher SUD severity, and higher rates of co-occurring medical conditions. In 2017, supervisors reported that the county implementation of the 1115 Waiver accelerated these trends making it difficult for providers to adapt to an environment that seemed to be perpetually changing.

**Conclusions:** The ACA and 1115 Waiver have changed the client population and services that SUD organizations need to provide. The major changes brought by about these policies in rapid succession proved challenging for treatment providers. Programs may benefit from more gradual implementation of system transformation, or greater support to ramp up organizational and clinical capacity during times of transition.

## A98 “Modeling the impact of naloxone distribution for overdose prevention through community programs, prescriptions, and pharmacy-facilitated channels in the US: results from a 10-state analysis” (SW11)

### Michael Irvine, Declan Oller, Brian Bishop, Jesse Boggis, Dan Coombs, and Traci Green

#### Lead Author Affiliation: British Vancouver Children’s Hospital Research Institute, 938W 28th Ave, Vancouver, BC V5Z 4H4, Canada

##### **Correspondence:** Michael Irvine (Mike.Irvine@bcchr.ca)

*Addict Sci Clin Pract* 2020, **15(Suppl 2)**:A98

**Background:** The opioid crisis claimed the lives of over 47,000 Americans in 2017. Equipping people with the opioid overdose antidote naloxone can reduce the rate of fatal overdose. This study aimed to estimate the number of naloxone kits needed to reduce overdose risk in a sample of 10 US states across a range of access points.

**Methods:** We constructed a Bayesian model of people at risk of opioid overdose and fitted to prescription, heroin, and fentanyl-dominant state-specific epidemics using 2017 data. We performed a literature review and modified-Delphi panel to estimate parameters linked to naloxone need. Overdose death, paramedic-attended overdose, and at-risk population data were used to calibrate the model for 10 states: MA, RI, NC, SC, OK, AZ, CA, ID, OR, and WA. We measured naloxone saturation using potentially fatal overdose deaths averted and probability of witnessed overdose reversed. We explored the impact on mortality if community program naloxone kits were distributed across 9 states at the same rate as MA.

**Results:** In 2017, there were 12,086 overdose related deaths across 10 states. We estimated 27,199 overdoses were averted by naloxone, resulting in 3350 averted deaths. Community program and pharmacy-facilitated distribution were more available compared with dispensed prescription, however, no state attained saturation. The highest probability of naloxone use during a witnessed overdose was in RI (60.3%; 95% CI 43.2%–85.1%). If MA community program distribution had been applied across all states, 12,958 deaths could have been averted. Within MA, naloxone use was attributed to community programs (68.0%), pharmacy-facilitation (22.7%) and dispensed prescriptions (9.3%).

**Conclusion:** Naloxone distribution efforts in 10 states are far from attaining maximum reach. Community program and pharmacy-facilitation can avert more overdose deaths and extend greater likelihood that naloxone will be used during a witnessed overdose.

## A99 “Barriers to accessing medication-assisted treatment among pregnant women with opioid use disorder: a comprehensive literature review” (SW12)

### Laura Curran

#### Lead Author Affiliation: New York University Silver School of Social Work, 1 Washington Square N, New York, NY 10003, USA

##### **Correspondence:** Laura Curran (lec468@nyu.edu)

*Addict Sci Clin Pract* 2020, **15(Suppl 2)**:A99

**Background:** The recommended treatment for opioid use disorder (OUD) in pregnancy is a comprehensive treatment program that includes the initiation of medication-assisted treatment (MAT). Despite its effectiveness, MAT use remains low; only 39% of pregnant women with OUD who admitted to a treatment facility in the U.S. in 2012 received any methadone or buprenorphine (Martin 2014). Expanding access to MAT for pregnant women is critical. A comprehensive review of the literature was conducted to identify the state, community, and individual level barriers among pregnant women with OUD in initiating MAT. This review aimed to broaden conventional views of access as synonymous with treatment availability by exploring multi-level barriers.

**Methods:** Peer-reviewed studies from 2000 to 2019 were identified using these search terms: opioid use disorder, pregnant, treatment, access, barriers, and substance use. Qualitative, quantitative, mixed methods studies, and policy statements were included. Studies were included if they were aimed at addressing challenges that prevent pregnant women from accessing MAT at the policy, legal, community, and individual levels. A total of 33 articles were reviewed.

**Results:** Findings revealed ten major barriers to MAT initiation for pregnant women: (1) lack of private or Medicaid insurance coverage, (2) jail or drug court procedures, (3) child abuse statutes criminalizing maternal drug use, (4) limitations in recent healthcare laws and policies, (5) gaps in healthcare systems related to fragmented screening and referral procedures, stigma and fear, (7) geographic barriers, (8) lack of provider certification to prescribe buprenorphine, (9) lack of provider understanding and unwillingness to provide treatment, and (10) economic barriers.

**Conclusions:** These findings highlight the need for improvement in healthcare policies that increase the availability of providers who understand the needs of pregnant women and who are certified to prescribe buprenorphine. A summary of policy statements points to the importance of early identification of OUD and universal screening. Laws and policies should avoid punitive measures, and efforts should be made to consistently link pregnant women with MAT by implementing multidisciplinary, collaborative approaches across systems of care.

## A100 “Operationalizing person-centered care in behavioral health treatment: how can treatment centers implement “respect for patients’ values, preferences, and expressed needs?” A qualitative study” (SW13)

### Olivia Randall-Kosich, Barbara Andraka-Christou, Rachel Totaram, Kendall Cortelyou-Ward, Danielle Atkins, and Andriy Koval

#### Lead Author Affiliation: Georgia State University School of Public Health, 140 Decatur St SE, Atlanta, GA 30303, USA

##### **Correspondence:** Olivia Randall-Kosich (orandallkosich1@gsu.edu)

*Addict Sci Clin Pract* 2020, **15(Suppl 2)**:A100

**Background:** To identify specific methods that behavioral health centers can implement to fulfill the first dimension of Picker Institute’s eight dimensions of person-centered care, “respect for patients’ values, preferences and expressed needs.”

**Methods:** Administrators, providers, peer support specialists, and current and former clients of South Florida behavioral health treatment centers were recruited by South Florida Behavioral Health Network (SFBHN). Interviews occurred over the telephone, were audio recorded and transcribed. We then created a codebook based on the research questions and preliminary review of transcripts. We independently coded transcripts and then resolved discrepancies through negotiation. Coded excerpts were analyzed to find specific operationalization methods.

**Results:** We recruited 38 participants: 26 past or current clients, 9 peer support specialists, and 9 employees (participants could designate more than one role). Most participants worked at/attended a facility that provided both mental health and substance use services (89%), offered both residential and outpatient levels of care (87%), and was affiliated with the SFBHN (71%). Participants identified the following methods for treatment centers to implement respect for patient values, preferences and needs: offering alternatives to twelve-step peer support groups; offering programming in non-English languages; helping clients attend religious services; displaying visible signs of appreciation of demographic diversity; asking which treatment methods have/have not worked in the past for the client; allowing clients to temporarily leave residential treatment centers; offering a menu of services instead of a preset schedule; allowing clients to choose harm reduction goals; and offering confidential ways for clients to file grievances.

**Conclusion:** Operationalization methods included offering “non-traditional” services (e.g., non-twelve-step peer support groups, all types of MAT), providing services specifically for clients from diverse backgrounds, and allowing clients to make treatment decisions and request changes.

## A101 “Opioid treatment in a pandemic: piloting a NYC-wide virtual buprenorphine clinic in response to COVID-19” (SW14)

### Noa Krawczyk, Daniel Schatz, Jennifer McNeely, Adam Demner, Timothy Reed, and Babak Tofighi

#### Lead Author Affiliation: New York University Grossman School of Medicine, 550 1st Avenue, New York, NY 10016, USA

##### **Correspondence:** Noa Krawczyk (noa.krawczyk@nyulangone.org)

*Addict Sci Clin Pract* 2020, **15(Suppl 2)**:A101

**Background:** Barriers to buprenorphine treatment, already rampant for patients with opioid use disorders (OUD), may be exacerbated during the Covid-19 pandemic. Telemedicine approaches may increase OUD treatment access, but until recently were largely restricted. In response to shifts in federal treatment regulations and the urgent need to mitigate risks of COVID-19, NYC’s public Health + Hospitals system launched a city-wide Virtual Buprenorphine Clinic (VBC). Here, we describe the VBC model, and present initial patient characteristics and outcomes.

**Methods:** The VBC is based on the medical management model which centers on a primary care approach to OUD treatment. Eligible patients can initiate same-day buprenorphine via a telemedicine visit with a physician visit followed by care management calls to assist with overdose education and linkage to needed health and social services.

**Results:** In its first 2 months, the VBC initiated 78 patients. Patients were primarily male (84%), with a mean age of 45. 22% had been released from prison/jail in the past month, 49% were homeless, and 22% did not own a personal phone. Referral sources for patients came primarily from non-hospital health providers (29%), homeless shelters (17%) and jail-reentry programs (15%). Since initiation, 27% of patients have been referred to ongoing care. Only one non-fatal overdose event has been reported among patients.

**Conclusions:** Buprenorphine provision through telemedicine provides a safe and feasible way of reaching OUD patients with multiple vulnerabilities while maintaining safety precautions for COVID-19. Sustainability of low-barrier telemedicine MOUD programs will depend on permanent changes to treatment regulations and building financially viable models to support adjunctive services that may be important for encouraging safety and retention.

## A102 “Opioid use disorder among patients receiving public mental health services: prevalence, characteristics and treatment willingness” (SW15)

### Allison Ober, Sarah B. Hunter, Colleen M. McCullough, Michael McCreary, Ivan Beas, Alanna Montero, Derjung M. Tarn, Elizabeth Bromley, Brian Hurley, John Sheehe, Jeremy Martinez, Isabel Leamon, and Kate E. Watkins

#### Lead Author Affiliation: RAND Corporation, 1776 Main St. Santa Monica, CA 90401-3208, USA

##### **Correspondence:** Allison Ober (ober@rand.org)

*Addict Sci Clin Pract* 2020, **15(Suppl 2)**:A102

**Background:** Despite the effectiveness of medication for opioid use disorder (MOUD), OUD among those with mental illness largely goes untreated. Understanding OUD prevalence and treatment preferences among people receiving treatment in public mental health clinics is needed to facilitate uptake of MOUD among people with co-occurring disorders.

**Methods:** Adults presenting for an appointment over a 2-week period within 8 clinics of the Los Angeles County Department of Mental Health were asked if they would like to complete an anonymous, tablet-based wellness survey. Clients who indicated opioid use in the past three months on a screener were directed to the survey. Probable OUD was assessed using the ASSIST. Willingness to take a medication and receive any treatment was assessed on a scale from 0 to 100.

**Results:** 3090 individuals completed the screening. 9% had a probable prescription (RX) OUD; 2% had a probable heroin OUD. 52% of those with probable RX OUD were female compared to 30% with probable heroin OUD. Of those with probable RX OUD, 43% were Black, 33% were Hispanic and 12% were White; of those with probable heroin OUD 24% were Black, 22% were Hispanic, and 39% were White. Depression and bipolar disorder were the most common reasons for seeking treatment among those with probable OUD (32% and 16%, respectively). The strongest predictor of willingness to take buprenorphine or naltrexone for an OUD was the belief that the medication would help stop opioid use (β = 13.10, p < 0.01); (β = 14.93, p < 0.01, respectively); having probable heroin OUD compared with RX OUD was associated with greater willingness to receive any OUD treatment in a mental health setting.

**Conclusion:** OUD is prevalent among those seeking services in public mental health settings; people with heroin OUD and those who believe in the benefits of the medication are most amenable to receiving MOUD.

## A103 “Patient attitudes toward substance use screening and discussion in primary care” (SW16)

### Leah Hamilton, Sarah E. Wakeman, Timothy Wilens, Joseph Kannry, Richard N. Rosenthal, Keith Goldfeld, Angeline Adam, Noa Appleton, Sarah Farkas, Carmen Rosa, John Rotrosen, and Jennifer McNeely

#### Lead Author Affiliation: New York University Grossman School of Medicine, 550 1st Avenue, New York, NY 10016, USA

##### **Correspondence:** Leah Hamilton (leah.hamilton@nyulangone.org)

*Addict Sci Clin Pract* 2020, **15(Suppl 2)**:A103

**Background:** Alcohol and drug use are often under-identified in primary care settings. Although prior research indicates that patients are generally supportive of alcohol screening, less is known about attitudes toward drug screening or the collection of this information in electronic health records (EHRs). As a part of an implementation study of EHR-integrated substance use screening in primary care, conducted in the NIDA Clinical Trials Network, patients were surveyed on their attitudes toward screening for substance use during medical encounters.

**Methods:** Surveys were administered to patients in six urban academic primary care clinics following the introduction of a screening program. Participants were recruited from the waiting room and self-administered an 18-item survey exploring attitudes toward screening for alcohol and drug use, and discussing substance use with healthcare providers.

**Results:** Participants (N = 553; mean age 54.2; 58.9% female; 60.4% white, 21.7% Black; 5.6% Asian, 17.9% Hispanic/Latino) overwhelmingly felt that they should be asked about their substance use (91%), and deemed it appropriate for their doctor to recommend reducing use if it adversely affects their health (92%). Most (87%) were equally comfortable discussing alcohol or drug use. 63% preferred discussing substance use with their doctor over other medical staff. Responses were mixed regarding screening modality: 55% preferred face-to-face, 25% had no preference, 19% preferred self-administration. Participants reported that they would be honest with their provider (94%), but 30% were concerned about their medical record confidentiality.

**Conclusion:** Patients strongly supported screening for drug and alcohol use in primary care, and discussing it with their doctor. However, patients’ concerns about having their substance use documented in their medical record could pose a barrier to achieving accurate responses. These findings suggest a need to educate patients on the confidentiality of medical records and the value of disclosing substance use for their medical care.

## A104 “Patient perspectives regarding universal self-administered screening for tobacco and cannabis in a large health care system” (SW17)

### Lillian Gelberg, Whitney Akabike, Sophie Feller, Efren Aguilar, Julia Kim, Roya Ijadi-Maghsoodi, and Steven Shoptaw

#### Lead Author Affiliation: University of California, Los Angeles, 1920 Colorado Ave., Santa Monica, CA 90404, USA

##### **Correspondence:** Lillian Gelberg (lgelberg@mednet.ucla.edu)

*Addict Sci Clin Pract* 2020, **15(Suppl 2)**:A104

**Background:** Tobacco and cannabis co-use is common and expected to increase in California with recent legalization of recreational cannabis. Studies have shown increased validity of self-reports of sensitive behaviors through patient self-administered computer-based assessment methods. We sought to understand patient attitudes regarding implementation of a self-administered computerized universal screener via the EMR patient portal for tobacco and cannabis use and second-hand exposure among all UCLA primary care patients.

**Methods:** We conducted 3 focus groups with adult UCLA patients (N = 23, 91% Female) to explore patient views and experiences in relation to tobacco and cannabis use. Participants discussed thoughts about their primary care physician asking about use and secondhand exposure, for themselves and their children; benefits and concerns of screening; how to implement screening; and neighborhood factors influencing use and exposure. Focus group sessions were audio-recorded, transcribed, and analyzed using content analysis.

**Results:** Barriers to screening for all patients for tobacco and cannabis use included concern about privacy of records and time spent completing the questionnaires. Some patients felt it was beneficial to screen youth for tobacco and cannabis use, including as an opportunity to educate youth about the consequences of use, while expressing concern that youth may not disclose use due to confidentiality concerns. Patients described neighborhood influences contributing to use, availability of cannabis, and for the youth, peer pressure.

**Conclusions:** Implementing a patient self-administered computerized tobacco and cannabis screener among primary care patients in a large health system may be a useful tool to provide patient education. However, patients may have concerns about screening that include privacy concerns and ramifications of disclosure, time spent on the screening, and honesty of disclosing use among youth. Understanding patient perceptions of screening for tobacco and cannabis use and exposure, and recommendations for computerized screening, can inform health systems when implementing universal tobacco and cannabis screening. Financial Support: This work was supported by the Tobacco-Related Disease Research Program of California (TRDRP).

## A105 “Prescription drug monitoring program rigor and opioid use in patients with cancer” (SW18)

### Tham T. Le, Sean P Fleming, Aida Kuzucan, and Linda Simoni-Wastila

#### Lead Author Affiliation: University of Maryland, Baltimore, 20 North Pine Street, Baltimore, Maryland 21201, USA

##### **Correspondence:** Tham T. Le (tham.le@umaryland.edu)

*Addict Sci Clin Pract* 2020, **15(Suppl 2)**:A105

**Background:** Prescription Drug Monitoring Programs (PDMP) differ significantly in their scope and rigor but their effects on pain management in cancer patients remains unknown. This study examined the impact of PDMP rigor on opioid utilization among patients with cancer.

**Methods:** A retrospective cohort study of new cancer patients aged 18–64 years in IQVIA PharMetrics* Plus. Study participants were continuously enrolled 12 months pre- and post- cancer diagnosis. PDMP rigor was assessed in states by registration and query mandates and classified into four categories: registration AND query, query only, registration only, and no mandate exposure (reference). Primary outcomes included individual-level opioid prevalent use and cumulative morphine equivalent dose (MED) in the 12-month post-period. To account for patients’ correlation within a state, generalized estimating equations modeled prevalent opioid use among all individuals; generalized linear models with robust sandwich estimators modeled cumulative MED among opioid users.

**Results:** Of 28,353 eligible individuals, 3899 (13.8%), 3459 (12.2%), 2764 (9.7%), and 18,231 (64.3%) exposed to registration AND query, query only, registration only, and no mandates, respectively, and 10,656 (37.6%) were prescribed opioids post-cancer. Prevalent opioid use was not significantly associated with registration and/or query mandates. Query mandates, alone or with registration mandates, were associated with lower mean cumulative MED (− 662 and − 702 mg, p < 0.01, respectively) compared to unexposed. Registration only mandates had no significant effect on cumulative MED (− 46 mg, p > 0.05).

**Conclusions:** Lower cumulative MED in cancer patients exposed to query mandates suggests that query mandates are a stronger tool used to reduce opioid dispensing in PDMPs than registration mandates, which may inadvertently lead to under-treatment of cancer pain. Policy makers should consider the impact of regulatory mandates in PDMPs intended to reduce high-risk opioid utilization in the general population without compromising unintended effects on at-risk patients.

## A106 “Relationship between substance use disorder (SUD) and healthcare fragmentation patterns in veterans at high-risk for hospitalization” (SW19)

### Fernanda Rossi, Christine Timko, Sarah J. Javier, Donna M. Zulman, Liberty Greene, Sara Singer, Megan E. Vanneman, Mary Goldstein, and Ranak Trivedi

#### Lead Author Affiliation: VA Palo Alto Health Care System and Stanford University, 3801 Miranda Avenue, Palo Alto, CA 94304, USA

##### **Correspondence:** Fernanda Rossi (fsrossi@stanford.edu)

*Addict Sci Clin Pract* 2020, **15(Suppl 2)**:A106

**Background:** Fragmented healthcare can pose potential risks to patients when they receive inefficient and poorly coordinated services. The extent to which fragmentation characterizes the healthcare of patients with chronic physical health conditions and co-occurring substance use disorders (SUD) is unknown. Patients with SUD and chronic medical comorbidities require extensive and integrated care, making it critical to examine healthcare fragmentation in this population. This study compared healthcare fragmentation for chronic physical conditions between Veterans with and without SUD.

**Methods:** The sample included Veterans Affairs (VA) patients at high risk for hospitalization (Care Assessment Need risk score ≥ 90th percentile). We identified Veterans with ≥ 4 non-mental health VA or VA-purchased care visits during FY14 to increase variability in our measures of healthcare fragmentation. Data were acquired from VA Corporate Data Warehouse. Outcomes were two fragmentation measures calculated in FY14: (1) count of non-mental health providers—a higher number indicates more fragmentation, and (2) Usual Provider of Care (UPC), the proportion of care with the most frequently seen non-mental health provider—a lower number indicates more fragmentation. We used Poisson regression and fractional logistic regression to test the association between the presence of SUD (using ICD-9 codes) and fragmentation, controlling for sociodemographic characteristics, medical comorbidity, and driving distance to VA.

**Results:** Of 424,451 Veterans, 20.8% had SUD. Compared to Veterans without SUD, those with SUD saw fewer providers (IRR = 0.90) (provider count: M = 5.9 SD = 3.3 range: 1–43) and had a higher UPC (more concentrated care; OR = 1.12) (UPC: M = 0.4 SD = 0.2 range: 0.04–1).

**Conclusion:** Within the VA, high-risk Veterans with SUD do not experience greater healthcare fragmentation. Findings may reflect differential service needs or consolidation of services for Veterans with SUD. Additional research is needed clarifying care coordination patterns in this population.

## A107 “Toward a typology of office-based buprenorphine treatment laws: themes from a review of state laws” (SW20)

### Barbara Andraka-Christou, Adam Gordon, Kathryn Bouskill, Rosanna Smart, Olivia-Randall-Kosich, Matthew Golan, Rachel Totaram, and Bradley D. Stein

#### Lead Author Affiliation: Department of Health Management and Informatics at University of Central Florida, 4000 Central Florida Blvd, Orlando, FL 32816, USA

##### **Correspondence:** Barbara Andraka-Christou (barbara.andraka@ucf.edu)

*Addict Sci Clin Pract* 2020, **15(Suppl 2)**:A107

**Background:** Buprenorphine is a gold standard treatment for opioid use disorder (OUD). Some US states have passed laws regulating office-based buprenorphine treatment (OBBT) for OUD, with requirements for OBBT providers beyond those required in federal law. We sought to identify themes in state OBBT laws as a first step toward creating a typology of laws regulating OUD treatment access and treatment provision.

**Methods:** Using search terms related to medications for OUD, we conducted searches in Westlaw software for state regulations and statutes in 51 US jurisdictions from 2005 to 2019. We identified OBBT laws and inductively analyzed them for themes using Dedoose software.

**Results:** Since 2005, ten states have passed a total of 181 OBBT laws. We identified the following themes: (1) provider credentials: state-level licensure for OBBT providers, continuing medical education requirements, supervision requirements for physician-extenders, and state registration requirements; (2) new patients: objective symptoms patients must have prior to receiving OBBT and exceptions for special populations; (3) educating patients: general informed consent requirements, and specific information to provide; (4) counseling: minimum counselor credentials, minimum counseling frequency, counseling alternatives; (5) patient monitoring: required prescription drug monitoring checks, frequency of drug screening, and responses to lost/stolen medications; (6) effective care: evidence-based treatment protocols, minimum clinician-patient contact frequency, health assessment requirements, and individualized treatment planning); and (7) patient safety: reconciling prescriptions, dosage limitations, naloxone co-prescribing, tapering, and office closures.

**Conclusions:** US state laws vary widely in the extent to which they place requirements beyond federal law on OBBT providers. Some laws codify practices for which scientific consensus is lacking. Additionally, some OBBT laws resemble opioid treatment program and pain management regulations. Results could serve as the basis for a typology of laws impacting OUD treatment and contribute to efforts to empirically examine how state policies affect treatment access and quality.

## A108 “Trends in opioid use alone and in combination with adjuvant medications during long-term care nursing home (LTC) stays, 2011–2015” (SW21)

### Aida Kuzucan, and Linda Simoni-Wastila

#### Lead Author Affiliation: University of Maryland, Baltimore, 620W Lexington St, Baltimore, MD 21201, USA

##### **Correspondence:** Aida Kuzucan (aidakuzucan@gmail.com)

*Addict Sci Clin Pract* 2020, **15(Suppl 2)**:A108

**Background:** Despite increased focus on opioid use in the general population, studies on opioid use alone and in combination with pain adjuvant medications, in LTC populations are lacking.

**Methods:** We linked 2011 to 2015 annual files from LTCfocus.org data, a 5% random sample of Medicare beneficiary claims (CCW), and the Minimum Data Set 3.0 (MDS) to identify a cohort of non-comatose LTC stays (> 100 custodial days) with continuous Medicare Parts A,B&D coverage. Any opioid use, and opioid use in combination with one or more pain adjuvant medications (NSAIDs, acetaminophen, triptans, anticonvulsants, muscle relaxants (MR), tricyclic antidepressants, and other antidepressants) was identified in the general population and among residents with Alzheimer’s and related dementias (ADRD), cancer, non-cancer chronic pain (NCCP), and in hospice. To quantify annual changes, we used a generalized estimating equation (with binomial distribution), adjusting for facility (resident population, occupancy and size, ownership, available special units, hospitalization rate, and staffing) and resident (age, sex, race, original reason for entitlement, follow-up time) factors.

**Results:** We found 84,529 LTC residents with 122,970 linked LTC stay-years. Opioid use was found in 40.5% of all LTC, 41.7% of hospice, 50.1% of cancer, 47.4% of NCCP, and 36.4% of dementia stays. From 2011 to 2015, analyses adjusted for facility and resident characteristics did not indicate constant or significant changes in dose, duration, or frequency of opioid use in general, hospice, cancer, NCCP, or dementia LTC related stays. Odds of opioid + skeletal muscle relaxant use increased by 81%, 26%, 75%, 114%, and 116% in the general, hospice, NCCP, cancer, and dementia LTC stay-years, respectively. Odds of opioid + anticonvulsant use also increased across stay-years, with odds in 2015 31%, 8%, 32%, 39%, and 33% greater than 2011 among general, hospice, NCCP, cancer, and dementia LTC stay-years, respectively.

**Conclusion:** Opioid use in combination with anticonvulsants and MR rose significantly among LTC stays from 2011 to 2015. More study into the safety of these medication combinations in these populations is warranted.

## A109 “TxMOUD: using SHIFT-evidence for statewide implementation of medication for opioid use disorder” (SW22)

### Shaun Jones, Sedona Koenders, Suyen Schneegans, Kristen Rosen, Holly Lanham, Erin Finley, and Jennifer Sharpe Potter

#### Lead Author Affiliation: University of Texas Health Science Center at San Antonio, 7703 Floyd Curl Dr, San Antonio, TX 78229, USA

##### **Correspondence:** Shaun Jones (joness8@uthscsa.edu)

*Addict Sci Clin Pract* 2020, **15(Suppl 2)**:A109

**Background:** Texas Medication for Opioid Use Disorder (TxMOUD) is a state-wide initiative to expand access to evidence-based treatment for opioid use disorder (OUD). TxMOUD has two key components: a training and technical assistance hub and funding for medication and associated treatment services for those without access. To ensure orderly, theory-based implementation across a complex healthcare landscape, we drew upon the Successful Healthcare Improvement for Translating Evidence in complex systems (SHIFT-Evidence) framework to guide implementation and evaluation.

**Methods:** Initial planning was informed by systematic review of barriers to buprenorphine treatment and findings from stakeholder engagement activities in a previous project, GetWaiveredTX. To track use of implementation and evaluation strategies across each SHIFT-Evidence level (patient, provider, microsystem, and macrosystem), our transdisciplinary team developed a conceptual framework to summarize TxMOUD activities. We delineated each activity ordered by level of implementation and identified corresponding implementation strategies. Each activity was cross-referenced with previously identified barriers to ensure all barriers were addressed and foreseeable concerns minimized.

**Results:** The resulting comprehensive SHIFT-Evidence framework informs the ongoing implementation and evaluation of our OUD treatment initiative. A major advantage of this framework is that it allows us to rapidly review our activities and address emergent barriers informed by evaluation data. In the spirit of SHIFT-evidence, we are flexible and pragmatic in upholding this framework, making revisions as necessary when strategies are no longer appropriate or require refinement.

**Conclusion:** We developed this SHIFT-Evidence framework for TxMOUD as a pragmatic and robust decision-making tool to support systematic implementation and evaluation of a large-scale OUD treatment initiative. Integration of novel implementation science frameworks and approaches can support rapid and effective translation of addiction science into practice while accounting for the unique needs and resources of diverse settings and patient populations.

## A110 “A pilot randomized controlled trial of a video directly observed therapy intervention delivered via mobile health application to patients receiving office-based opioid use disorder treatment” (TD01)

### Judith I. Tsui, Brian G. Leroux, Zachery A. Schramm, Andrea C. Radick, Colleen Labelle, Matthew Heerema, Kendra Blalock, Jared W. Klein, Joseph O. Merrill, Andrew J. Saxon, Jeffrey H. Samet, and Theresa W. Kim

#### Lead Author Affiliation: University of Washington, Division of General Internal Medicine Harborview Medical Center, Box 359780 325 Ninth Ave, Seattle, WA 98104, USA

##### **Correspondence:** Judith I. Tsui (tsuij@uw.edu)

*Addict Sci Clin Pract* 2020, **15(Suppl 2)**:A110

**Background:** To conduct a pilot randomized controlled trial evaluating the treatment effects of a smartphone video directly observed therapy (DOT) application for patients who recently enrolled in office-based opioid use disorder treatment with buprenorphine.

**Methods:** Adults (≥ 18 years old) prescribed sublingual buprenorphine for < 28 days were recruited from office-based programs at two urban medical centers and randomized to video DOT (intervention)—delivered via a HIPAA-compliant, asynchronous, mobile health technology platform—or treatment-as-usual (TAU) for 12 weeks. Intervention participants were instructed to record daily videos of buprenorphine self-administration. Study outcomes were: (1) percentage of the 12 weekly urine drug tests negative for illicit opioids with missing presumed positive (primary outcome) and (2) treatment retention at week 12 (secondary outcome). Poisson regression was used to estimate a risk ratio for no illicit opioid use calculated using Generalized Estimating Equations accounting for clusters. Retention rates were compared using Poisson regression with robust standard errors.

**Results:** Of 114 patients screened, 78 (68.4%) enrolled: 20 (25.6%) female; 30 (38.5%) non-white; 65 (83.3%) graduated high school; and 31 (39.7%) reported homelessness. The mean (SD) number of days on medication before study enrollment was 8.96 (± 7.34). The mean (SD)/median (IQR) for submitted videos was 31% (34%) and 16% (51%), respectively. In intention-to-treat analysis, the rate of weekly opioid negative UDT was 50% (95% CI 40–63%) in the intervention arm versus 64% (95% CI 55–74%) in the TAU arm; RR = 0.78 (95% CI 0.60–1.02, p = 0.07). Retention was similar in the intervention versus TAU arm, 69% (95% CI 56–86%) v. 82% (95% CI 71–95%); RR = 0.84 (95% CI 0.65–1.10, p = 0.20).

**Conclusion:** Video DOT for recently enrolled office-based buprenorphine patients for 12 weeks did not suggest benefits on illicit opioid use and treatment retention. However, its effectiveness was limited by low rates of use.

## A111 “Adaptation of a group-based community reinforcement and family training (CRAFT) approach with support persons of patients starting buprenorphine” (TD02)

### Jennifer K. Manuel, Karen Chan Osilla, Jarret Catlin, Brian Hurley, Barbara Lodge, Allison Ober, and Katherine Watkins

#### Lead Author Affiliation: University of California, San Francisco, School of Medicine, 4150 Clement St., San Francisco CA 94121, USA

##### **Correspondence:** Jennifer K. Manuel (jennifer.manuel@ucsf.edu)

*Addict Sci Clin Pract* 2020, **15(Suppl 2)**:A111

**Background:** The opioid epidemic continues to have devastating consequences. Buprenorphine is an effective treatment for opioid use disorder (OUD), but about half of patients drop out within the first year. Support persons (e.g., family members, close friends) of patients starting buprenorphine may encourage treatment retention. Community Reinforcement and Family Training (CRAFT) is an evidence-based approach developed as an individualized therapy for support persons (SP) of treatment-refusing substance users, but has not been extensively tested for OUD. We adapted CRAFT for support persons of patients starting buprenorphine and for delivery in a rolling, group format. To understand the feasibility and acceptability of this adaption, we assembled focus groups for feedback.

**Methods:** Our research team convened two focus groups: (1) patients with OUD in buprenorphine treatment and (2) SP of patients in buprenorphine treatment. Participants received $50 remuneration. Themes were gathered and summarized, and feedback was incorporated in the final version of the intervention.

**Results:** The patient group (N = 12 participants) highlighted the importance of their loved one understanding opioid addiction, buprenorphine benefits and side-effects, and how to administer Narcan. The SP group (N = 10 participants; partners, parents, grandparent, close friends) emphasized that the group filled a large gap in available clinical/support services for SPs and discussed the desire for more opioid education and training in communication. Perceived benefits of CRAFT included increased understanding of the patient’s perspective, the trajectory of addiction and role of buprenorphine in recovery, and emotional support for SPs from group members. Feedback was incorporated into an adapted 10-session CRAFT manual that is currently being piloted.

**Conclusion:** Focus group results suggest that group-based CRAFT may be a feasible and acceptable approach for broadening OUD treatment to include support persons. This approach has the potential to help retain patients in buprenorphine treatment while improving the SP and patient relationship.

## A112 “Building a pathway to treatment: pilot-test of family connect, a linkage-to-substance use treatment program for youth on probation” (TD03)

### Gail N. Robson, Jacqueline Lee, Jillian Watkins, Gail Wasserman, and Katherine Elkington

#### Lead Author Affiliation: Columbia University, 1051 Riverside Dr, New York, NY 10032, USA

##### **Correspondence:** Katherine Elkington (ke2143@cumc.columbia.edu)

*Addict Sci Clin Pract* 2020, **15(Suppl 2)**:A112

**Background:** Approximately 25%–50% of justice-involved youth (JIY) have substance use problems or disorders (SU/SUD), rates much higher than youth in the general population, yet 50%–80% of JIY do not receive SU services. Well-documented individual, family, and structural barriers must be addressed for JIY with SU/D to achieve linkage to services and ultimately, positive behavioral health outcomes. We developed and pilot-tested a unique service delivery model (Family CONNECT) that targets family- and systems-level factors to increase uptake of SU services among JIY.

**Methods:** We enrolled n = 18 youth on probation in need of SU treatment (56% male, age 14.49 years, 28% white, 44% Hispanic), and their caregivers, into Family Connect. Families worked with a linkage specialist (LS) to facilitate engagement in treatment; the LS also coordinated with probation officers and treatment providers. Referral, initiation (intake) and treatment engagement (intake + 1 session) of Family CONNECT youth was compared to a historical control group drawn from probation records using chi2 and multivariable logistic regression.

**Results:** Rates of referral [94% vs 74%, non-significant (ns)] and initiation (88% vs 71%, ns) to any behavioral health services were higher in Family CONNECT, while treatment engagement (56% vs 61%, ns) was slightly lower in Family CONNECT youth. On average, LS spent 90 days working with families to initiate treatment. Logistic regression models showed no demographic differences in initiation and engagement in care, and for referral only case type (JD vs PINS) was significant (OR = 3.3 [1.1, 9.8], p < 0.05) with more PINS referred for treatment.

**Conclusion:** Family Connect demonstrated promise of efficacy to successfully move JIY through the behavioral health care cascade, achieving increased referral and intake and similar levels of engagement. Use of a LS is a feasible approach to overcome both system- and youth/family-level factors to increase treatment uptake in JIY.

## A113 “Chronic opioid therapy: a scoping literature review on evolving clinical and scientific definitions” (TD04)

### Yun Shen, Hemita Bhagwandass, Tychell Branchcomb, Sophia A. Galvez, Ivanna Grande, Julia Lessing, Mikela Mollanazar, Natalie Ourhaan, Razanne Oueini, Michael Sasser, Ivelisse L. Valdes, Ashmita Jadubans, Josef Hollmann, Scott M. Vouri, Juan M. Hincapie-Castillo, Lauren E. Adkins, and Amie J. Goodin

#### Lead Author Affiliation: University of Florida, 1225 Center Drive Gainesville, FL 32610 HPNP Building, Rm 3334, USA

##### **Correspondence:** Yun Shen (yunshen@ufl.edu)

*Addict Sci Clin Pract* 2020, **15(Suppl 2)**:A113

**Background:** The management of chronic non-cancer pain (CNCP) with Chronic Opioid Therapy (COT) is controversial. Additionally, there is a lack of consensus on how COT is defined resulting in unclear clinical guidance and the evaluation of treatment effectiveness and safety. This scoping review identifies and evaluates evolving COT definitions throughout clinical and scientific literature.

**Methods:** The databases searched to identify clinical and scientific studies containing COT definitions including PubMed, Embase, and Web of Science. A total of 8866 studies published between January 2000 and July 2019 were screened with n = 224 meeting inclusion criteria. COT definitions in the literature were classified by pain population of application and specific dosage/duration definition parameters.

**Results:** Approximately half (50.5%) of studies defined COT as “days supply duration > 90 days” and 9.3% used a definition of “ > 120 days supply,” with other days supply cut-off points (> 30, > 60, or > 70) each appearing in < 5% of total studies. COT was defined by number of prescriptions in n = 63 studies, with 16.5% studies using number of initiations and 11.2% using number of refills. More than one-third (36.6%) studies included > 1 COT definition, and only 19.2% studies distinguished between acute/chronic pain treatment. Episode duration/dosage was used by 90 studies to define COT, with 7.59% by Morphine Milligram Equivalents (MME’s) + days’ supply and 32.59% by other “episode” combination definitions. COT definitions were applied to musculoskeletal CNCP (60.7%) most often, and typically in adults aged 18–64 (69.2%). The average number of studies per year defined COT using “> 90 days supply” increased from 3.2/year before 2016 to 20.7/year after 2016.

**Conclusion:** In recent years, an increasing proportion of studies defined COT as “> 90 days supply”. The most recent literature trends toward shorter duration criteria, suggesting that contemporary COT definitions are increasingly conservative.

## A114 “College students’ receptiveness to on- and off-campus prevention and treatment approaches for risky alcohol and cannabis use” (TD05)

### Ashley C. Helle, Cassandra L. Boness, and Kenneth J. Sher

#### Lead Author Affiliation: University of Missouri, 210 McAlester Hall, Columbia, MO 65211-2500, USA

##### **Correspondence:** Ashley C. Helle (hellea@missouri.edu)

*Addict Sci Clin Pract* 2020, **15(Suppl 2)**:A114

**Background:** Heavy alcohol and cannabis use are pervasive problems among college students. There has been a concerted effort to address this via empirical investigation and implementation of evidence-based prevention and treatment, though less work has focused on college students’ perceptions. Epler et al. (2009) identified that college students were most open to individual therapy and self-help options for reducing their alcohol use, but less open to medication. The current study provides a needed update on Epler’s work by examining student openness to intervention approaches across (1) a wider set of options given current technologies and climate (e.g., remote/telehealth), and (2) extending the focus to cannabis use.

**Methods:** 446 undergraduates reported on their alcohol and cannabis use, motives for and reasons against use, and openness to an array of intervention approaches for reducing alcohol and cannabis use.

**Results:** Students were most open to self-help options, talking with family/friends, individual approaches (therapy, PCP), and harm reduction/workshops specific to alcohol, and self-help, talking with family/friends, and individual approaches (therapy, PCP) specific to cannabis. In general, students were less open to groups and medication-based interventions. Women tended to express higher openness. Logistic multivariate regressions indicated that lower alcohol consumption and frequency of cannabis use were associated with increased openness to various approaches. Higher conformity (alcohol) motives were associated with more openness to specific intervention options. Those with greater risk of alcohol and cannabis dependence were less open to many intervention options.

**Conclusion:** College students are open to various intervention approaches for alcohol and cannabis, including technology-based approaches and talking with individual providers. These results can inform selection, implementation, and availability of campus-wide services as low-cost technological-based approaches are expanding. Further, institutions may consider further attention to existing services (e.g., peer support, PCP) for addressing alcohol and cannabis use, given students’ receptiveness to such approaches.

## A115 “Designing a user-informed prescription opioid misuse prevention program for juvenile justice-involved youth and caregivers” (TD06)

### Sarah A. Helseth, Gabriela Aisenberg, A. Rani Elwy, Sara J. Becker, Kathleen Kemp, and Anthony Spirito

#### Lead Author Affiliation: Brown University School of Public Health, 121 S Main St, Providence, RI 02903, USA

##### **Correspondence:** Sarah A. Helseth (sarah_helseth@brown.edu)

*Addict Sci Clin Pract* 2020, **15(Suppl 2)**:A115

**Background:** Prescription opioid (PO) misuse peaks in young adulthood, highlighting the need for effective PO misuse prevention programs during adolescence. Prevention needs may be greatest among youth in the juvenile justice (JJ) system, which is the largest single referrer for outpatient opioid misuse treatment, after self-referral. Surprisingly, no PO misuse prevention programs have been developed for youth in JJ-settings. We conducted formative research to explore the needs and preferences of JJ-involved families, to guide development of a JJ-tailored PO misuse prevention program.

**Methods:** Participants were recruited through the Rhode Island Family Court. Semi-structured interviews were conducted with 21 JJ-involved adolescents (M = 16 years [SD = 1]; 57% male; 52% Latinx; 43% Non-Hispanic White) and their caregivers (n = 20; 91% female; 36% Latinx; 45% Non-Hispanic White). Interviews assessed PO-related topics: information/knowledge, personal experiences, motivation, behavioral skills, and proposed program features, content, and design. Qualitative data analysis consisted of a hybrid inductive and deductive approach. Interview data were analyzed using a deductive, a priori coding framework developed from the Information Motivation Behavioral Skills model. Emergent, inductive codes that did not fit within this framework were also captured.

**Results:** We found that caregivers knew more about PO misuse than teens, several of whom misidentified non-opiates (e.g., Xanax, ecstasy) or substances containing opioids (e.g., cough syrup). Sample-wide, three caregivers and two adolescents reported personal PO misuse. Motivations for PO misuse included depression, life stressors, inability to access preferred substances, and experimentation. Both groups were unfamiliar with symptoms of PO misuse but eager to learn how to identify and handle a suspected overdose. Regarding the prevention program, all caregivers expressed interest in a brief session followed by text message support. Adolescents thought PO misuse prevention was not necessary because they “already know to stay away from that.”

**Conclusion:** These findings informed development of our PO misuse prevention program for JJ-involved families.

## A116 “Expanding outcomes when considering the relative effectiveness of two evidence-based outpatient treatment programs for adolescents” (TD07)

### Beth Ann Griffin, Lynsay Ayer, Joseph Pane, Brian Vegetabile, Lane Burgette, Daniel McCaffrey, Donna L. Coffman, Matthew Cefalu, Rod Funk, and Mark Godley

#### Lead Author Affiliation: RAND Corporation, 1776 Main St. Santa Monica, CA 90401-3208, USA

##### **Correspondence:** Beth Ann Griffin (bethg@rand.org)

*Addict Sci Clin Pract* 2020, **15(Suppl 2)**:A116

**Background:** The current study seeks to advance understanding about how to address substance use and co-occurring mental health problems in adolescents. Specifically, we compared the effectiveness of two evidence-based treatment programs (MET/CBT5 and A-CRA) for both substance use and mental health outcomes (i.e., crossover effects).

**Methods:** We utilized statistical methods designed to approximate randomized controlled trials when comparing nonequivalent groups using observational study data. Our methods also included an assessment of the potential impact of omitted variables.

**Results:** We found that after applying balancing weighting to ensure similarity of the baseline samples (given the non-randomized study design), both groups significantly improved on the two substance use outcomes (days abstinent and percent of youth in recovery) and on the two mental health outcomes (PTSD symptoms and general emotional problems). Youth in A-CRA were significantly more likely to be in recovery at the three-month follow-up compared to youth in MET/CBT5, but the size of this effect was very small. Youth receiving MET/CBT5 appeared to show significantly more improvement in the two mental health measures compared to youth in A-CRA, though these effect sizes were also very small.

**Conclusions:** The findings indicate that adolescents with co-occurring substance use and mental health problems appear to benefit from both treatments even though they are not specifically targeting mental health problems.

## A117 “Extended-release naltrexone and harm-reduction counseling: perception of treatment among people experiencing chronic homelessness with alcohol use disorder” (TD08)

### Kavya A. Magham, Emily Taylor, Roxanna King, Silvi Goldstein, Jessica Holttum, and Susan Collins

#### Lead Author Affiliation: Elson S. Floyd College of Medicine at Washington State University, 412 E Spokane Falls Blvd, Spokane, WA 99202, USA

##### **Correspondence:** Kavya A. Magham (kavya.magham@wsu.edu)

*Addict Sci Clin Pract* 2020, **15(Suppl 2)**:A117

**Background:** A recent randomized controlled trial of harm reduction counseling combined with extended-release naltrexone showed reductions in alcohol use and alcohol-related harm. Additionally, this combined pharmacobehavioral therapy demonstrated improvements in health-related quality of life. The objective of the present secondary study was to assess patient perceptions of this treatment through a qualitative analysis of exit interviews.

**Methods:** Participants were adults over 21 years of age experiencing homelessness and alcohol use disorder (AUD) recruited using consecutive sampling into a randomized controlled trial of harm reduction counseling and extended-release naltrexone. Included in the current sample were those who completed final Week 36 follow-up appointment (N = 155). Measures included participant responses to semi-structured interview prompts eliciting participants’ perspectives on the treatment components. A conventional content analysis was conducted by reviewing interview transcripts, systemically coding participants’ statements for each aspect of the study and classifying them into coding categories.

**Results:** Participants reported interest in the study due to desire to change their drinking habits, the payment, and interaction with staff. Preliminary results suggest general satisfaction with study assessments and counseling, including feeling heard by the staff and learning valuable information about healthy drinking habits. There were mixed perceptions of extended-release naltrexone. While some participants thought it helped recover from heavy alcohol drinking, others expressed discomfort with the injection site and the fear of needles.

**Conclusions:** Participants generally reported positive experiences with patient-centered counseling and provided constructive criticism on how to improve medical and medication-related procedures. These findings contribute to patient-centered research by providing a means for patients to shape treatment development and enhancement for marginalized individuals with alcohol dependence.

## A118 “Health and economic outcomes of treatment with extended-release naltrexone among pre-release prisoners with opioid use disorder (HOPPER)” (TD10)

### Sean M. Murphy, Philip J. Jeng, Sabrina A. Poole, Ali Jalali, Danielle A. Ryan, Frank J. Vocci, Michael S. Gordon, George E. Woody, and Daniel Polsky

#### Lead Author Affiliation: Weill Cornell Medical College, 1300 York Ave, New York, NY 10065, USA

##### **Correspondence:** Danielle A. Ryan (Dar4006@med.cornell.edu)

*Addict Sci Clin Pract* 2020, **15(Suppl 2)**:A118

**Background:** Opioid-overdose is the leading cause of death among previously-incarcerated persons with opioid use disorder (OUD), with the first post-release weeks proving especially fatal. Extended-release naltrexone (XR-NTX) ensures ~ 30 days of opioid-overdose protection. The drug’s high cost is a barrier; however, benefits/cost-offsets associated with effective treatment could improve budgets, and society as a whole.

This protocol describes a NIDA-funded study to—evaluate changes in healthcare utilization, quality-of-life, and other resources associated with different strategies of XR-NTX delivery to persons with OUD being released from incarceration; and estimate the relative “value” of each strategy.

**Methods:** Data from two ongoing, randomized-controlled trials will be used to evaluate these questions, within and across studies. Study A (XR-NTX Before vs. After Reentry) compares pre-release injection + treatment referral, vs. referral only. Study B (XR-NTX vs. enhanced XR-NTX) compares pre-release injection + referral, vs. pre-release injection + post-release place-of-residence treatment.

**Results:** Trials are ongoing. We will produce four outcomes: (1) estimate the cost of the correctional health system of implementing and running each XR-NTX program, (2) evaluate the differences in the utilization of healthcare services associated with opioid use across the different arms, (3) evaluate differences in QALYs gained across arms, (4) conduct a cost-effectiveness analysis via the incremental cost-effectiveness ratio (ICER).

**Conclusions:** This study offers the unique opportunity to assess the effectiveness and cost-effectiveness of multiple XR-NTX delivery strategies, according to different stakeholder perspectives.

## A119 “Help me help my teen after residential substance use treatment: a content analysis of parent forum posts” (TD11)

### Sarah A. Helseth, Kelli Scott, Katherine I. Escobar, Frances Jimenez, and Sara J. Becker

#### Lead Author Affiliation: Brown University School of Public Health, 121 S Main St, Providence, RI 02903, USA

##### **Correspondence:** Sarah A. Helseth (sarah_helseth@brown.edu)

*Addict Sci Clin Pract* 2020, **15(Suppl 2)**:A119

**Background:** Adolescents in residential substance use (SU) treatment have extremely high risk of relapse following discharge. Continuing care services can reduce youth relapse rates, but families often encounter logistical barriers that impede their ability to obtain these services. Mobile health (mHealth) technologies have increased teens’ access to continuing care services, but no comparable mHealth programs exist to support their parents during the post-discharge transition home. We developed Parent Substance Misuse in Adolescents in Residential Treatment (SMART), a mHealth program that combined one-on-one coaching sessions, a computerized parenting skills program, and an app-based networking forum.

**Methods:** To gain insight into parents’ post-discharge needs, we conducted a content analysis of forum posts made during the Parent SMART pilot trial. Thirty parents (87% female, 73% Non-Hispanic White) of teens (ages 12–18) in residential SU treatment had access to two expert-moderated forums: Ask an Expert (AAE) gave parents rapid access to an adolescent SU expert, while Connect with Parents (CWP) facilitated peer support among participants. Data were analyzed using thematic analysis.

**Results:** Twenty-one parents (70%) posted in either forum; of those, 9 posted in both. Twelve unique users made 15 AAE posts and 18 unique users made 50 CWP posts. Five major themes emerged: parent-to-parent support (27 posts), parenting skills (24 posts), post-discharge transition (13 posts), adolescent SU (9 posts), and family functioning (7 posts). AAE posts most-often sought consultation on Parent SMART skills (61% of AAE content). In contrast, CWP posts sought recommended strategies for implementing skills (21%) or support around managing a teen with SU problems (40%).

**Conclusion:** Our analysis suggests that parents solicit both expert and peer support during their teen’s post-discharge transition home. Implications for future mHealth continuing care programs to help parents help their teens will be discussed.

## A120 “Helping me to help you: does a web-based CRAFT intervention change drinking in the concerned partner?” (TD12)

### Lindsey M. Rodriguez, Karen Chan Osilla, Clayton Neighbors, and Eric R. Pederson

#### Lead Author Affiliation: University of South Florida, 4202 E. Fowler Avenue, Tampa, FL 33620, USA

#### **Correspondence:** Lindsey M. Rodriguez (lrodriguez12@usf.edu)

*Addict Sci Clin Pract* 2020, **15(Suppl 2)**:A120

**Background:** Military service members and their partners report greater alcohol use and related problems compared to their civilian counterparts. Community reinforcement and family therapy (CRAFT) is a therapeutic approach for individuals who are concerned about their loved one’s substance use (i.e., concerned partners; CPs). CRAFT focuses on enhancing self-care for the CPs and improving partner communication between the two partners, as well as increasing help-seeking and reducing drinking for the heavy drinking loved one. However, CPs may present with heavy drinking themselves, which may affect the effectiveness of CRAFT. To date, no research has explored whether CRAFT may also reduce drinking in the CP. We evaluated a web-based CRAFT intervention on reductions in CP drinking, and whether intervention effects differed as a function of CP baseline drinking levels.

**Methods:** Military CPs were recruited online and randomized to either web-based CRAFT called Partners Connect or a delayed waitlist control condition, and followed for 5 months. CPs (N = 161) who reported drinking at least once in the past month were included in analysis.

**Results:** Negative binomial regression models were used with CP drinking frequency as the outcome, with covariates including baseline CP drinking frequency, number of children, age, and perceived partner drinking. Results indicated that the intervention was not associated with overall changes in CP drinking frequency. However, CP drinking at baseline moderated the intervention’s effect, such that it was efficacious in reducing CP drinking frequency over time among CPs who were heavier drinkers at baseline (i.e., those who drank 15 drinks per week or more).

**Conclusion:** CPs participated in a study to help their loved one and we found that this web-based intervention served as an additional opportunity to help themselves as well, and may offer a unique opportunity to help dyads who may benefit from a brief intervention.

## A121 “Qualitative evaluation of two alcohol cessation interventions for elective surgical patients” (TD13)

### Diana Diaz Martin, Lyndsay Chapman, Tom Ren, Michael J Mello, Brian Borsari, Frederic C. Blow, and Anne C. Fernandez

#### Lead Author Affiliation: Michigan State University College of Human Medicine, 965 Fee Rd A110, East Lansing, MI 48824, USA

##### **Correspondence:** Diana Diaz Martin (dianadm@umich.edu)

*Addict Sci Clin Pract* 2020, **15(Suppl 2)**:A121

**Background:** High risk alcohol use prior to surgery is a common surgical risk factor associated with an increased risk of postoperative complications and prolonged hospital stay. However, pre-surgical patients are inconsistently screened for alcohol problems and rarely offered advice or alcohol intervention preoperatively. This study tested two alcohol cessation interventions to assist patients in reducing alcohol use. The interventions aim to increase alcohol abstinence before and after elective surgery with the goal of decreasing post-operative complications.

**Methods:** This study tested two preoperative alcohol interventions: (1) Health Coaching; a two-session motivational interview delivered by a health coach, and (2) Brief Advice; a 10-min session that could be delivered by clinic staff. Elective surgical patients who met ‘risky drinking’ criteria (N = 12) took part in the intervention trial during which they provided qualitative and quantitative feedback to further refine the intervention content and design.

**Results:** Data indicated that patients preferred interventions emphasize surgical health promotion messages rather than addiction messaging. Patients emphasized the need for visually appealing infographics, tailored data-based feedback, and a flexible delivery platform. Quantitative data from the open trial reflected high need, importance, and acceptability of the intervention and content.

**Conclusions:** This study resulted in patient feedback and data that was used to further refine and improve two preoperative alcohol interventions for elective surgical patients. The interventions are currently undergoing evaluation through a randomized pilot trial. They have the potential to address gaps in alcohol screening and intervention in preoperative surgical care.

## A122 “Peer-delivered recovery support services in addiction treatment: a scoping review” (TD14)

### Lauren Perron, and Steven Belenko

#### Lead Author Affiliation: Department of Criminal Justice at Temple University, 1115 Polett Walk, Philadelphia, PA 19122, USA

##### **Correspondence:** Lauren Perron (tuj68329@temple.edu)

*Addict Sci Clin Pract* 2020, **15(Suppl 2)**:A122

**Background:** There have been increased efforts to incorporate peer-based recovery services into existing models of substance abuse treatment, as recovery-oriented, chronic care approaches to addiction treatment have grown in popularity. However, the expanded use of the peer model has occurred in the absence of clear empirical data on its effectiveness.

**Methods:** This review considers studies that examined interventions involving a peer recovery support component and their effectiveness in terms of treatment engagement, drug abstinence, health service utilization, and other related outcomes. A combination of search terms was used to locate relevant literature from a variety of databases. These terms included “peer-based recovery services”, “peer recovery support specialists”, “peer recovery coaches”, “effectiveness of”, and “recovery outcomes associated with”. Articles examining mutual aid modalities of peer support were excluded from the review.

**Results:** A total of twenty-nine peer-reviewed studies met the criteria for inclusion in this review. Several studies suggest that peer recovery services are associated with higher rates of drug abstinence, across many different drug types. Individuals receiving peer recovery support services overwhelmingly show greater treatment engagement, with a higher likelihood of attendance, more frequent attendance, satisfaction, and faster initiation to treatment. The results show mixed effects of the effectiveness of peer recovery support on health service utilization. Other studies found no significant differences between peer recovery services and clinical treatment alone on different outcomes.

**Conclusions:** Though there is promising evidence of the benefits of peer-delivered recovery support services, there is a need to expand this literature base. Much of the extant literature lacks methodological rigor, and few studies have examined the direct effect of peer recovery support on recovery outcomes. New research is needed to disentangle the effects of peer support from those of the treatment intervention itself.

## A123 “Postpartum risky drinking: a survey to inform development of a text messaging intervention” (TD15)

### Sarah Dauber, Cori Hammond, Michelle Martinez, Johannes Thrul, and Allison West

#### Lead Author Affiliation: Center on Addiction, 485 Lexington Ave, New York, NY 10017, USA

##### **Correspondence:** Sarah Dauber (sdauber@centeronaddiction.org)

*Addict Sci Clin Pract* 2020, **15(Suppl 2)**:A123

**Background:** Postpartum risky drinking (RD) is associated with long-term negative child outcomes. Postpartum women are at high risk for alcohol relapse, as more than half of women who reduce their drinking during pregnancy return to pre-pregnancy levels within 3 months postpartum. Postpartum women are unlikely to seek formal treatment for RD due to stigma and fears of child removal. Text messaging interventions (TMIs) show promise for improving reach to address postpartum RD and prevent negative outcomes. This study assessed feasibility, acceptability, and utility of a TMI to address postpartum RD.

**Methods:** We conducted a Qualtrics panel survey of 170 low-income postpartum women. Forty-four respondents (26%) were defined as risky drinkers (reported any binge drinking during pregnancy, or reported binge drinking monthly or more often before pregnancy or postpartum), and were compared to the remaining 126 women on concern about drinking, technology use behaviors, and interest in a TMI for RD.

**Results:** Risky drinkers were significantly more likely than others to report being concerned about their drinking since giving birth (p = 0.001). Risky drinkers were frequent users of mobile phones, with 86% sending or receiving text messages and 91% checking for text messages at least daily. Forty-three percent of risky drinkers reported that they were very or extremely likely to participate in a TMI for RD. Most frequently endorsed barriers to participation among risky drinkers included worry about the messages being annoying (43%) and fear of child removal (48%), which was a significantly greater concern among risky drinkers compared to non-risky drinkers (p = 0.004).

**Conclusions:** Results support the feasibility of and interest in a TMI for RD in postpartum women, and also suggest particular areas of concern for this population to inform TMI development.

## A124 “Technology-based tools and resources for substance use disorders” (TD16)

### Zach Sneed, Amy Faltinek, Regina Baronia, and Peggy Edwards

#### Lead Author Affiliation: Texas Tech University Health Sciences Center, 3601 4th Street, Lubbock TX 79430, USA

##### **Correspondence:** Zach Sneed (zach.sneed@ttuhsc.edu)

*Addict Sci Clin Pract* 2020, **15(Suppl 2)**:A124

**Background:** With increasing sophistication and access to Internet technology, there is a corresponding increase in web-based treatments for substance use disorders (SUD). Advantages of technology-based treatment for SUD can include: greater access to treatment, screening and referral, personalized materials and effects, social support, therapeutic prompts and monitoring among others. Also, the use of technology greatly increases the kind and amount of data useful to researchers. Our study took a more in depth review on a wider range of technology-based SUD treatment interventions.

**Method:** We conducted a content analysis via systematic review methods using PubMed, Embase, Cochrane, CINAHL, Ageline, PsycInfo, Rural Health Information Hub, WorldWideScience.org, ClinicalTrials.gov, Scopus and Web of Science. This process returned 3222 references which were imported into Covidence for review. Removing 396 duplicates left 2,826 studies. Next, irrelevant 2102 articles were excluded, leading to 720 full text reviews. This phase included interlibrary loans, interrater reliability, and conflict resolution strategies excluding 301 articles. Final results are based on the full-text review of 410 studies.

**Results:** Final results include a wide array of information such as article focus (research or conceptual); type of research (clinical trial, group experiment, single-subject, systematic reviews); and a summary of the included studies’ research questions and/or specific aims.

**Conclusion:** A significant variety of technology-based SUD tools and resources exist; however much of the recent literature struggles to use common definitions necessary for progress. Some products have existed for years and possess a substantial evidence base; however other research presented in final form is lacking typical components of published work relat3ed to methodology and efficacy. A variety of opportunities exist to expand or augment clinical services and research. Our presentation provides a methodologically rigorous and relevant synopsis.

## A125 “The acceptability and feasibility of smartphone- based recovery coaching and contingency management to reduce substance use among young adults” (TD17)

### Win C. Turner, and Jody L. Kamon

#### Lead Author Affiliation: Center for Behavioral Health Integration at Stony Brook University, PO Box 966, Middlebury, VT 05753, USA

##### **Correspondence:** Win C. Turner (wincturner@gmail.com)

*Addict Sci Clin Pract* 2020, **15(Suppl 2)**:A125

**Background:** Screening and intervening with young adults to reduce substance use including nicotine is a major healthcare problem. Contingency management (CM) is a highly effective but rarely used method to reduce substance use. Young adults are known to be fluent in online applications and may be more interested in a technology based CM approach for reducing their substance use. To examine the feasibility and acceptability of an app-based substance use intervention utilizing CM to increase engagement and deliver evidence-based interventions for risky substance misuse among a college population.

**Methods:** Through a college health center’s SBIRT efforts, students identified with risky substance misuse (alcohol, nicotine, marijuana, other drugs) were offered the opportunity to sign up for smartphone-based CM including (a) “facetime” (b) recovery coaching, (c) blue tooth substance monitoring, (d) healthy activities and (e) “smart bank” incentives. A unique aspect was the acceptance of student chosen substance goals as targets for enhancing engagement, internal motivation & CM incentives. Findings presented include fourteen students engaged while living on a college campus as well as their time post COVID-19 living off campus.

**Results:** Twelve of 14 young adults continue to be engaged in Dynamicare three months post-enrollment. Evaluation data included monthly self-report of functioning and toxicological testing. Ratings on ease of use, helpfulness, and satisfaction were high with 75% to 100% of participants reporting strong satisfaction depending on the indicator. Based on self-report and testing data, participants reduced their nicotine use. Data will also be presented on alcohol, cannabis use, depression and anxiety.

**Conclusion:** Smartphone based CM with "facetime and/or text" recovery coaching is not only feasible but also acceptable to young adults. Reductions in nicotine use occurred with little change in alcohol and cannabis. Future directions should consider how both self-selection of reduction goals & incentive values impact outcomes.

## A126 “Screening, self-management, and referral to treatment (SSMRT): a secondary prevention platform for populations without access to care” (TD18)

### Karen T. Y. Tang, Alexandra Loverock, Jakob Koziel, and Igor Yakovenko

#### Lead Author Affiliation: Dalhousie University, 6299 South St, Halifax, NS B3H 4R2, Canada

##### **Correspondence:** Karen T. Y. Tang (karen.tang@dal.ca)

*Addict Sci Clin Pract* 2020, **15(Suppl 2)**:A126

**Background:** Few individuals with substance use problems ever access addiction health services. This is exacerbated by the lack of mental health solutions across North America due to limited treatment spaces and preference to personally manage use. Past research has found that screening, brief intervention, and referral to treatment is efficacious and has potential for addressing this gap. We sought to develop and implement a remotely accessible platform called the Screening, Self Management, and Referral to Treatment (SSMRT). SSMRT is a marijuana misuse resource aimed at youth and young adults who may not have access to traditional addiction health services.

**Methods:** The platform was based on: (a) scoping review of 2473 studies that used brief screeners and 468 studies of brief interventions for substance use, (b) establishing a reference group of 36 researchers and front-line service providers, and (c) online survey of 3600 university students' marijuana use habits and interest in specific types of online supports for their substance use concerns.

**Results:** The SSMRT platform is a free, online intervention designed to increase access to mental health resources and supports for young adults across North America. SSMRT allows clinicians, marijuana users, family members of users, and researchers to take advantage of its resources remotely. This presentation will provide an overview of the development of the SSMRT platform and the results of the survey on youths’ substance use habits. Notably, between 26 and 51% of past 6-month marijuana users indicated that they would be 'very interested' or 'definitely would' access screening tools to determine their marijuana use, general information on marijuana, interactive tools to help them manage substance use problems, and locally available treatment resources.

**Conclusion:** Limitations of primary prevention approaches, and opportunities for widespread dissemination will be discussed as they relate to the implementation of the SSMRT platform.

## A127 “The whole health study RCT protocol: collaborative care treatment for opioid use disorder and co-occurring psychiatric disorders in primary care” (TD19)

### David S. Mandell, Hillary R. Bogner, Kyle M. Kampman, and The Whole Health Study Team

#### Lead Author Affiliation: University of Pennsylvania Perelman School of Medicine, 3400 Civic Center Blvd, Philadelphia, PA 19104, USA

##### **Correspondence:** David S. Mandell (mandelld@upenn.edu)

*Addict Sci Clin Pract* 2020, **15(Suppl 2)**:A127

**Background:** People with opioid use disorder (OUD) often have a psychiatric disorder, which elevates the risk of morbidity and mortality. Promising evidence supports the use of collaborative care for treating people with OUD in primary care. Whether collaborative care interventions that treat both OUD and psychiatric disorders will result in better outcomes is presently unknown.

**Methods:** The Whole Health Study is a randomized controlled trial designed to test collaborative care treatment for OUD and the psychiatric disorders that commonly accompany OUD. Approximately 1,200 University of Pennsylvania Health System primary care patients aged 18 years or older with OUD and depression or anxiety will be randomized to one of three conditions: (1) Augmented Usual Care, which consists of a primary care physician (PCP) waivered to prescribe buprenorphine, a mental health care manger, and an addiction psychiatrist to consult on medication-assisted treatment; (2) Collaborative Care, which consists of a waivered PCP, a mental health care manager with OUD and mental health treatment training, and an addiction psychiatrist who provides consultation for OUD and mental health; or (3) Collaborative Care Plus, which consists of all the elements of the Collaborative Care arm plus a Certified Peer Recovery Specialist to help with patient engagement in treatment and retention. **Results:** Primary outcomes are 6-month rates of opioid use and 6-month rates of remission of co-occurring psychiatric disorders. Secondary outcomes are buprenorphine adherence, treatment retention, use of non-prescribed drugs, and mortality. We also will assess the costs of delivering care in each study arm and the changes in total healthcare costs among participants.

**Conclusion:** The Whole Health Study will investigate whether collaborative care models that address OUD and co-occurring depression or anxiety will result in better patient outcomes in primary care. The results will inform public health policy and clinical care delivery in the context of the current opioid crisis.

## A128 “Transporting to treatment: the effectiveness of a mobile engagement unit” (TD20)

### Rebecca E. Stewart, Lisa Shen, Josh Vigderman, Merakey; Nayoung Kwon, Molly Candon, David Mandell, Susanna Kramer, Roland Lamb, and Aileen Rothbard

#### Lead Author Affiliation: University of Pennsylvania Perelman School of Medicine, 3400 Civic Center Blvd, Philadelphia, PA 19104, USA

##### **Correspondence:** Rebecca E. Stewart (rebecca.harris@pennmedicine.upenn.edu)

*Addict Sci Clin Pract* 2020, **15(Suppl 2)**:A128

**Background:** Many novel engagement and low-threshold treatment services (such as mobile treatment units) have been developed to meet the needs of people with opioid use disorder (OUD). Use of these service models has outpaced the research on their effectiveness. The current study examines the effectiveness of a mobile engagement unit in connecting individuals with OUD to treatment.

**Methods:** This retrospective cohort study compared demographic, prior treatment characteristics and service outcomes for individuals transported to an appointment intake by a Mobile Engagement Unit (MEU) with those of individuals who came to the intake through typical referral routes such as walk-in, other providers, and court order. Comparisons of baseline demographics and comorbidities were conducted using χ^2^ tests. We used a difference-in-differences approach and propensity score matching to examine use of outpatient and methadone maintenance services.

**Results:** The sample includes 468 individuals who had an intake between October 1, 2018 and March 31, 2019. Fifty-three (11%) came to treatment via MEU, while 415 came to treatment through other referrals. The two groups were balanced in sex, age, mental health and substance use diagnostic acuity. There were statistically significant differences in race, with the mobile group serving more white individuals. MEU participants used fewer conventional outpatient and methadone services than the comparison group prior to intake, and used more services following the intake.

**Conclusion:** A mobile transport program effectively connected individuals to SUD treatment, particularly in the first month. This is promising given many high-risk individuals do not typically engage in substance use treatment services. Future research is needed to understand how the unit “jump started” treatment.

## A129 “Evaluating the effects of a cognitive behavioral therapy intervention on adults that use cannabis on first-time alcohol-involved DUI offenses” (TD21)

### Bryce Pardo, Karen Chan Osilla, and Rosanna Smart

#### Lead Author Affiliation: RAND Corporation, 1776 Main St. Santa Monica, CA 90401-3208, USA

##### **Correspondence:** Bryce Pardo (brycepardo@gmail.com)

*Addict Sci Clin Pract* 2020, **15(Suppl 2)**:A129

**Background:** Co-use of alcohol and cannabis may result in greater driving impairment than use of either substance alone. Many individuals arrested for driving under the influence (DUI) are mandated to attend a program for license reinstatement, but these programs have largely focused on alcohol-involved DUI. Little is known about their effectiveness in reducing DUI among those that co-use both drugs.

**Methods:** We examined survey data from a randomized clinical trial of 322 participants enrolled in three DUI programs in California. Participants were 21 and older with a first‐time DUI offense. Participants were randomly assigned to a 12‐session cognitive behavioral therapy (CBT) focused on reducing alcohol-involved DUI, or usual care (UC) and then surveyed 4 and 10 months later. We conducted intent‐to‐treat analyses to test the hypothesis that alcohol-focused interventions may not be as effective for individuals who use cannabis.

**Results:** Participants were 72.3% male and 51.7% Hispanic, with an average age of 33.2 (SD = 12.4); 81% reported alcohol use in the past month; 38% reported past-month cannabis use. Relative to UC, participants receiving CBT had lower odds of driving after drinking at follow‐ups compared to participants receiving UC (odds ratio [OR] = 0.33, p < 0.1, and OR = 0.27, p < 0.1, at 4- and 10-month follow-ups, respectively) after controlling for past-month cannabis use. The intervention was not moderated by self-reported use of cannabis (OR = 1.26, p = 0.08; OR = 1.51, 0.63, at 40 and 10-month follow-ups, respectively).

**Conclusions:** While CBT helped reduce alcohol-involved DUI in both individuals who used alcohol-only and alcohol and cannabis, the intervention did not extend to cannabis-involved driving outcomes or past-month days of cannabis use. Cannabis-involved DUI and other related behaviors may benefit from specific interventions that seek to understand the mechanism underlying their use and subsequent driving behavior. This is increasingly important as states relax cannabis prohibition.

