# Correction to: Proceedings of the Addiction Health Services Research (AHSR) 2020: Virtual Conference: Part 2

**DOI:** 10.1186/s13722-021-00217-x

**Published:** 2021-03-02

## Correction to: Addict Sci Clin Pract (2020) 15(Suppl 2):35 https://doi.org/10.1186/s13722-020–00208-4

After publication of this supplement [1], it was brought to our attention that errors were apparent in the following abstracts.

## A1 “Patient characteristics associated with admission to low-safety inpatient psychiatric facilities: evidence of racial inequities” (AW01)

Morgan C. Shields

Lead Author Affiliation: Center for Mental Health, Department of Psychiatry, Perelman School of Medicine, University of Pennsylvania, 3400 Civic Center Blvd, Philadelphia, PA 19,104, USA

***Correspondence: ***Morgan C. Shields (shmorg@upenn.edu)

Correction:

The text of the abstract is incorrect. The abstract text should read:

**Background:** I examined patient demographic, clinical, payment, and geographic factors associated with admission to low-safety inpatient psychiatric facilities.

**Methods: **Massachusetts all-payer 2017 discharge data (N = 39,128) were linked to facility-level indicators of safety (N = 38). A composite of safety performance was constructed by averaging standardized measures of restraint and seclusion, as well as five-year (2014–2018) averages of overall, substantiated, and abuse-related (verbal, physical, sexual abuse) complaints per 1,000 discharges (• = 0.73). This composite informed the grouping of high (top 20%), middle, and low-safety (bottom 20%) performers. I first examined unadjusted differences across safety groups, as well as differences in bypass patterns across racial and ethnic groups. I then fit a series of multinomial regression models, adding payment and geography separately.

**Results:** Outstanding factors independently associated with admission to low-safety facilities were being a racial or ethnic minority compared to White patient (relative risk ratio [RRR] for non-Hispanic Black = 1.7, 95% CI = 1.5–2.0; non-Hispanic Asian = 5.6, 95% CI = 3.6–8.7; non-Hispanic “other” race = 2.2, 95% CI = 1.7–2.7; Hispanic/Latinx = 1.3, CI = 1.1–1.5), and not having private insurance (RRR for uninsured/self-pay = 2.4, CI = 1.6–3.6, Medicaid = 1.8, CI = 1.6–2.0, Medicare = 1.3, CI = 1.2–1.5). Several other factors were independently associated with admission to low-safety facilities, such as substance use disorder other than alcohol, proximity, severity, schizophrenia/psychosis, homelessness, and younger age.

**Conclusion:** There were considerable racial and ethnic inequities in admission to low-safety inpatient psychiatric facilities even after accounting for clinical, geographic, and payment characteristics. Future research should further examine quality variation and outcomes, as well as how community-based referrals, mode of transport (e.g., police, self), and deliberate steering and selection affect admissions and outcomes.

The original paper has been updated.

## A53: “A Multidisciplinary Outreach Team Approach to Treatment Engagement in Medication for Opioid Use Disorder” (IS03)

Courtney M. DelaCuesta, BS*; Rebecca Uth, PsyD; Linda Hurley, MA, CAGS, LCDCS; and Rosemarie Ann Martin, PhD.

Lead author affiliation: Brown University School of Public Health, 121 S Main St, Providence, RI 02903, USA

*Correspondence: Courtney DelaCuesta* (Courtney_DelaCuesta@brown.edu)

Correction:

The title of the abstract is incorrect. The title should read:

A Multidisciplinary Outreach Team Approach to Treatment Engagement in Medication for Opioid Use Disorder

## A75 “Examining preliminary data for opioid‑related technical assistancerequests for underserved communities” (MM08)

Holly Hagle, Michael Knabel, Yifei Liu, Frances Bozsik, Ignacio Alex Barajas Munoz, Laurie Krom, Aimee Campbell, Kathryn Cates‑Wessel, and Frances Levin.

Lead Author Affiliation: University of Missouri, Kansas City, 5000 Holmes St, Kansas City, MO 64,110, USA.

***Correspondence: ***Holly Hagle (hagleh@umkc.edu).

Correction:

The lead author of the abstract should be Holly Hagle, Ph.D., and corresponding author should be: Correspondence: Holly Hagle (hagleh@umkc.edu), the original paper has been updated.

## A100: “Operationalizing Person-Centered Care in Behavioral Health Treatment: How can Treatment Centers Implement “Respect for Patients’ Values, Preferences, and Expressed Needs?” A Qualitative Study” (SW13)

Olivia Randall-Kosich, MHA*; Barbara Andraka-Christou, JD, PhD; Rachel Totaram, MHA; Kendall Cortelyou-Ward, PhD; Olena Mazurenko, MD, PhD; Danielle Atkins, PhD; and Andriy Koval, PhD.

Lead Author Affiliation: Georgia State University School of Public Health, 140 Decatur St SE, Atlanta, GA 30303, USA

*Correspondence: Olivia Randall-Kosich* (orandallkosich1@gsu.edu)

Correction: An author was left out of the original author list. The author that was left out is:

Olena Mazurenko, MD, PhD

## A126: “Screening, Self-Management, and Referral to Treatment (SSMRT): A Secondary Prevention Platform for Populations Without Access to Care” (TD18)

Karen T. Y. Tang, BA*; Alexandra Loverock, MSc; Jakob Koziel, MSc; Cameron Wild, PhD, and Igor Yakovenko, PhD.

Lead Author Affiliation: Dalhousie University, 6299 South St, Halifax, NS B3H 4R2, Canada

*Correspondence: Karen Tang* (karen.tang@dal.ca)

Correction: An author was left out of the original author list.

The author that was left out is: Cameron Wild, PhD
